# Supplementary material for: Breast cancer secretes anti-ferroptotic MUFAs and depends on selenoprotein synthesis for metastasis
Source: EMBO Mol Med. 2024 Oct 21;16(11):7. doi: 10.1038/s44321-024-00142-x (PMC11555046; doi:10.1038/s44321-024-00142-x)
Supplement: Supplementary file 1 — Appendix [file 44321_2024_142_MOESM1_ESM.pdf]

Table of content:

Appendix Figure S1. (p. 1)

Appendix Figure S2. (p.2)

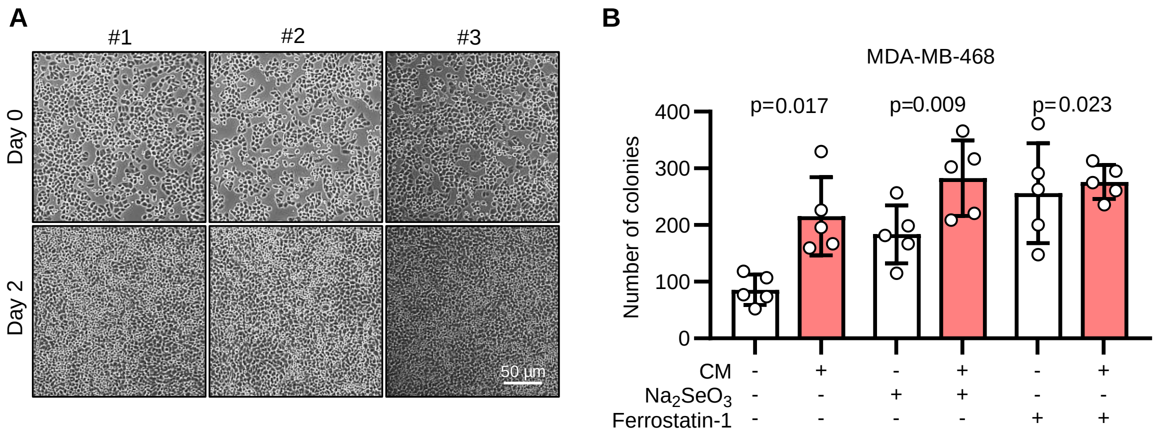

Appendix Figure S1: Medium conditioned by breast cancer cells and cancer-associated fibroblasts enhances the clonogenicity of triple-negative breast cancer cells.

A. Images of high-density cultures of MDA-MB-468 cells at the start (day 0) and end (day 2) of the medium conditioning period. Representative fields of view from 3 independent experiments (#1-3) are shown.

B. Quantification of the number of colonies obtained from the assays shown in Figure 1C. P values refer to a two-way ANOVA test for unpaired samples with Dunnett's multiple comparisons test. n_{exp}=5. Bars represent mean ± s.d.

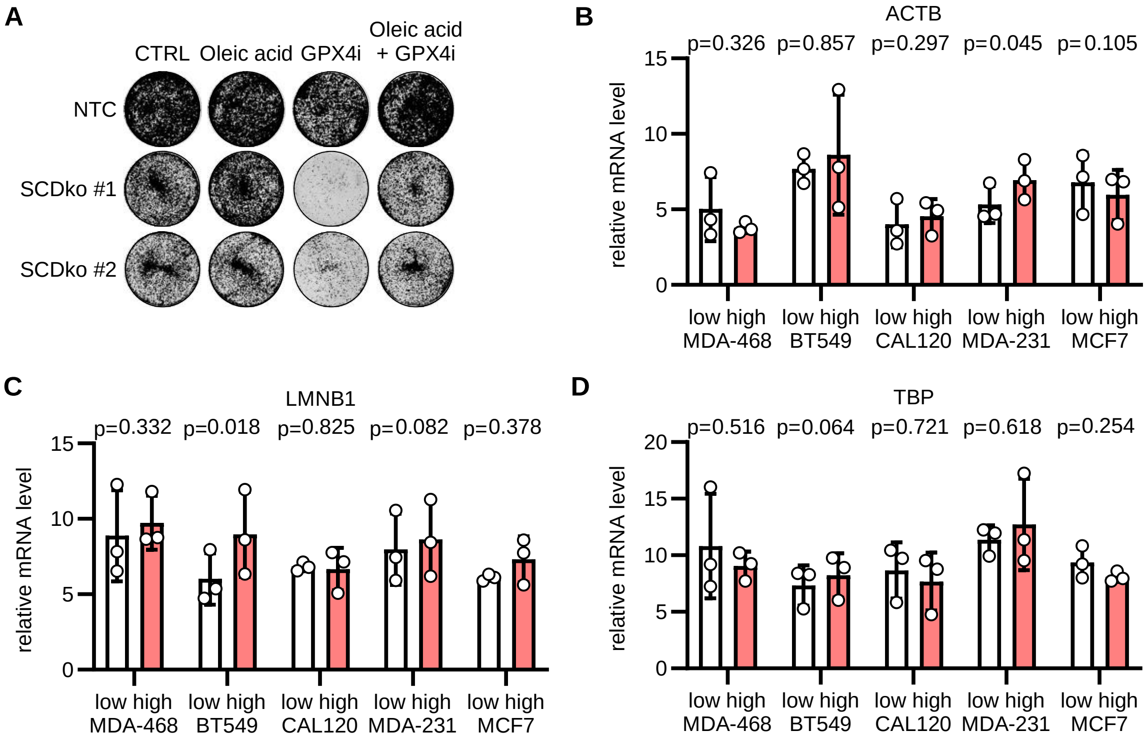

Appendix Figure S2: Loss of SCD sensitises cells to ferroptosis.

A. Representative images of the colony forming assays shown in Figure 5B.

B-D. qPCR quantification of ACTB (B), LMNB1 (C), and TBP (D) mRNA expression in MDA MB-468, BT549, CAL120, MDA-MB-231 and MCF7 cells seeded at low or high density and cultured for 2 days. The mean values for the three genes were used to normalize the expression of the genes shown in Figure 5 H. P value refers to a two-tailed, homoscedastic Student's *t* tests for paired samples comparing low and high densities. Bars represent mean ± s.d.
