# Supplementary material for: Breast cancer secretes anti-ferroptotic MUFAs and depends on selenoprotein synthesis for metastasis
Source: EMBO Mol Med. 2024 Oct 21;16(11):7. doi: 10.1038/s44321-024-00142-x (PMC11555046; doi:10.1038/s44321-024-00142-x)
Supplement: Supplementary file 2 — Source data Fig. 1 [file 44321_2024_142_MOESM2_ESM.zip › Figure 1/C/picture+label.pptx]

## Slide 1
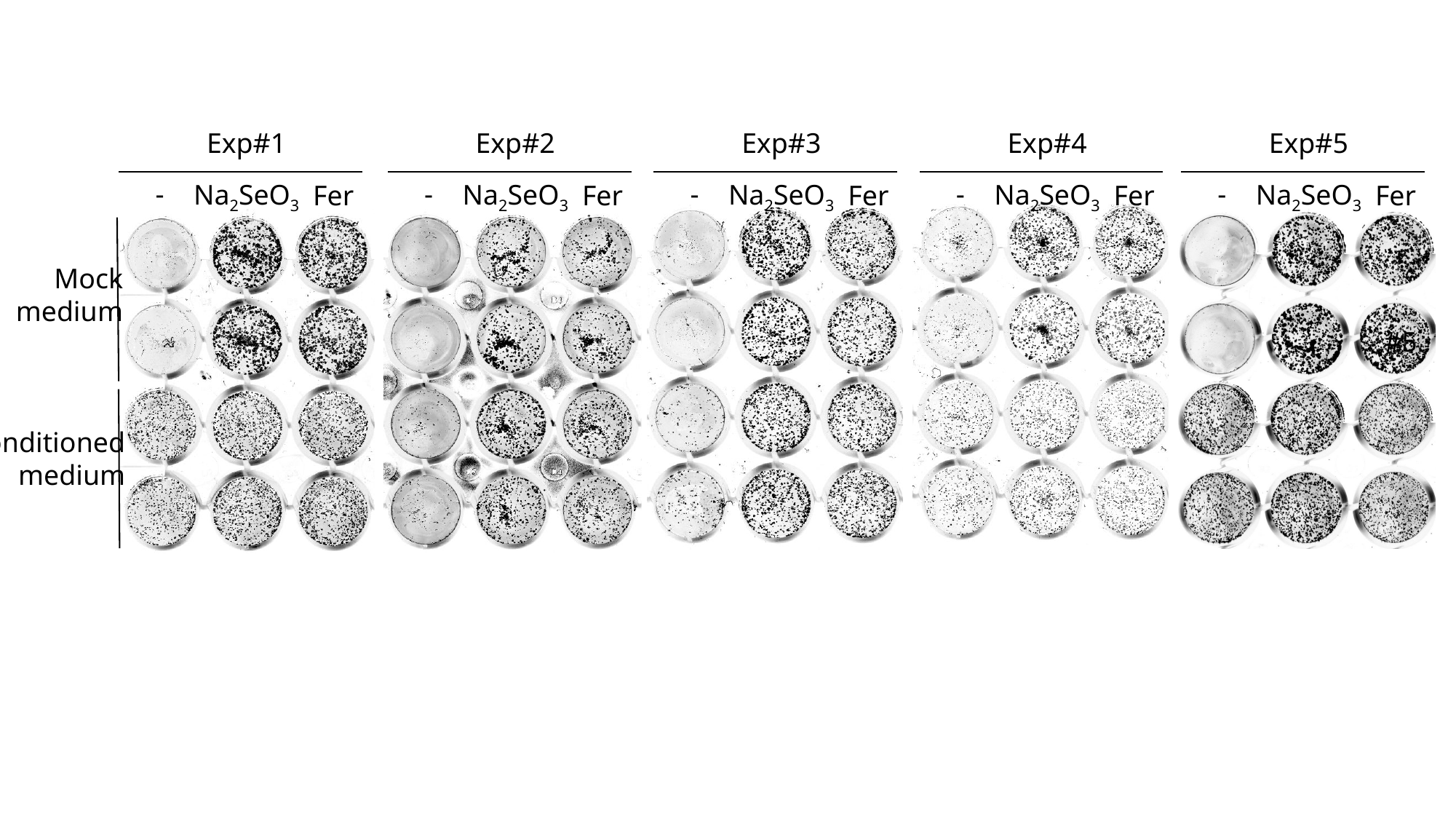

Exp#1
-
Na2SeO3
Fer
Exp#2
-
Na2SeO3
Fer
Exp#3
-
Na2SeO3
Fer
Exp#4
-
Na2SeO3
Fer
Exp#5
-
Na2SeO3
Fer
Mock
medium
#5
Conditioned
medium
