# Supplementary material for: Breast cancer secretes anti-ferroptotic MUFAs and depends on selenoprotein synthesis for metastasis
Source: EMBO Mol Med. 2024 Oct 21;16(11):7. doi: 10.1038/s44321-024-00142-x (PMC11555046; doi:10.1038/s44321-024-00142-x)
Supplement: Supplementary file 2 — Source data Fig. 1 [file 44321_2024_142_MOESM2_ESM.zip › Figure 1/D/pictures and labels.pptx]

## Slide 1
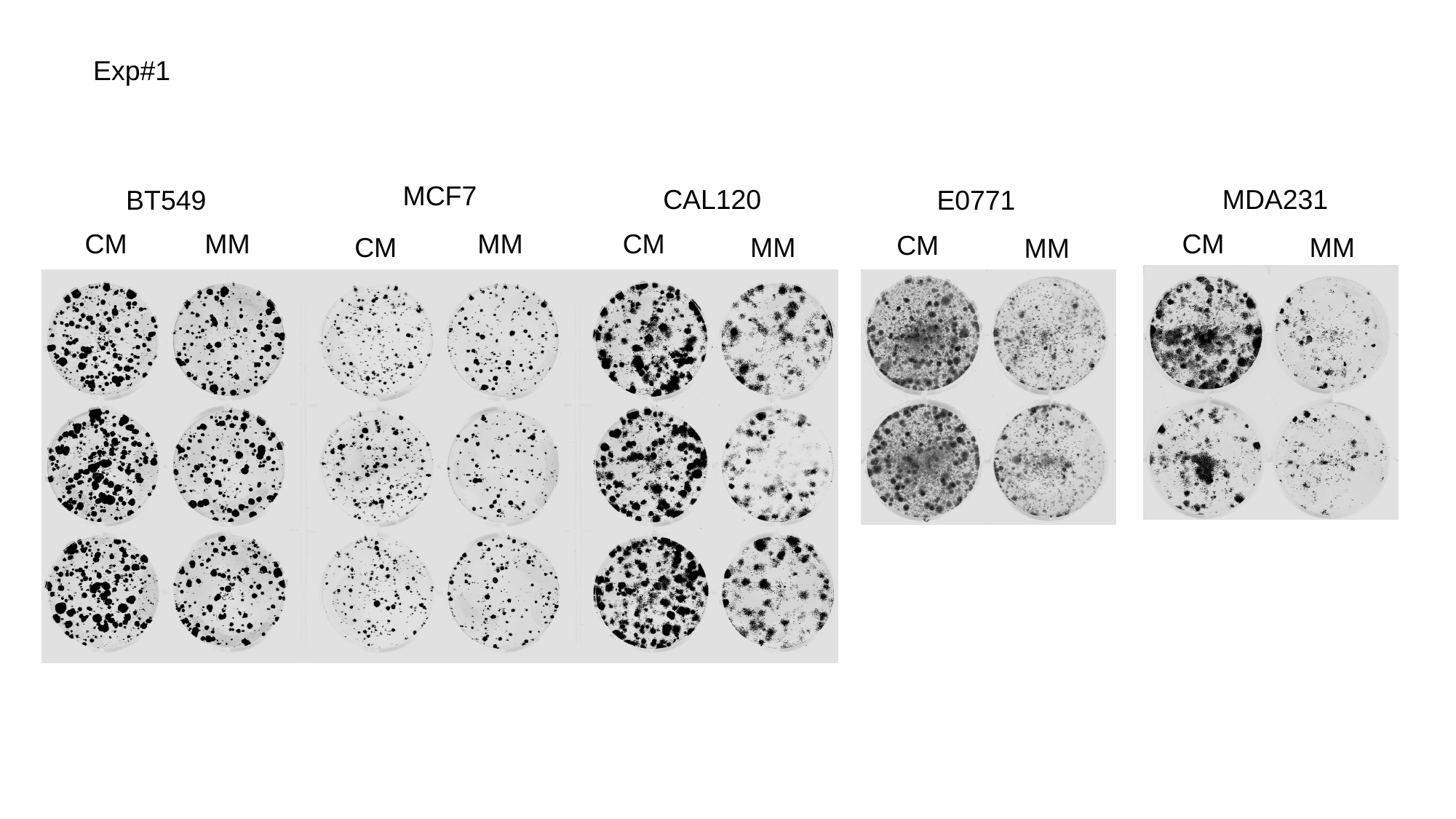

Exp#1
MCF7
CAL120
MDA231
BT549
E0771
CM
MM
MM
CM
CM
CM
CM
MM
MM
MM

## Slide 2
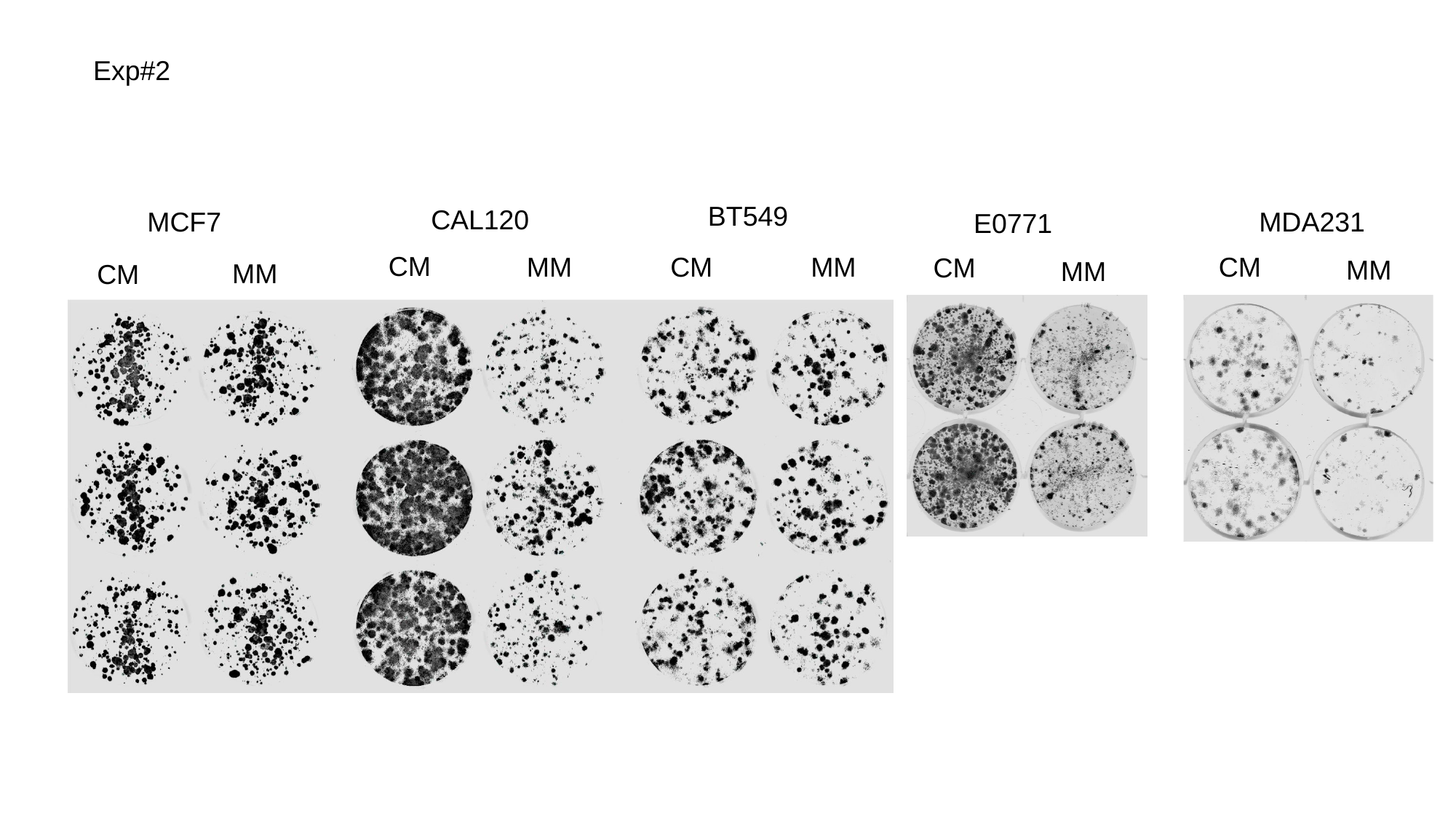

Exp#2
BT549
CAL120
MCF7
MDA231
E0771
CM
MM
CM
CM
MM
CM
MM
MM
MM
CM

## Slide 3
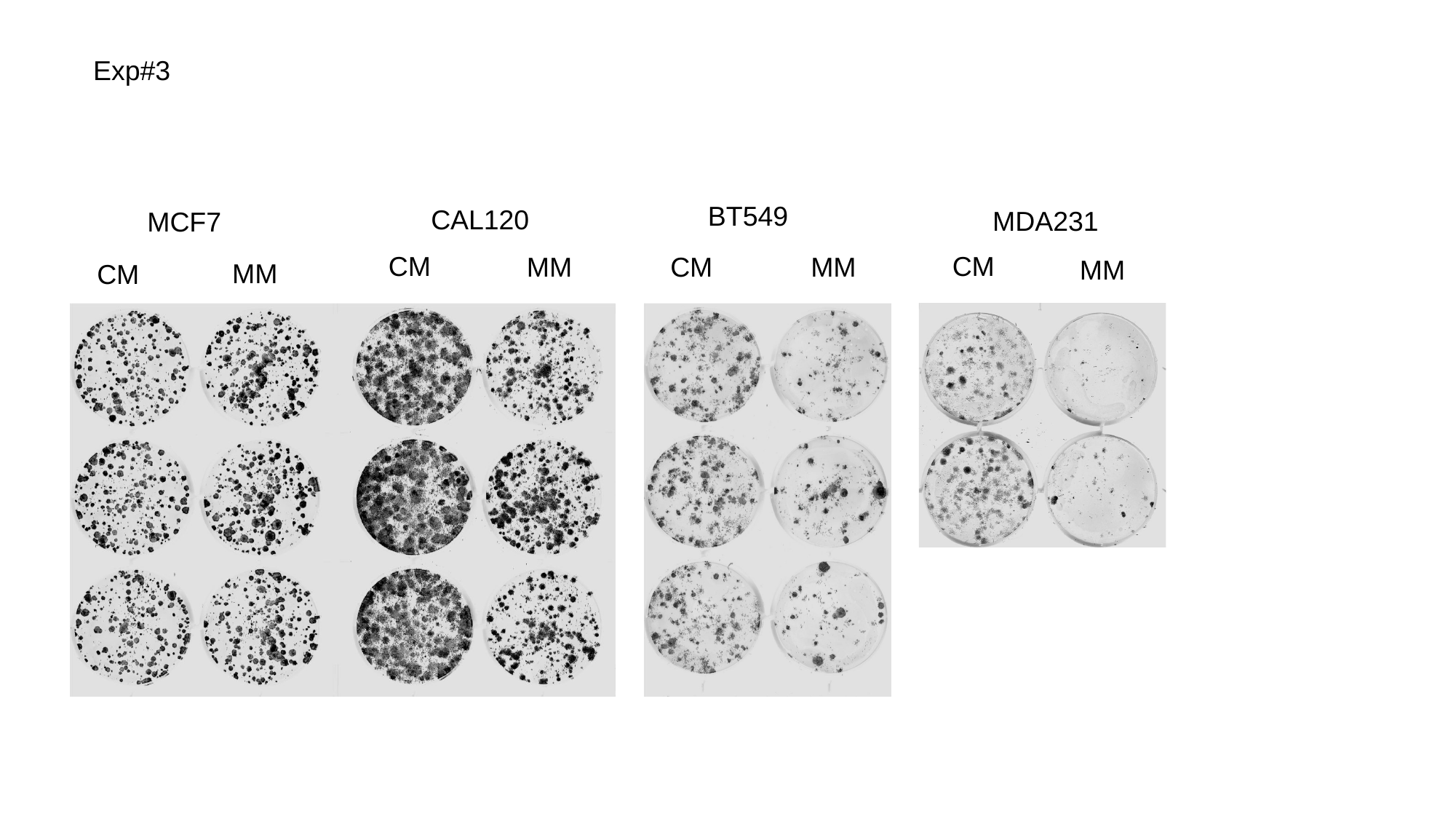

Exp#3
BT549
CAL120
MDA231
MCF7
CM
CM
MM
CM
MM
MM
MM
CM
