# Supplementary material for: Breast cancer secretes anti-ferroptotic MUFAs and depends on selenoprotein synthesis for metastasis
Source: EMBO Mol Med. 2024 Oct 21;16(11):7. doi: 10.1038/s44321-024-00142-x (PMC11555046; doi:10.1038/s44321-024-00142-x)
Supplement: Supplementary file 2 — Source data Fig. 1 [file 44321_2024_142_MOESM2_ESM.zip › Figure 1/E/pictures and labels.pptx]

## Slide 1
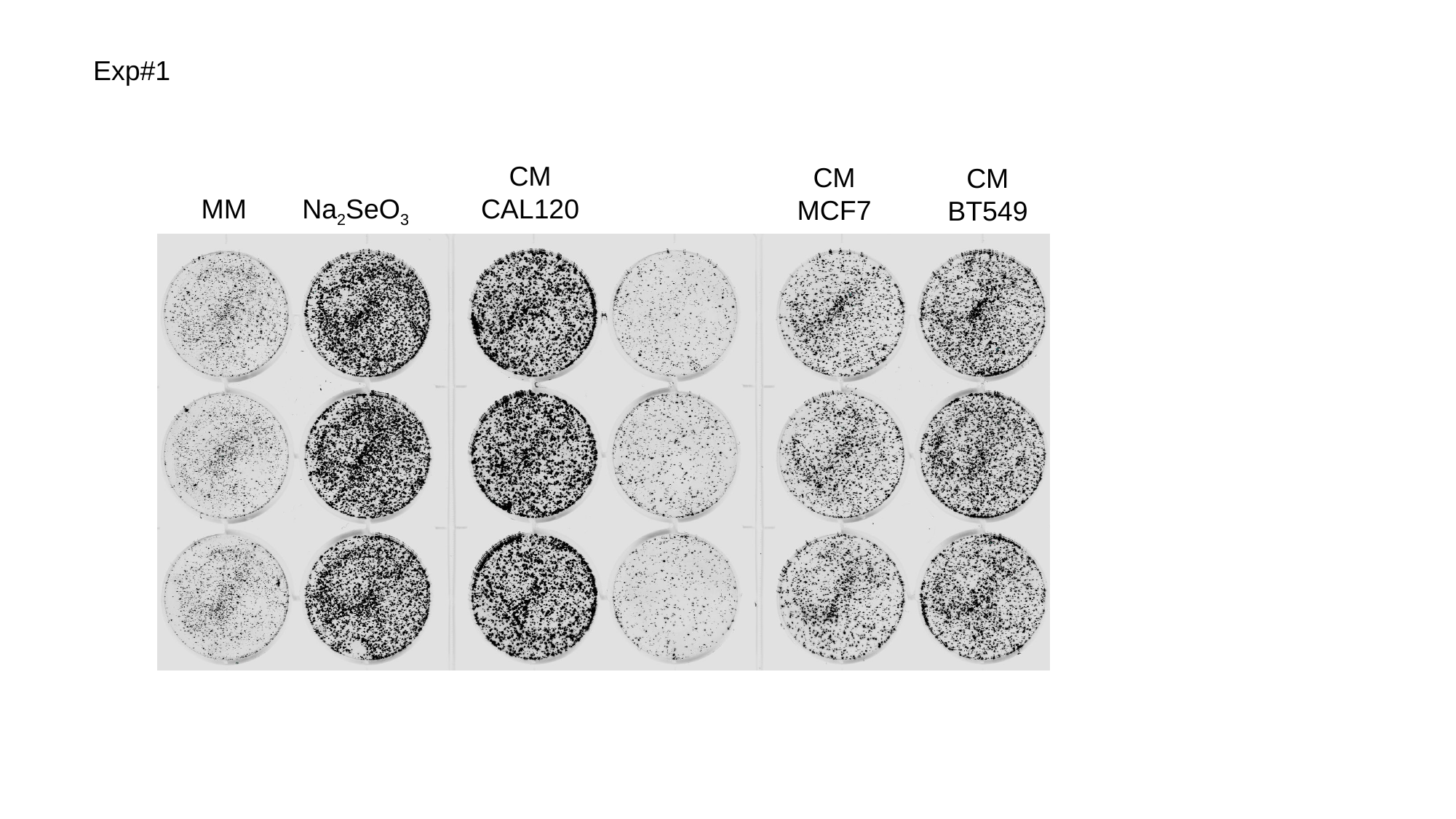

Exp#1
CM
CAL120
CM
MCF7
CM
BT549
MM
Na2SeO3

## Slide 2
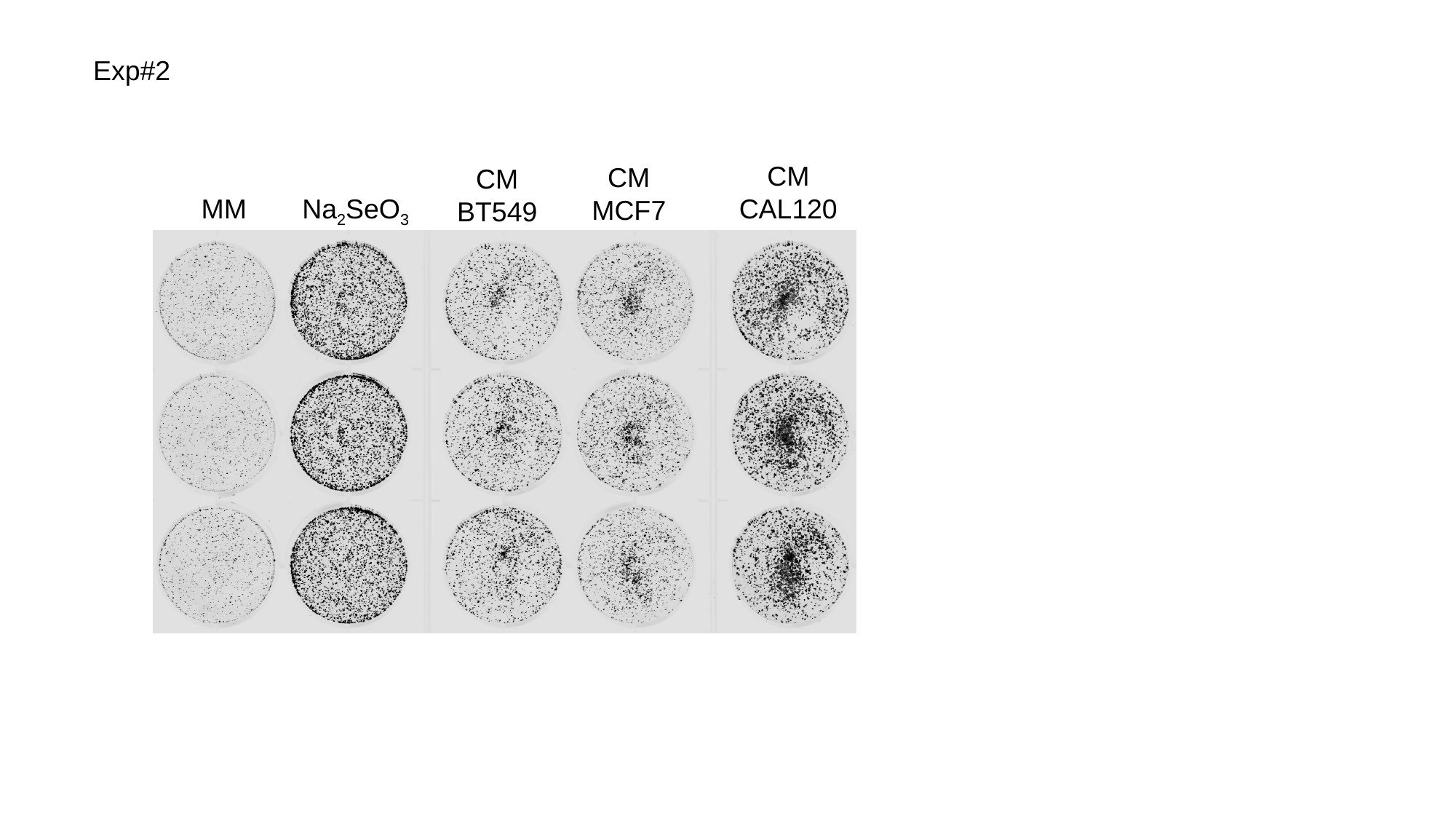

Exp#2
CM
CAL120
CM
MCF7
CM
BT549
MM
Na2SeO3

## Slide 3
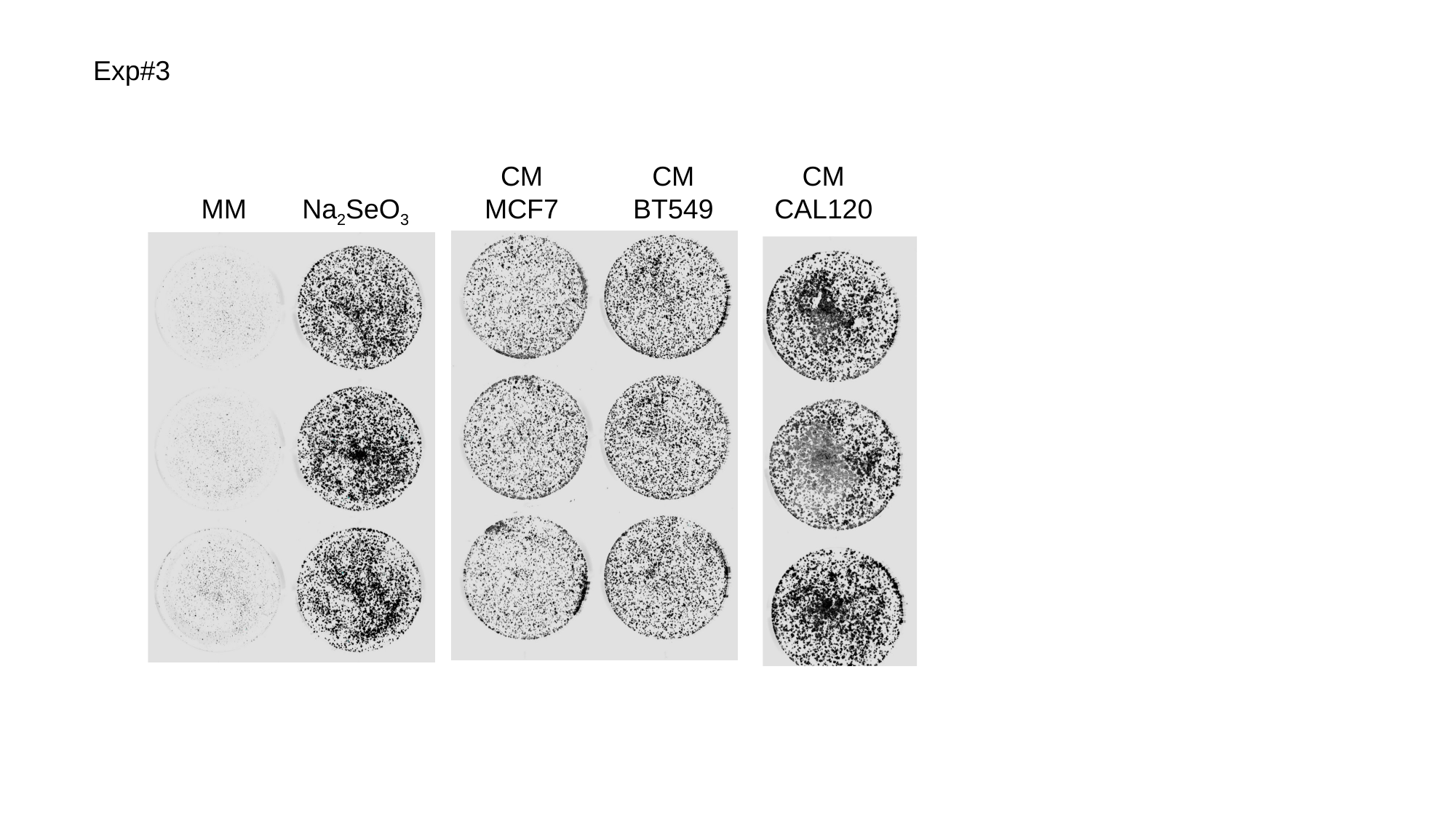

Exp#3
CM
BT549
CM
MCF7
CM
CAL120
MM
Na2SeO3
