# Supplementary figures and images for: Breast cancer secretes anti-ferroptotic MUFAs and depends on selenoprotein synthesis for metastasis (part 1 of 2)
Source: EMBO Mol Med. 2024 Oct 21;16(11):7. doi: 10.1038/s44321-024-00142-x (PMC11555046; doi:10.1038/s44321-024-00142-x)

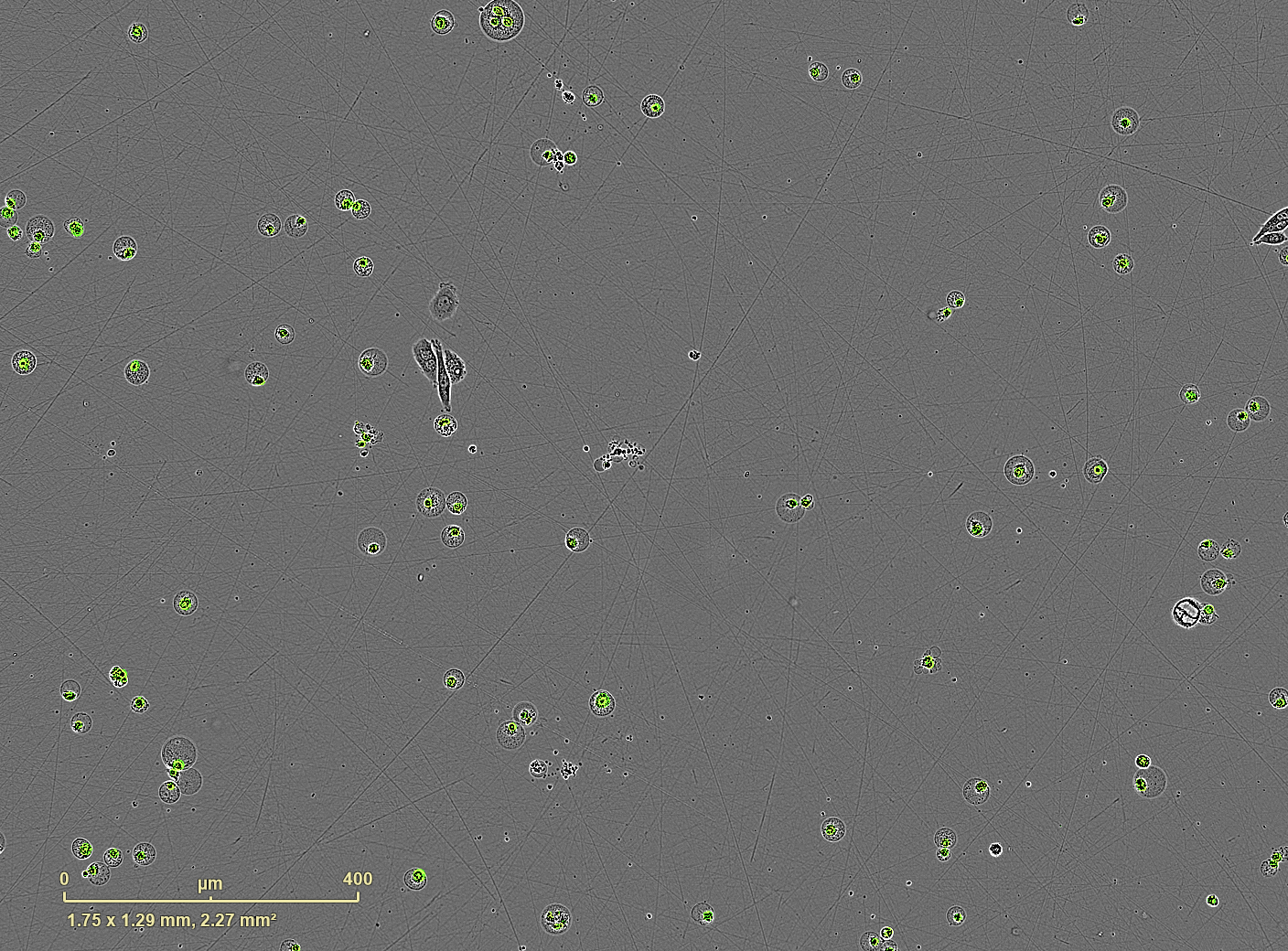

Supplement: Supplementary file 2 — Source data Fig. 1 [file 44321_2024_142_MOESM2_ESM.zip › Figure 1/A/Vehicle.tif]

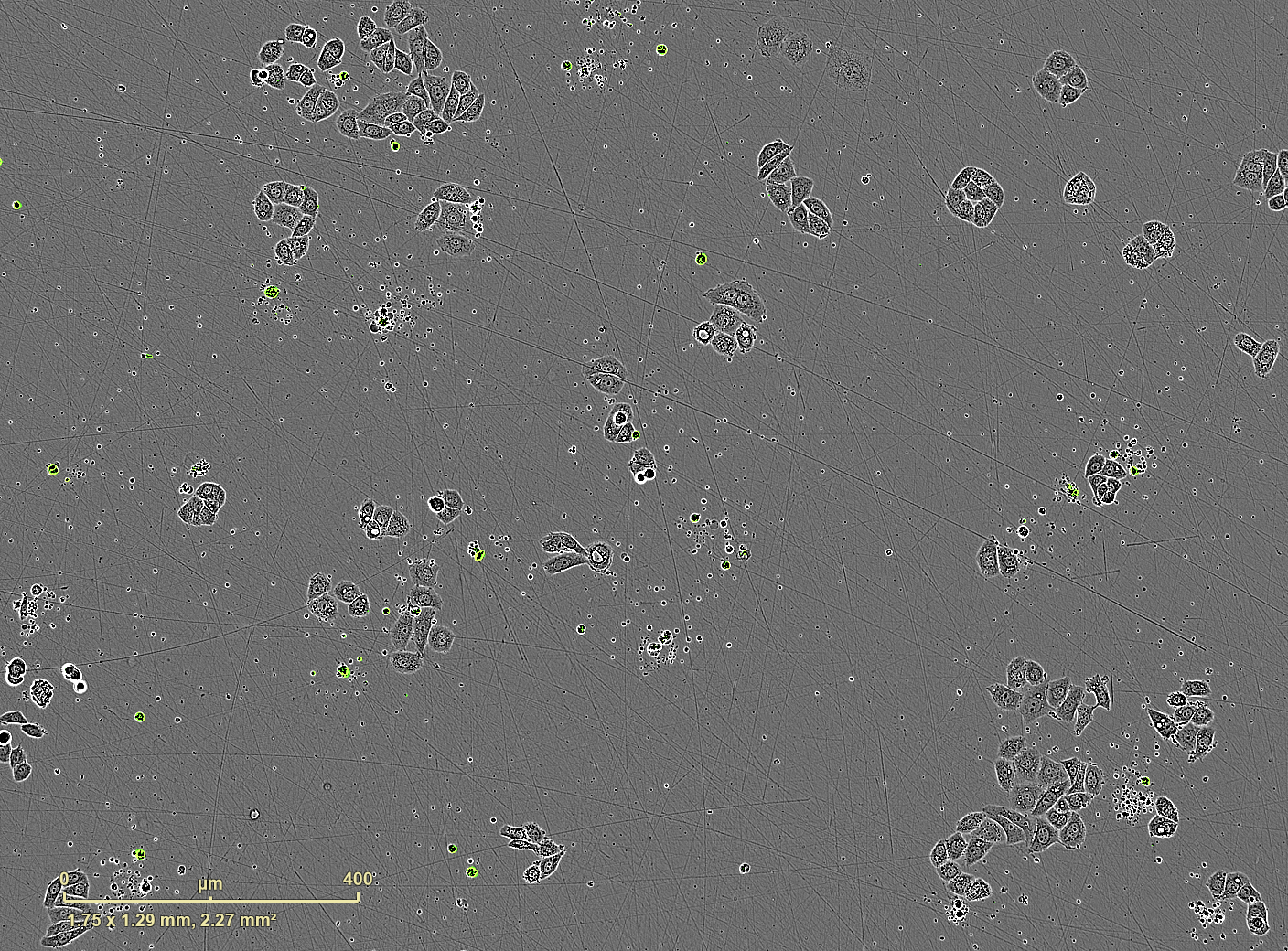

Supplement: Supplementary file 2 — Source data Fig. 1 [file 44321_2024_142_MOESM2_ESM.zip › Figure 1/A/Ferrostatin.tif]

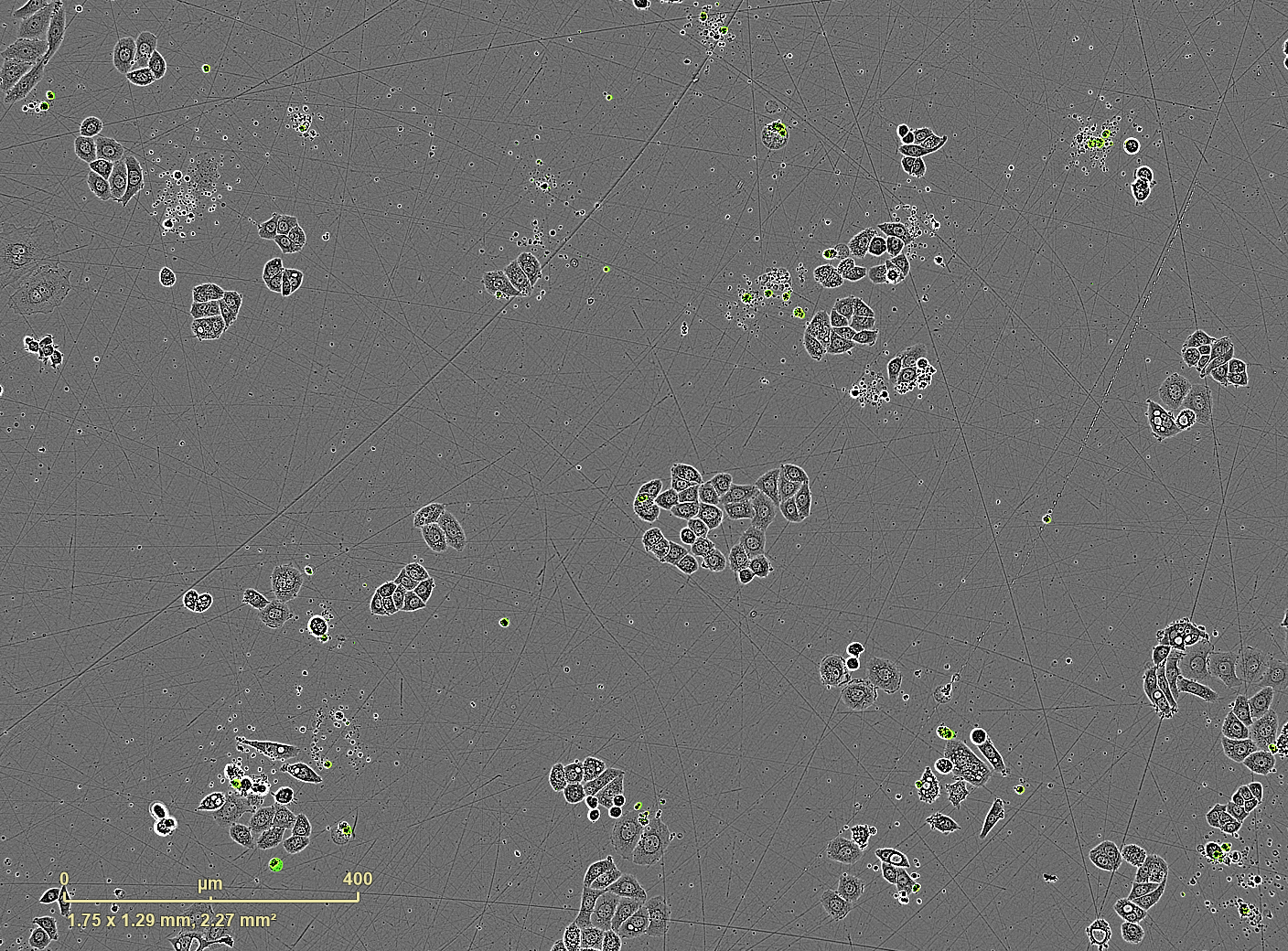

Supplement: Supplementary file 2 — Source data Fig. 1 [file 44321_2024_142_MOESM2_ESM.zip › Figure 1/A/Na2SeO3.tif]

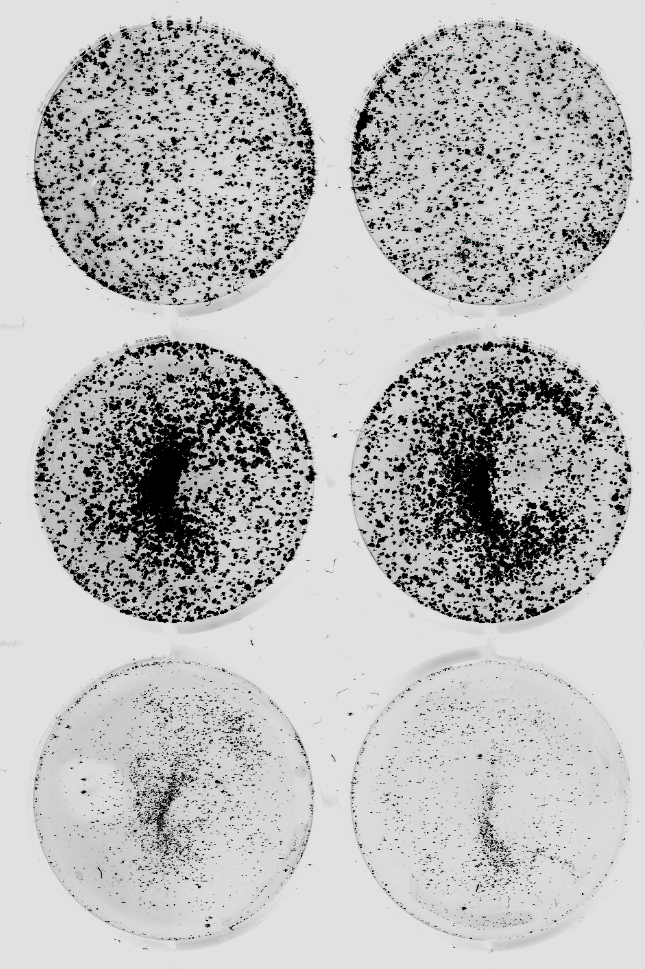

Supplement: Supplementary file 2 — Source data Fig. 1 [file 44321_2024_142_MOESM2_ESM.zip › Figure 1/F/exp#3 MM Na2SeO3 CAF.tif]

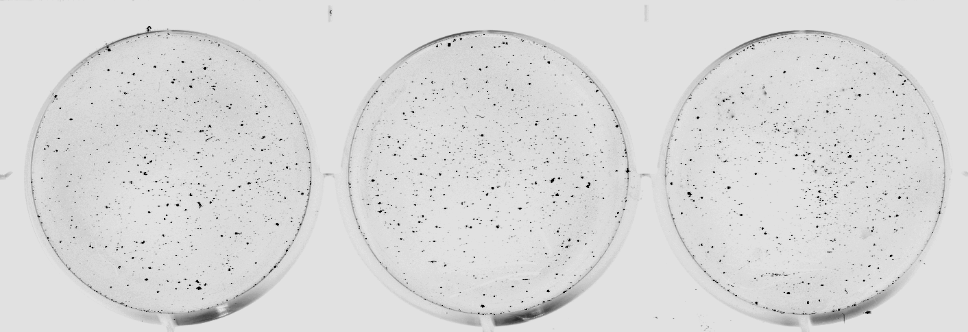

Supplement: Supplementary file 2 — Source data Fig. 1 [file 44321_2024_142_MOESM2_ESM.zip › Figure 1/F/exp#1 MF.tif]

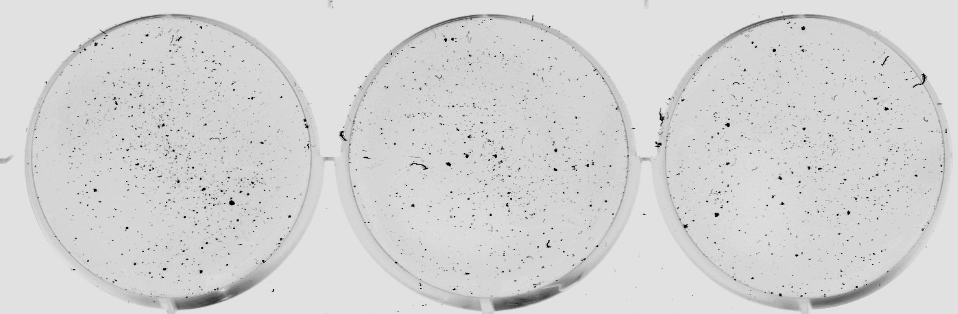

Supplement: Supplementary file 2 — Source data Fig. 1 [file 44321_2024_142_MOESM2_ESM.zip › Figure 1/F/exp#3 MF.tif]

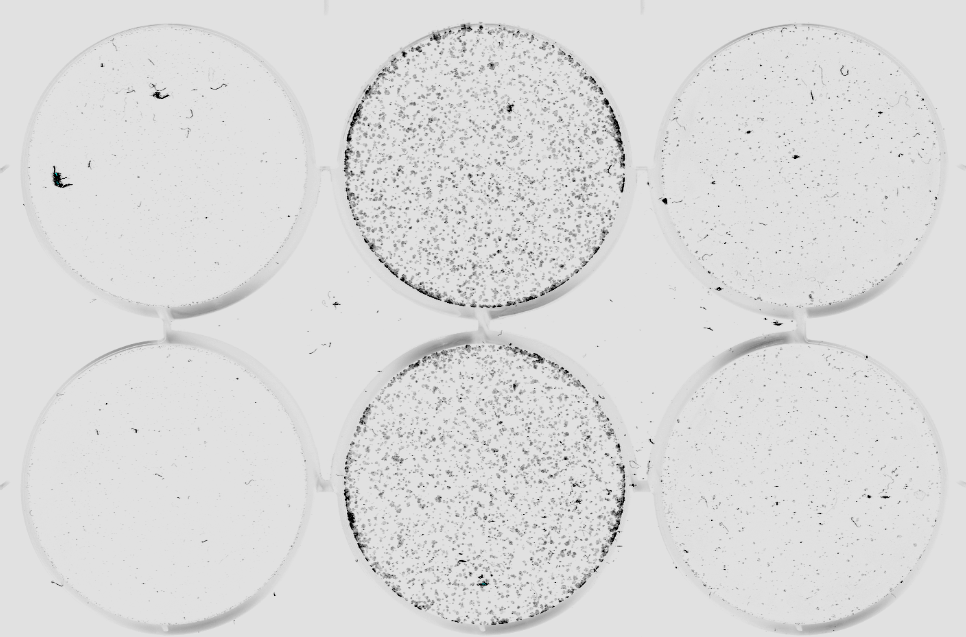

Supplement: Supplementary file 2 — Source data Fig. 1 [file 44321_2024_142_MOESM2_ESM.zip › Figure 1/F/exp#5.tif]

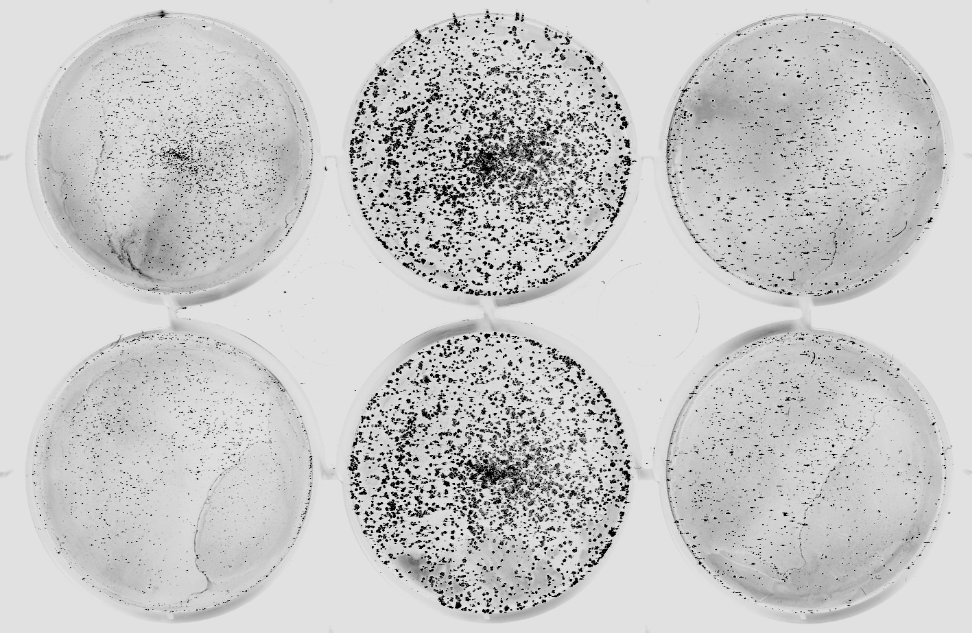

Supplement: Supplementary file 2 — Source data Fig. 1 [file 44321_2024_142_MOESM2_ESM.zip › Figure 1/F/exp#4.tif]

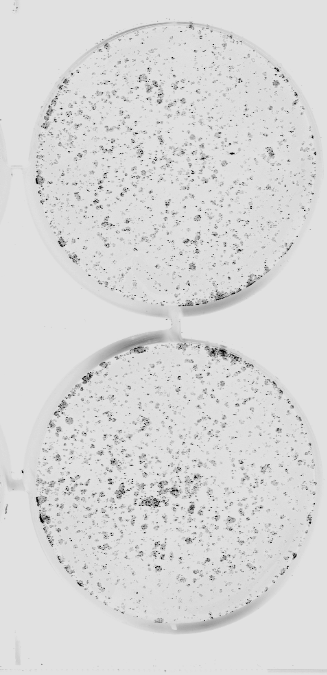

Supplement: Supplementary file 2 — Source data Fig. 1 [file 44321_2024_142_MOESM2_ESM.zip › Figure 1/F/exp#3 DF.tif]

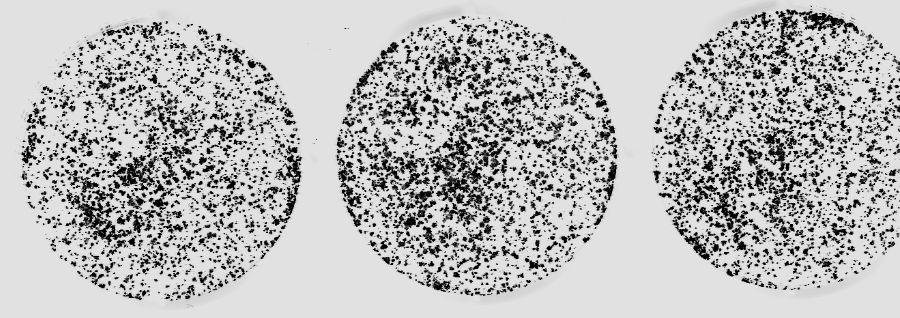

Supplement: Supplementary file 2 — Source data Fig. 1 [file 44321_2024_142_MOESM2_ESM.zip › Figure 1/F/exp#1 CAF.tif]

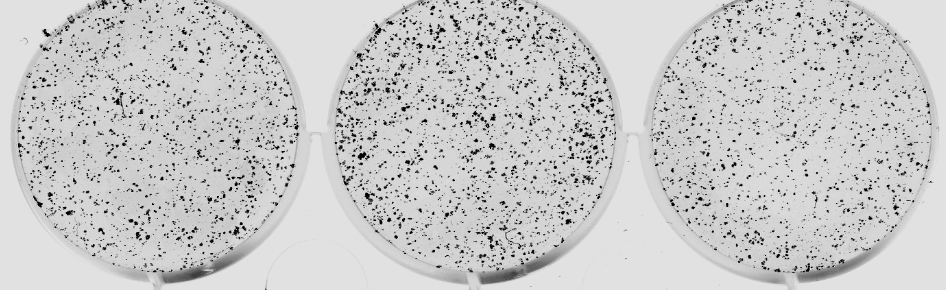

Supplement: Supplementary file 2 — Source data Fig. 1 [file 44321_2024_142_MOESM2_ESM.zip › Figure 1/F/exp#2 MF.tif]

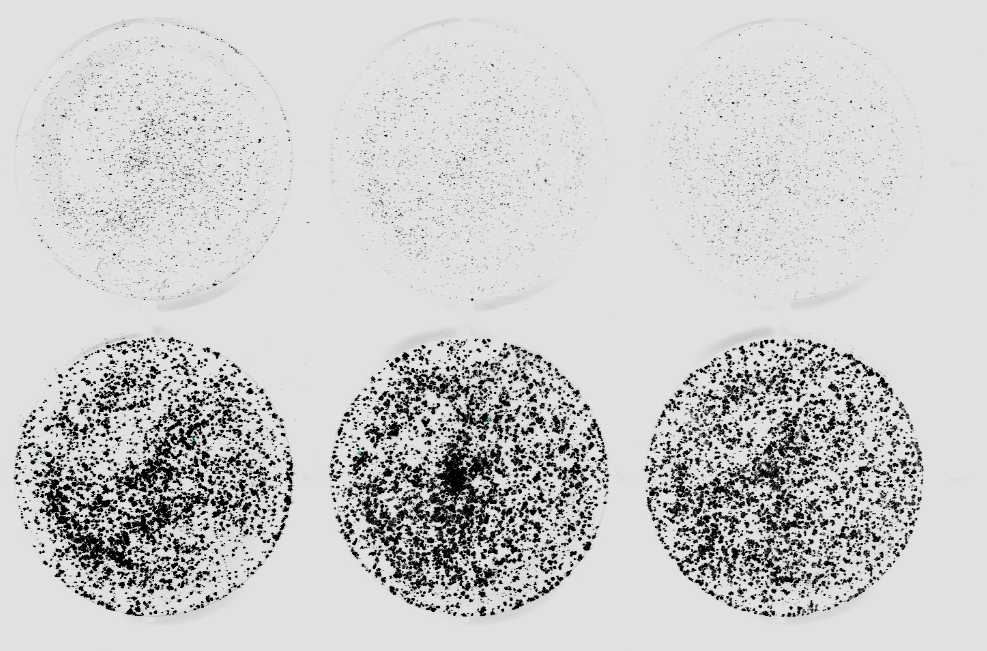

Supplement: Supplementary file 2 — Source data Fig. 1 [file 44321_2024_142_MOESM2_ESM.zip › Figure 1/F/exp#1 MM Na2SeO3.tif]

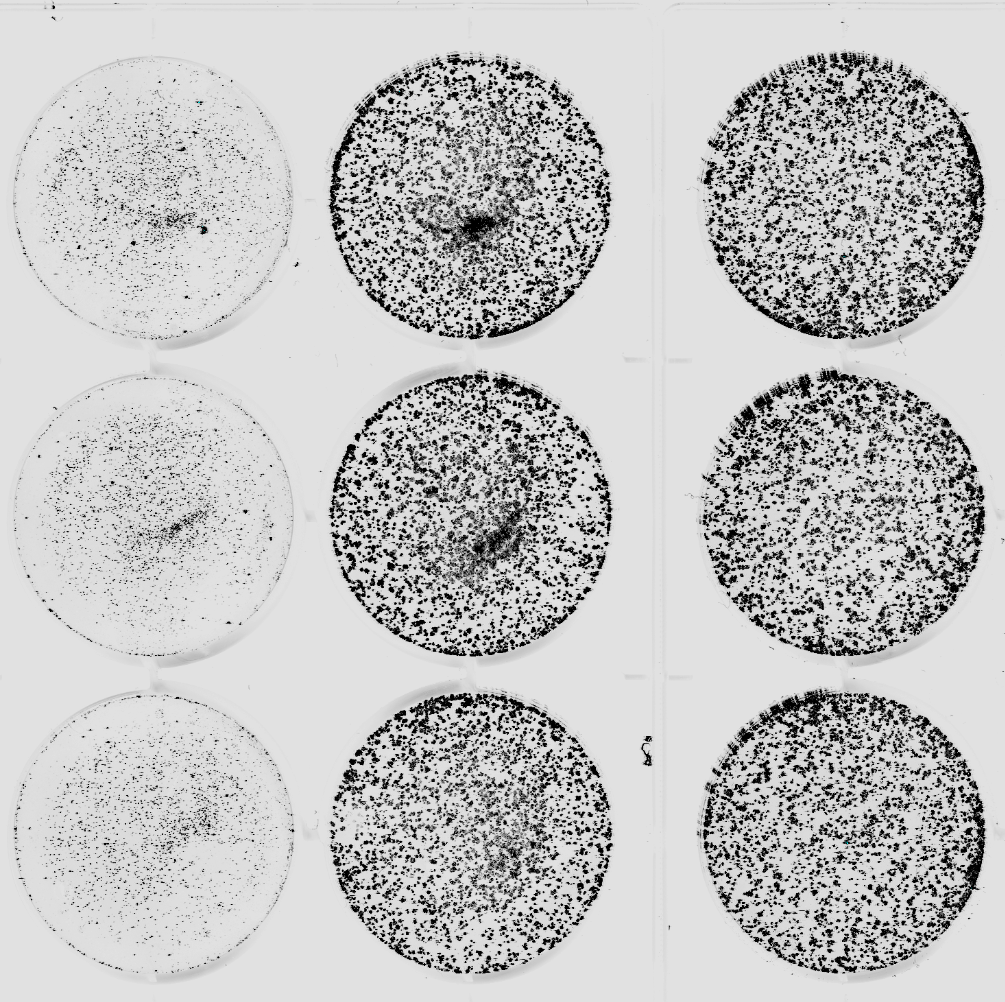

Supplement: Supplementary file 2 — Source data Fig. 1 [file 44321_2024_142_MOESM2_ESM.zip › Figure 1/F/exp#2 MM Na2SeO3 CAF.tif]

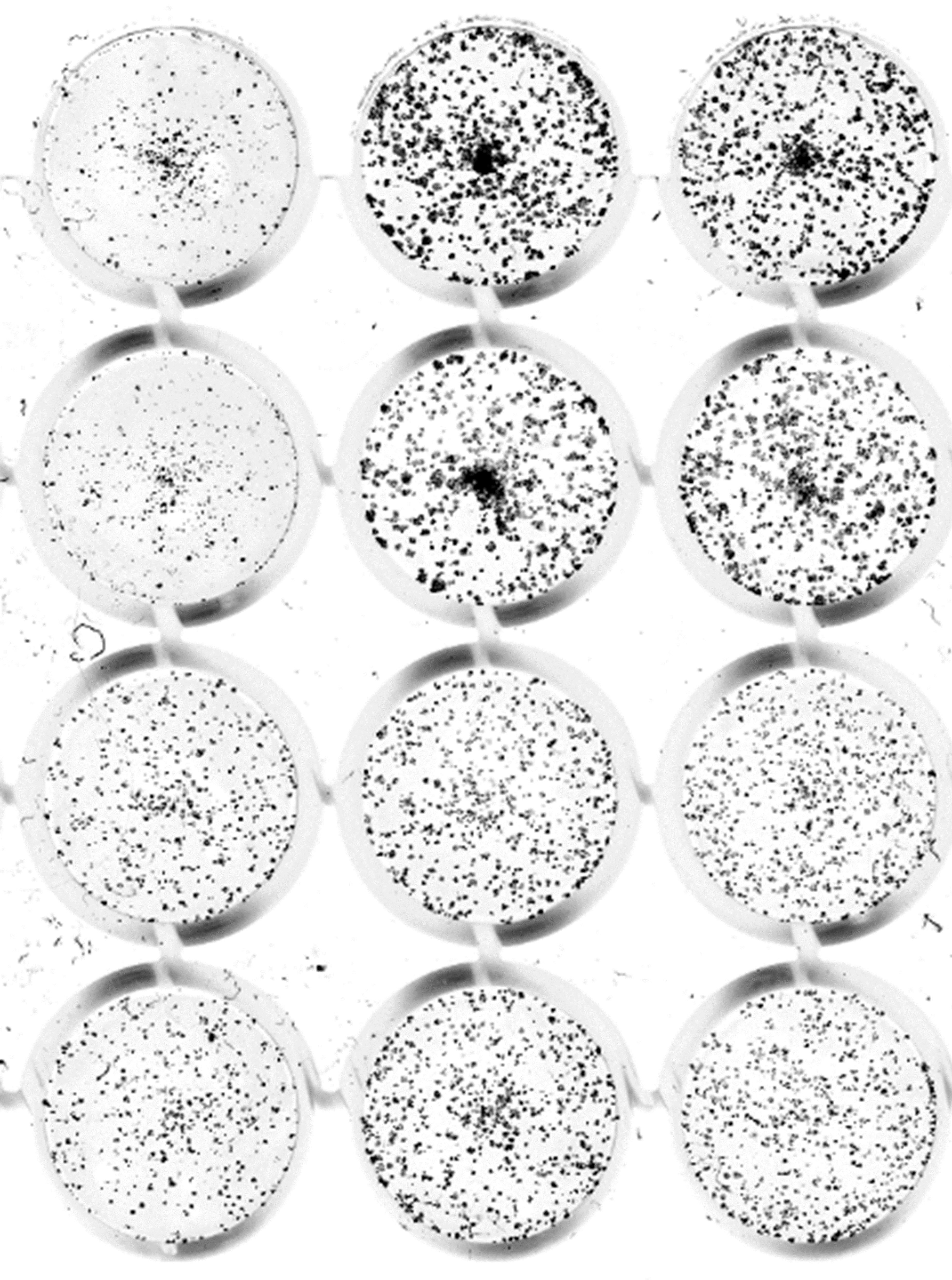

Supplement: Supplementary file 2 — Source data Fig. 1 [file 44321_2024_142_MOESM2_ESM.zip › Figure 1/C/exp4.tif]

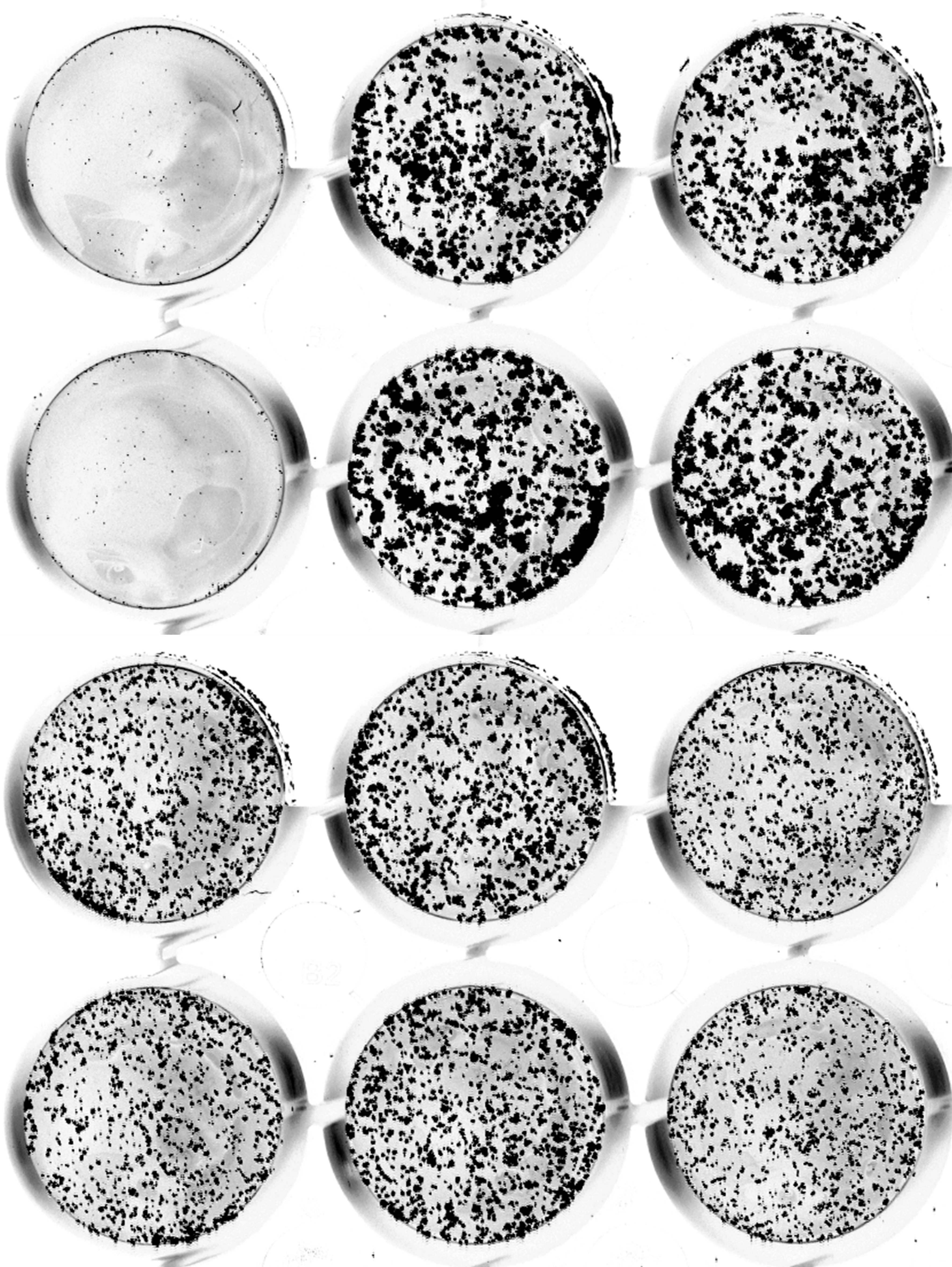

Supplement: Supplementary file 2 — Source data Fig. 1 [file 44321_2024_142_MOESM2_ESM.zip › Figure 1/C/exp5.tif]

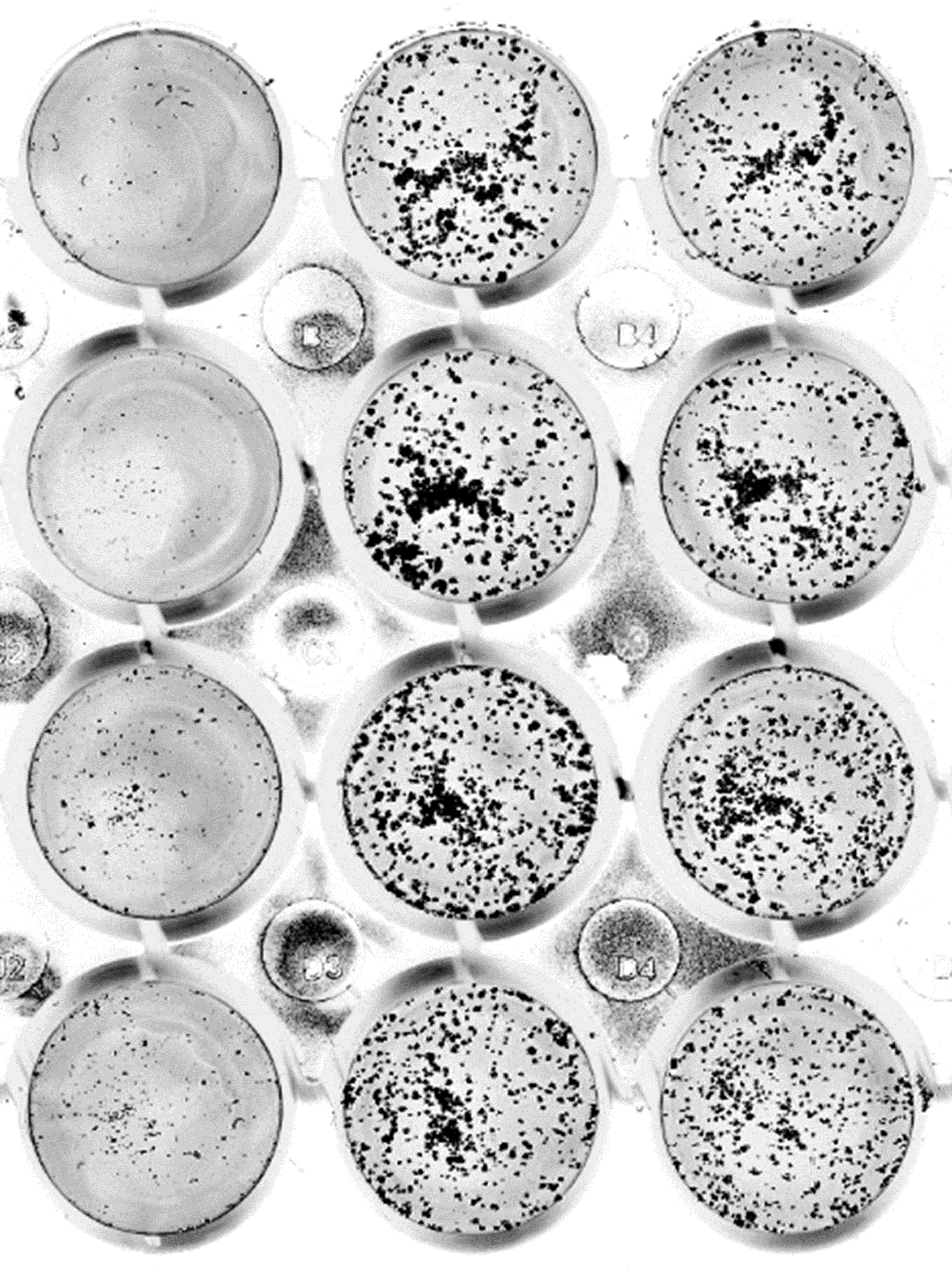

Supplement: Supplementary file 2 — Source data Fig. 1 [file 44321_2024_142_MOESM2_ESM.zip › Figure 1/C/exp2.tif]

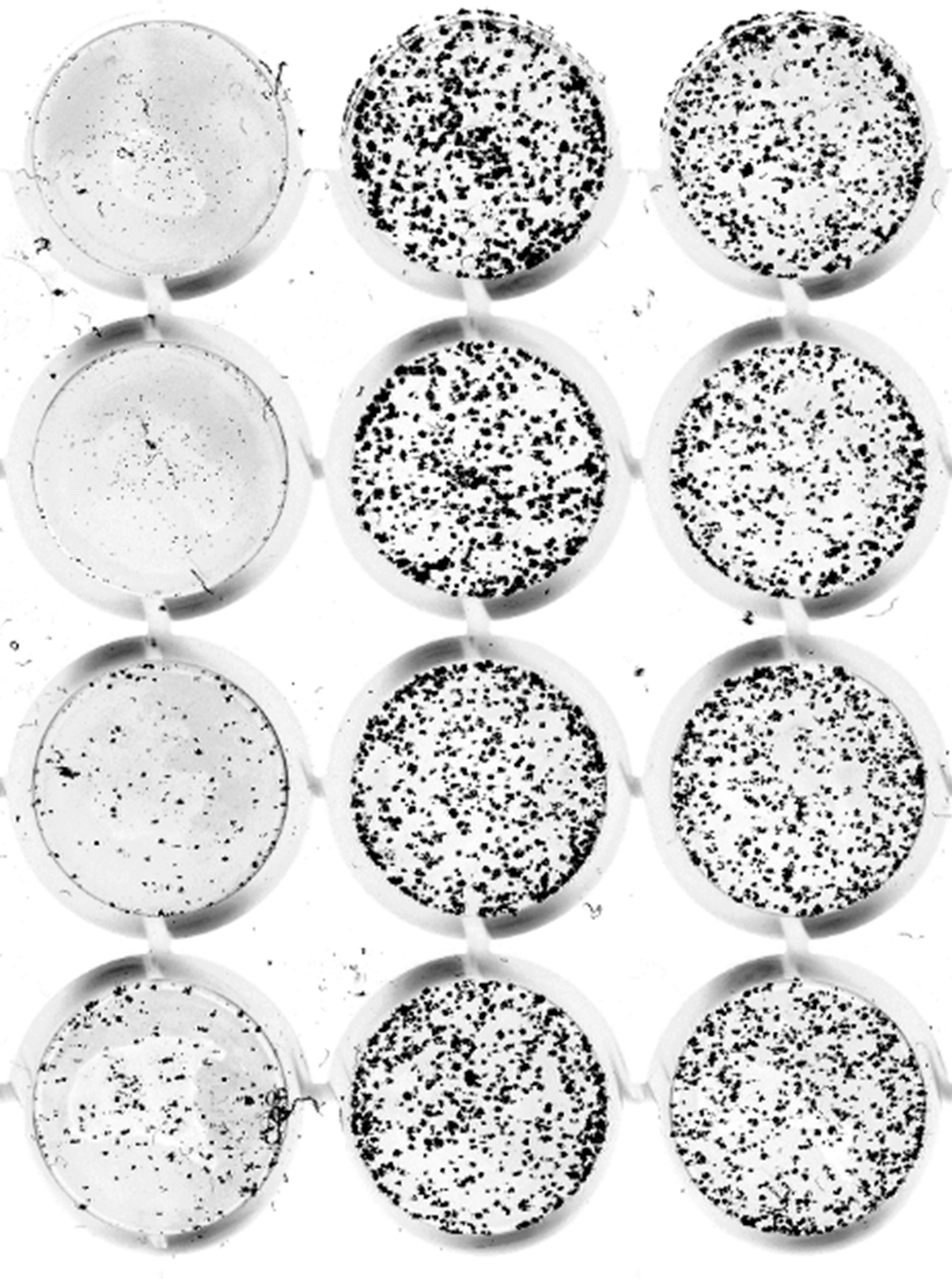

Supplement: Supplementary file 2 — Source data Fig. 1 [file 44321_2024_142_MOESM2_ESM.zip › Figure 1/C/exp3.tif]

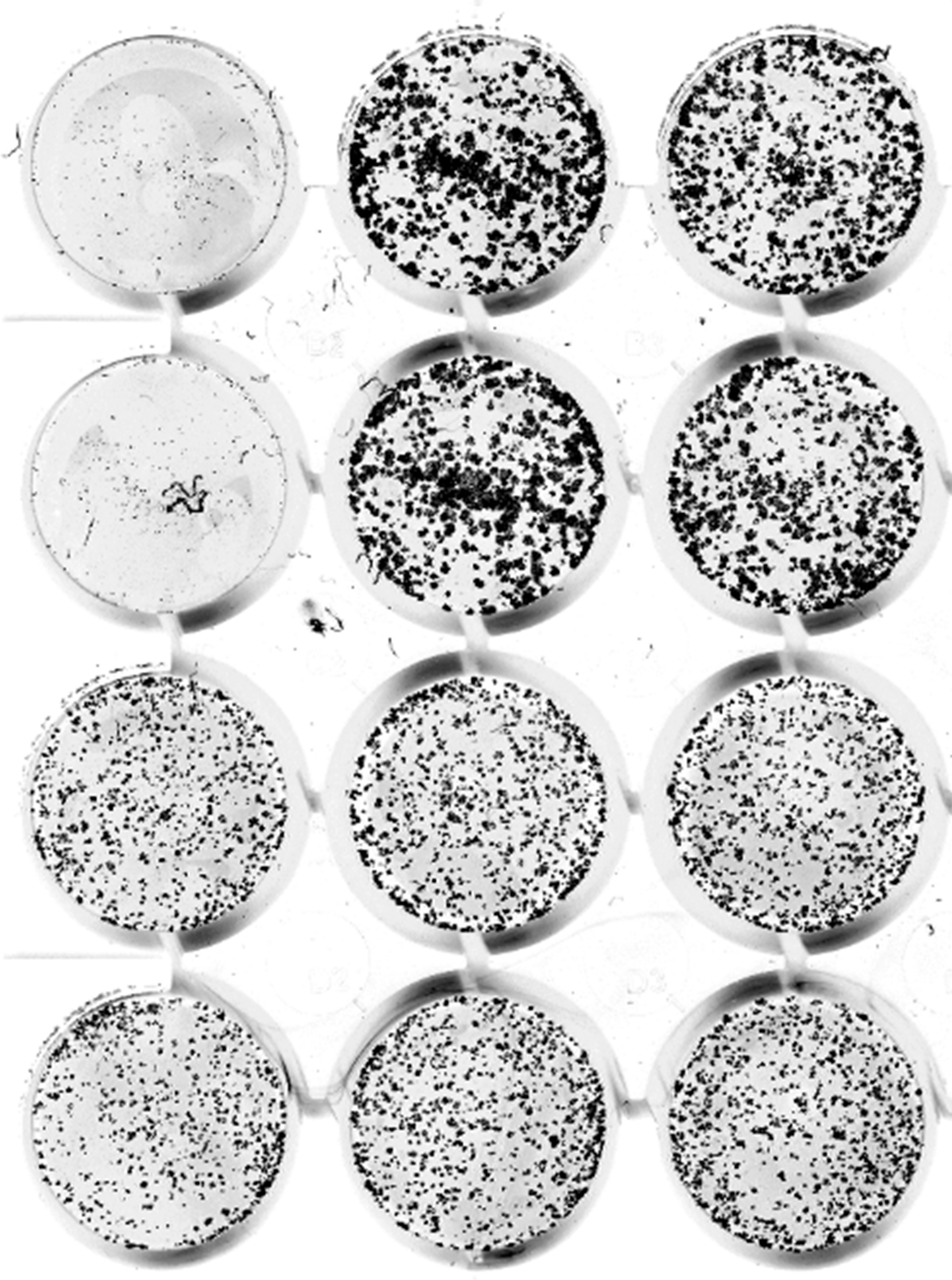

Supplement: Supplementary file 2 — Source data Fig. 1 [file 44321_2024_142_MOESM2_ESM.zip › Figure 1/C/exp1.tif]

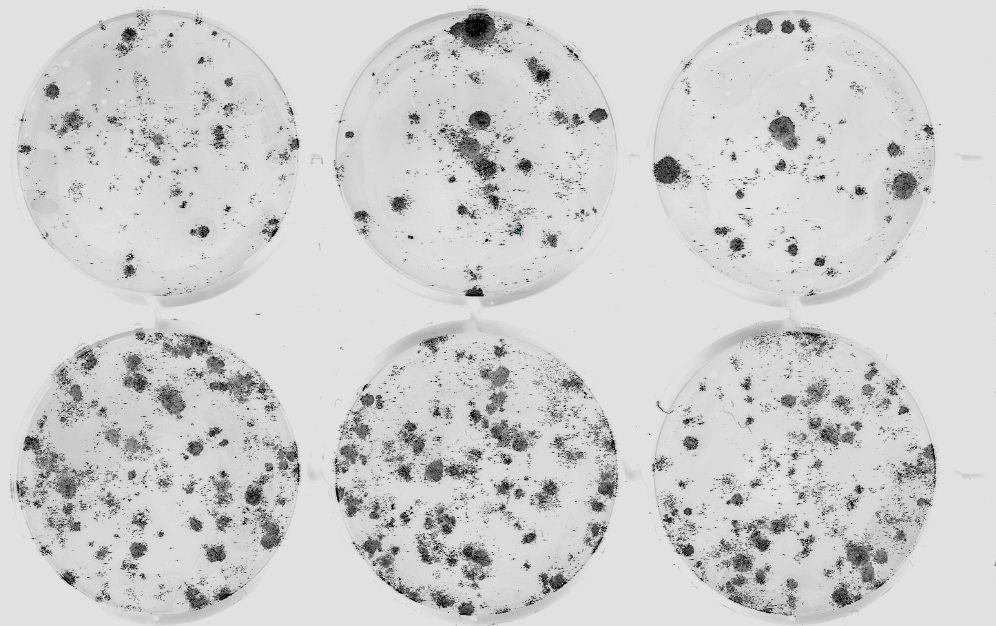

Supplement: Supplementary file 2 — Source data Fig. 1 [file 44321_2024_142_MOESM2_ESM.zip › Figure 1/D/exp#3 bt549.tif]

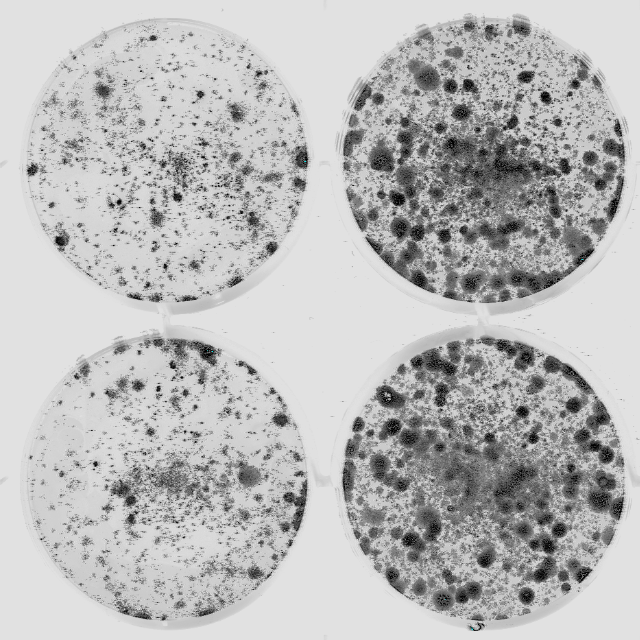

Supplement: Supplementary file 2 — Source data Fig. 1 [file 44321_2024_142_MOESM2_ESM.zip › Figure 1/D/exp#1 E0771.tif]

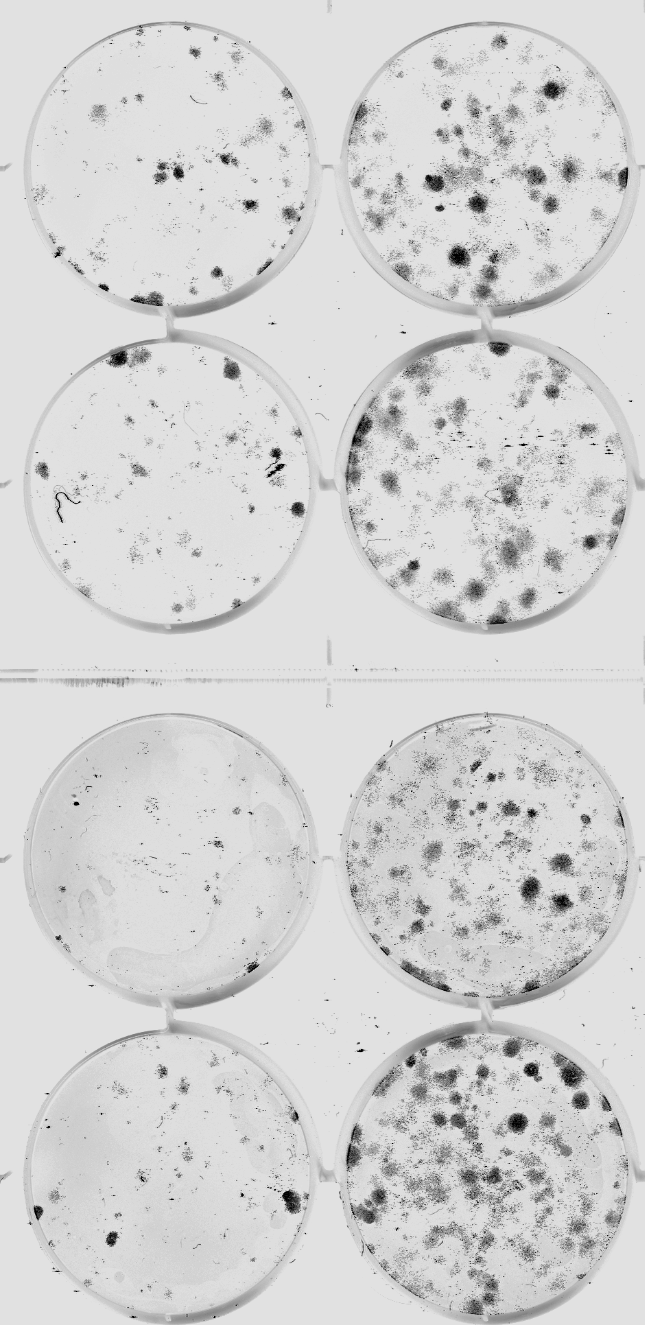

Supplement: Supplementary file 2 — Source data Fig. 1 [file 44321_2024_142_MOESM2_ESM.zip › Figure 1/D/exp#2 +exp#3 mda231.tif]

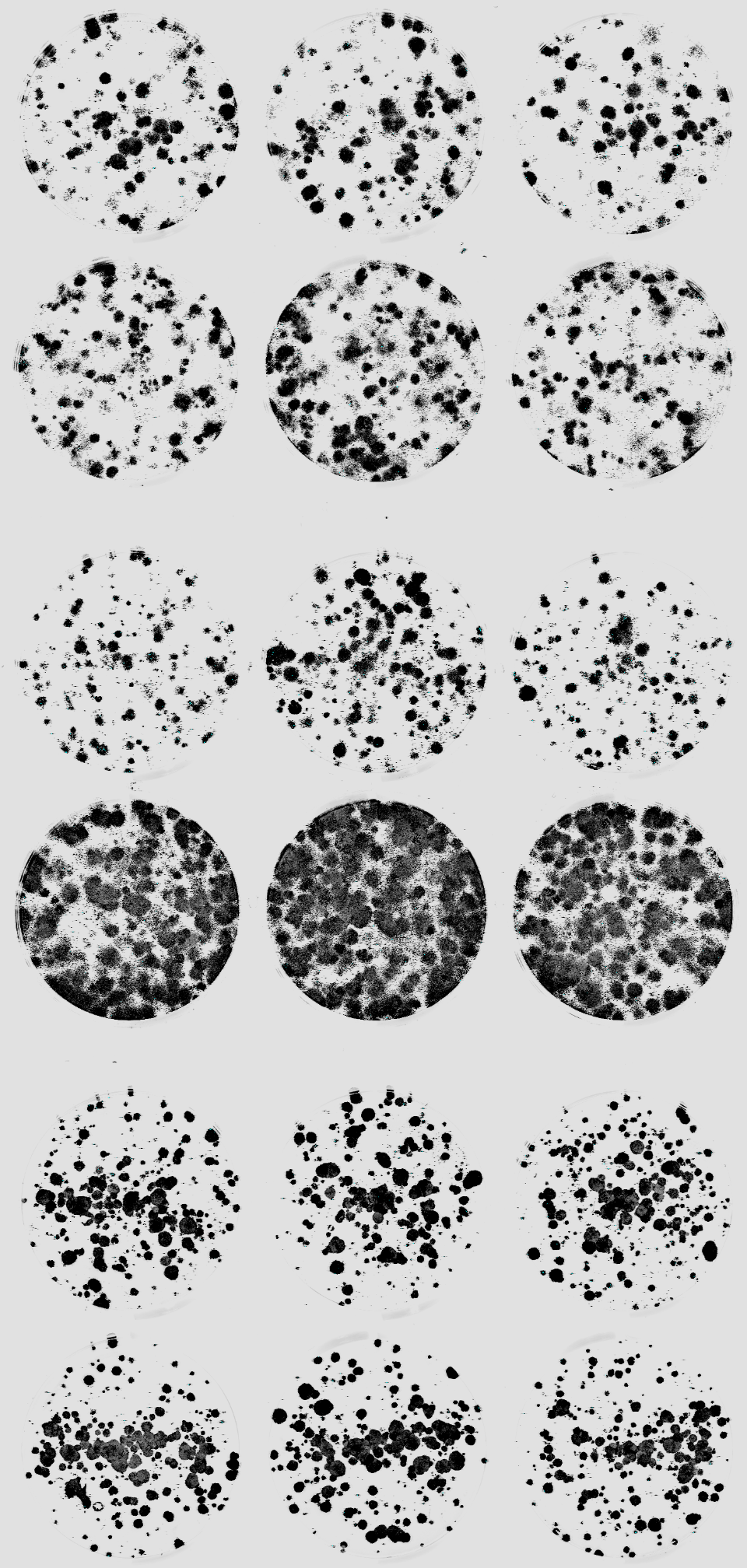

Supplement: Supplementary file 2 — Source data Fig. 1 [file 44321_2024_142_MOESM2_ESM.zip › Figure 1/D/exp#2 bt549 cal120 mcf7.tif]

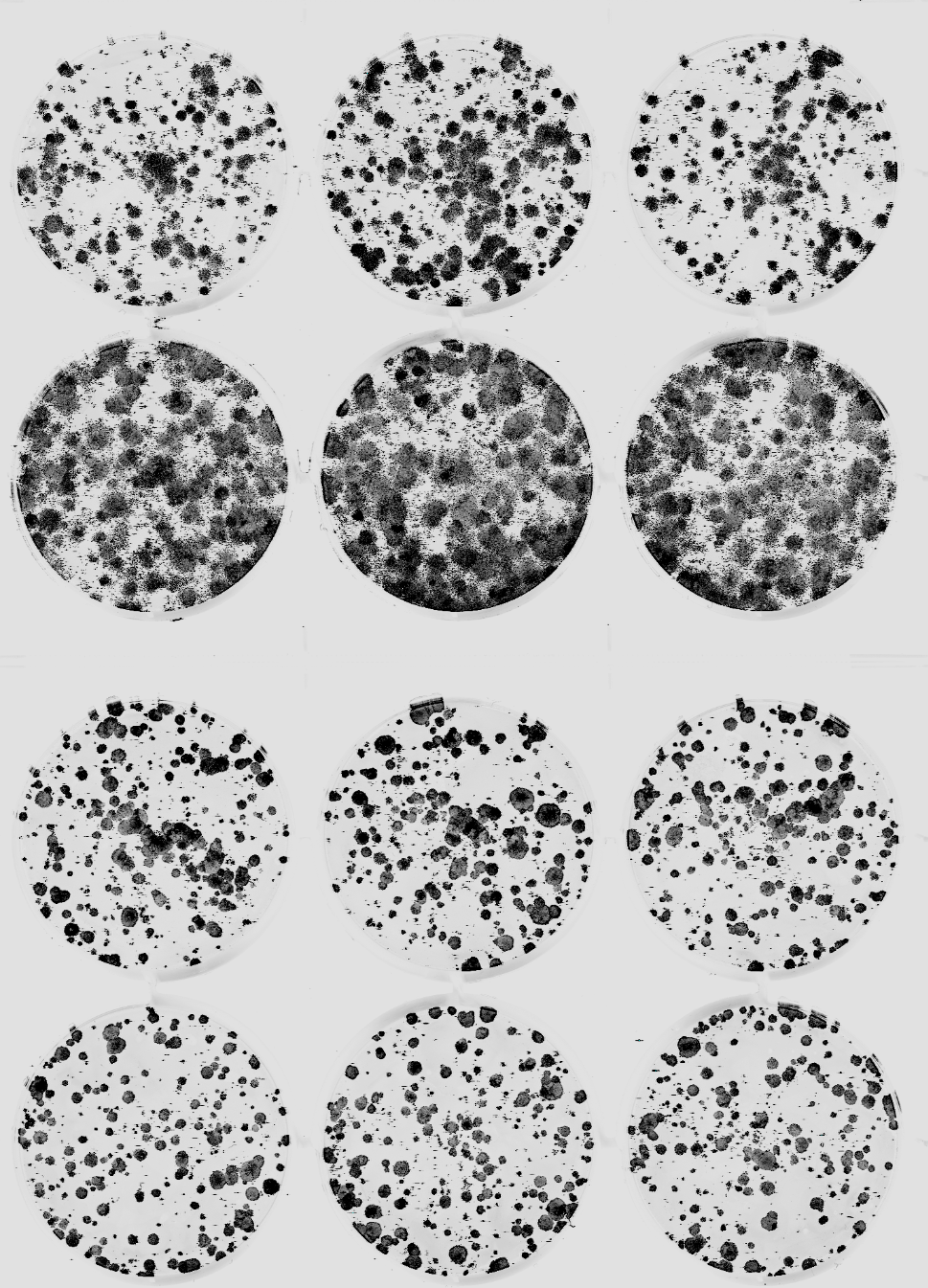

Supplement: Supplementary file 2 — Source data Fig. 1 [file 44321_2024_142_MOESM2_ESM.zip › Figure 1/D/exp#3 cal120 mcf7.tif]

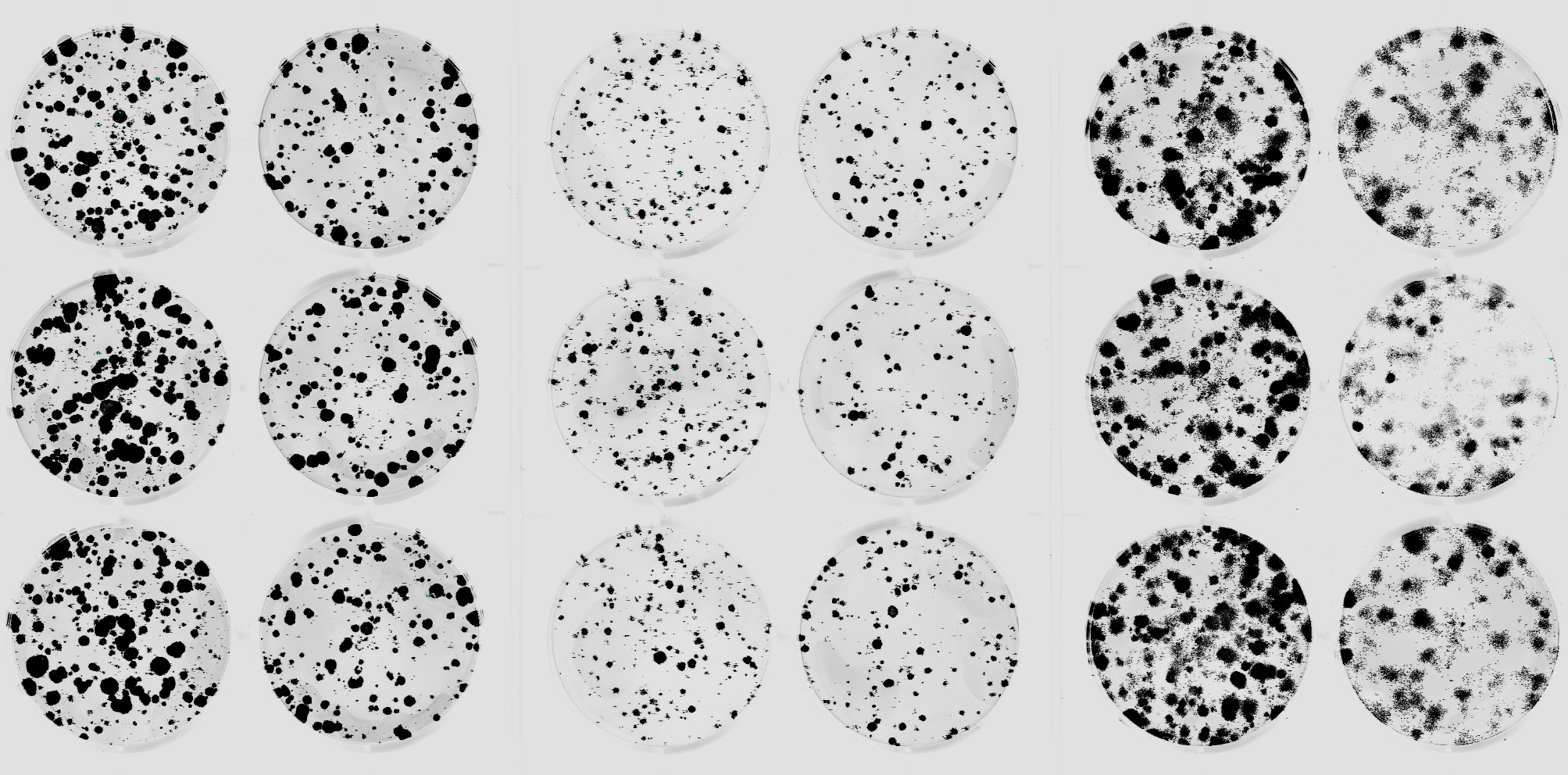

Supplement: Supplementary file 2 — Source data Fig. 1 [file 44321_2024_142_MOESM2_ESM.zip › Figure 1/D/exp#1 bt549 cal120 mcf7.tif]

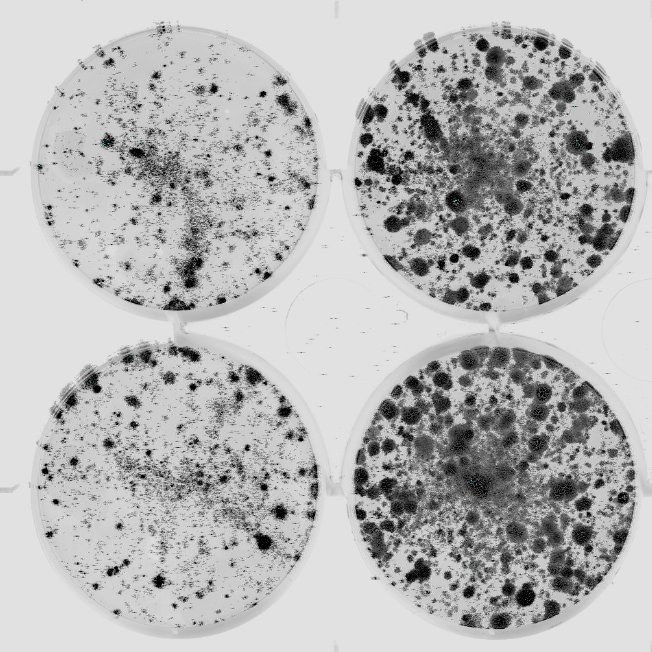

Supplement: Supplementary file 2 — Source data Fig. 1 [file 44321_2024_142_MOESM2_ESM.zip › Figure 1/D/exp#2 E0771.tif]

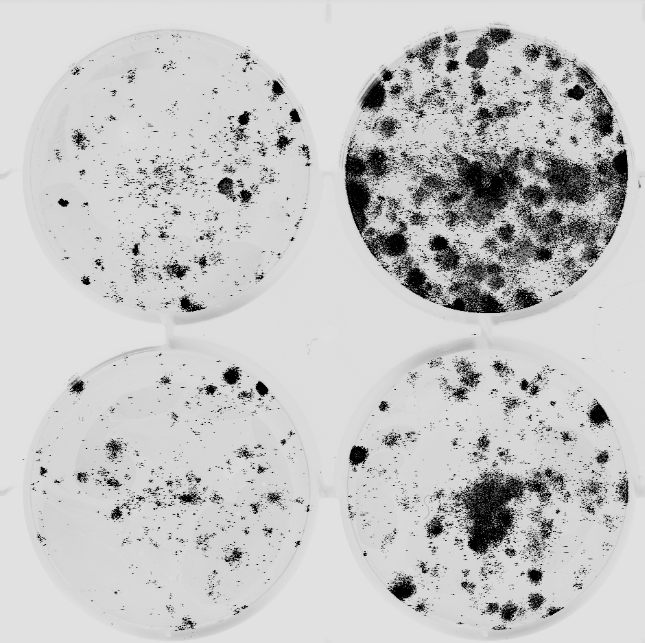

Supplement: Supplementary file 2 — Source data Fig. 1 [file 44321_2024_142_MOESM2_ESM.zip › Figure 1/D/exp#1 mda231.tif]

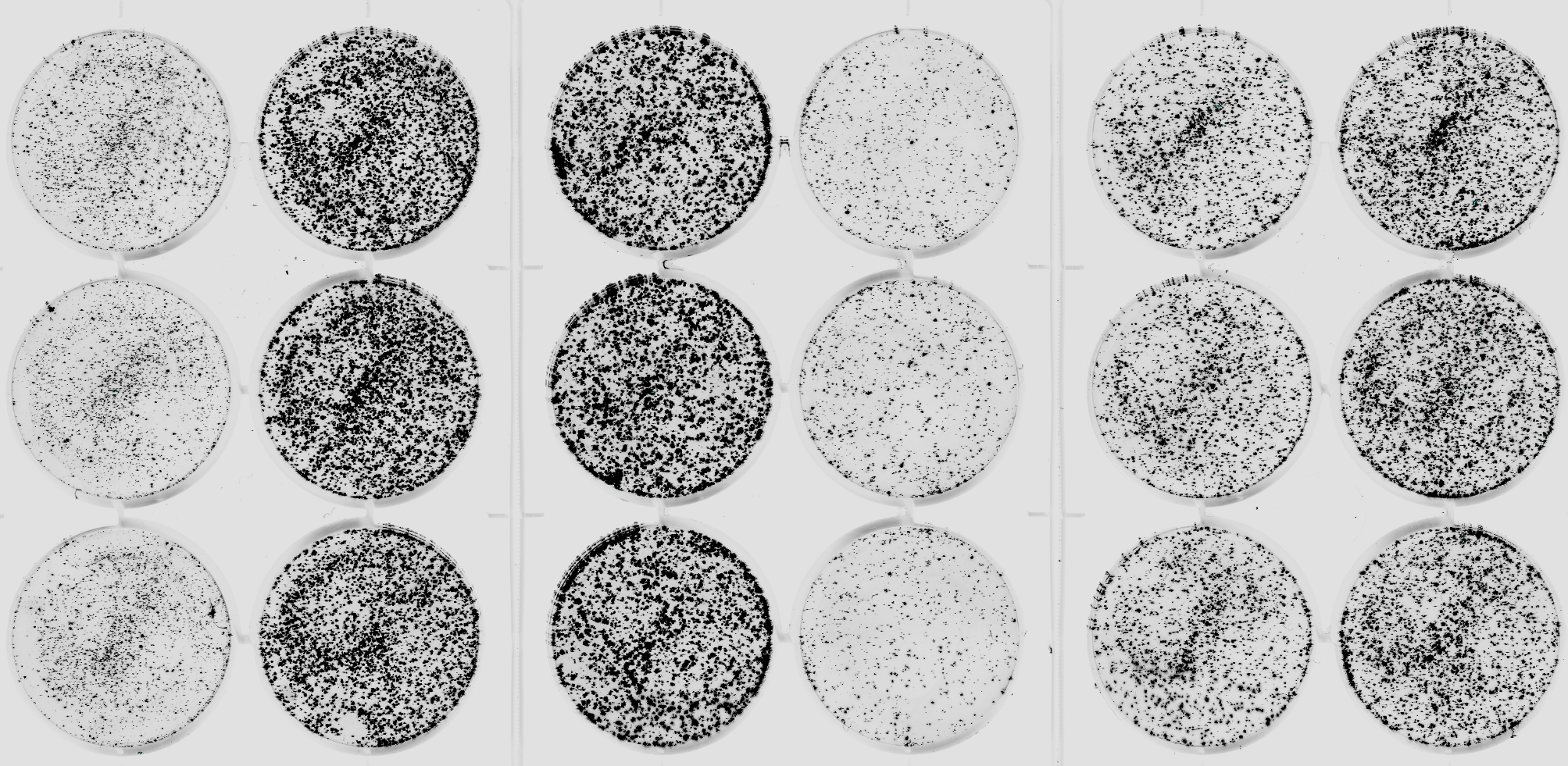

Supplement: Supplementary file 2 — Source data Fig. 1 [file 44321_2024_142_MOESM2_ESM.zip › Figure 1/E/exp#1.tif]

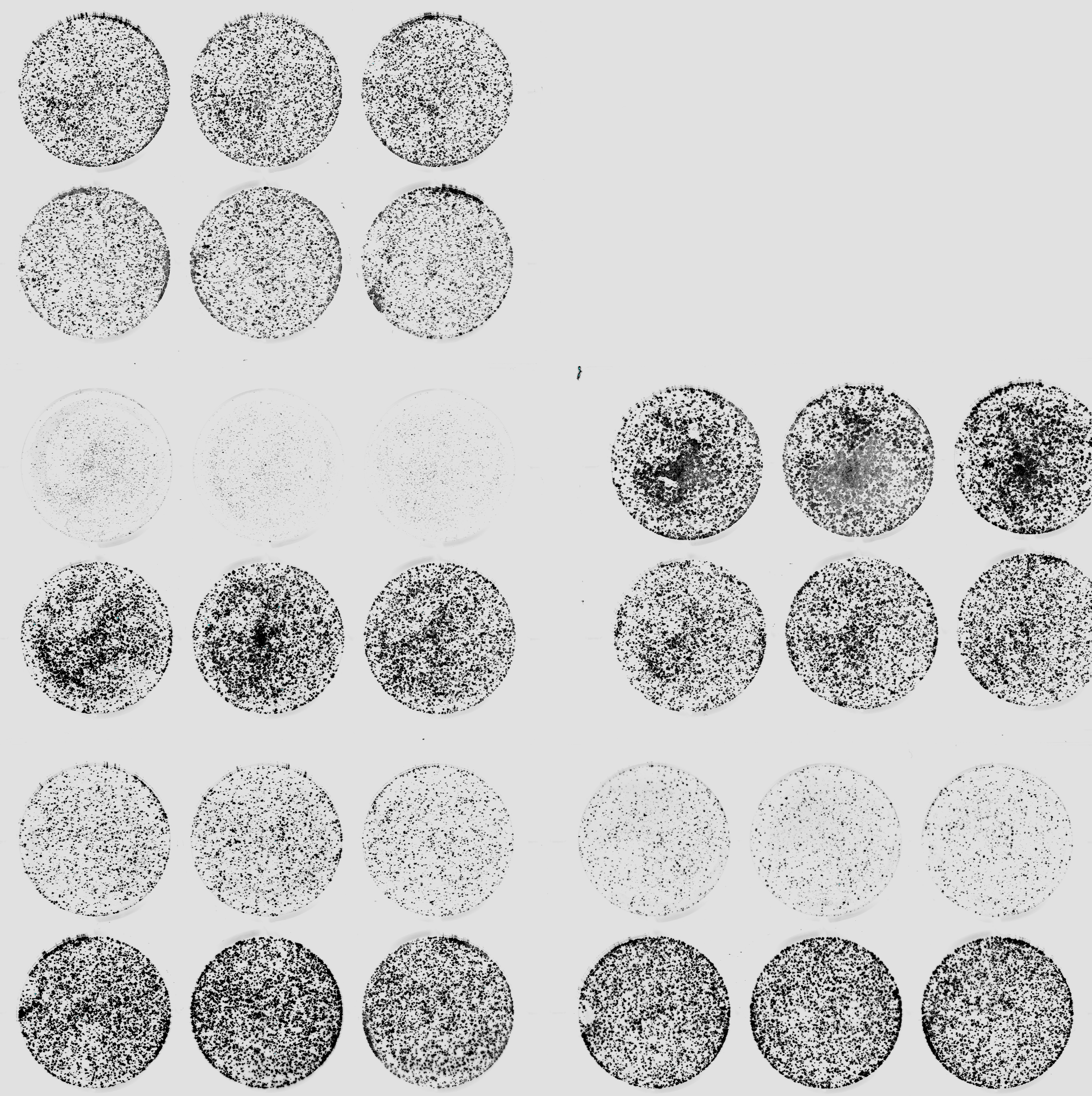

Supplement: Supplementary file 2 — Source data Fig. 1 [file 44321_2024_142_MOESM2_ESM.zip › Figure 1/E/Image_0000300_01.tif]

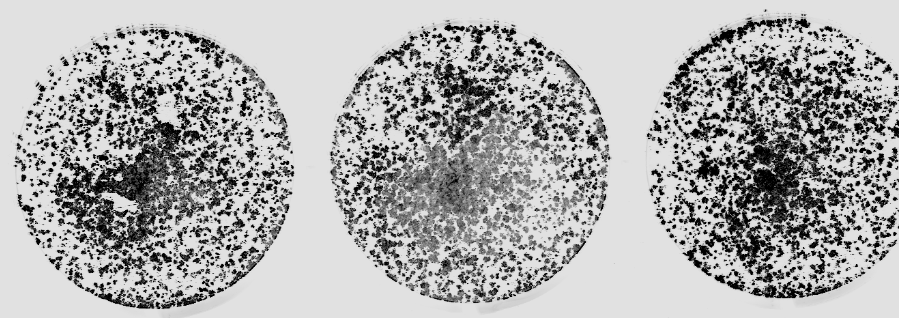

Supplement: Supplementary file 2 — Source data Fig. 1 [file 44321_2024_142_MOESM2_ESM.zip › Figure 1/E/exp#3 cal120.tif]

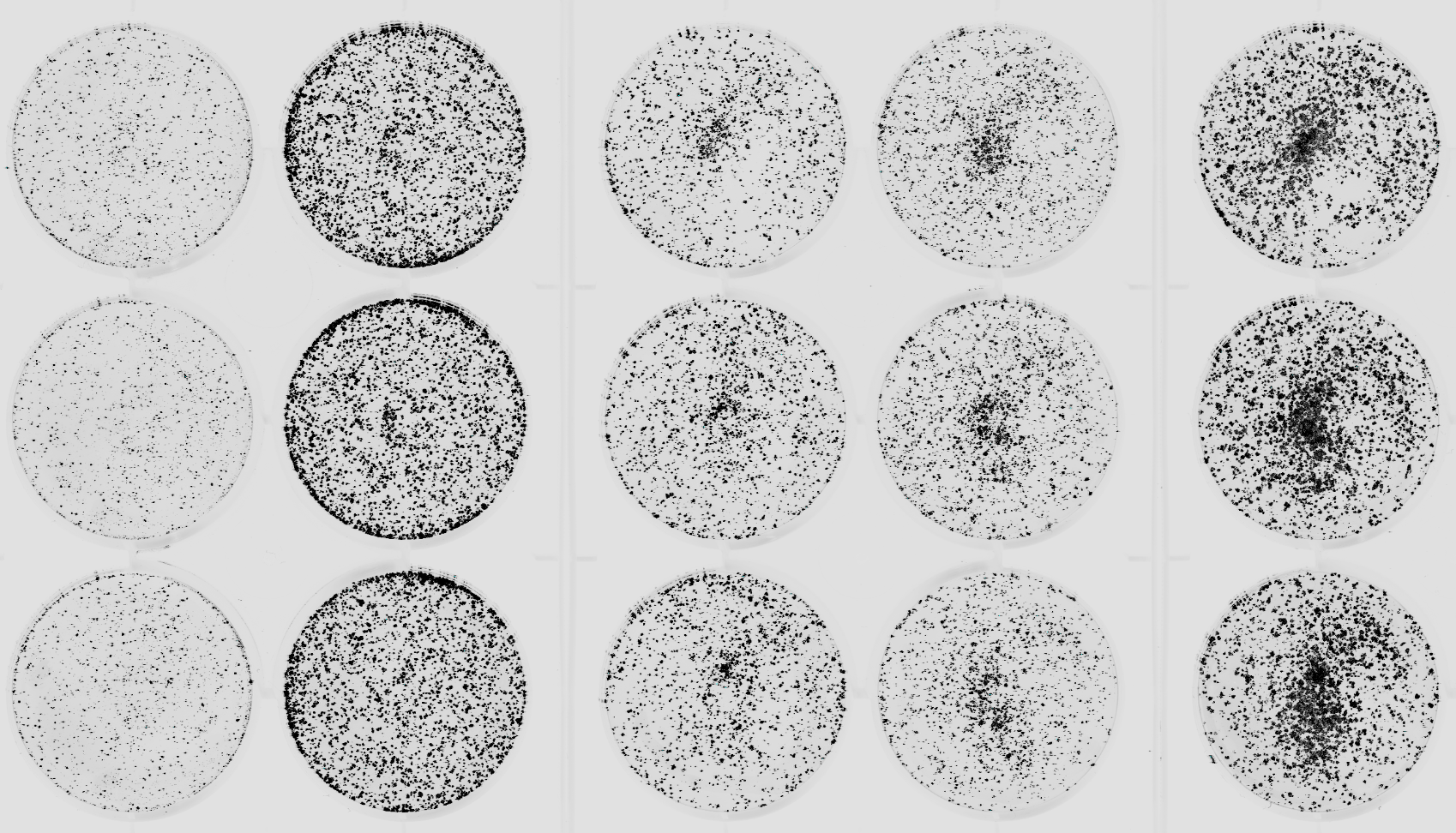

Supplement: Supplementary file 2 — Source data Fig. 1 [file 44321_2024_142_MOESM2_ESM.zip › Figure 1/E/exp#2.tif]

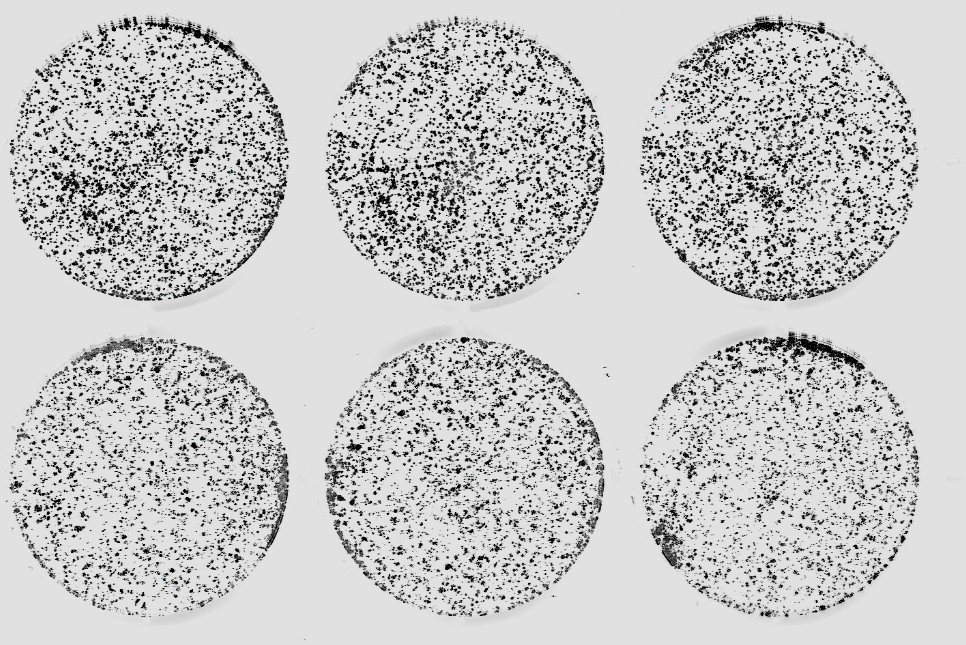

Supplement: Supplementary file 2 — Source data Fig. 1 [file 44321_2024_142_MOESM2_ESM.zip › Figure 1/E/exp#3 mcf7 Bt549.tif]

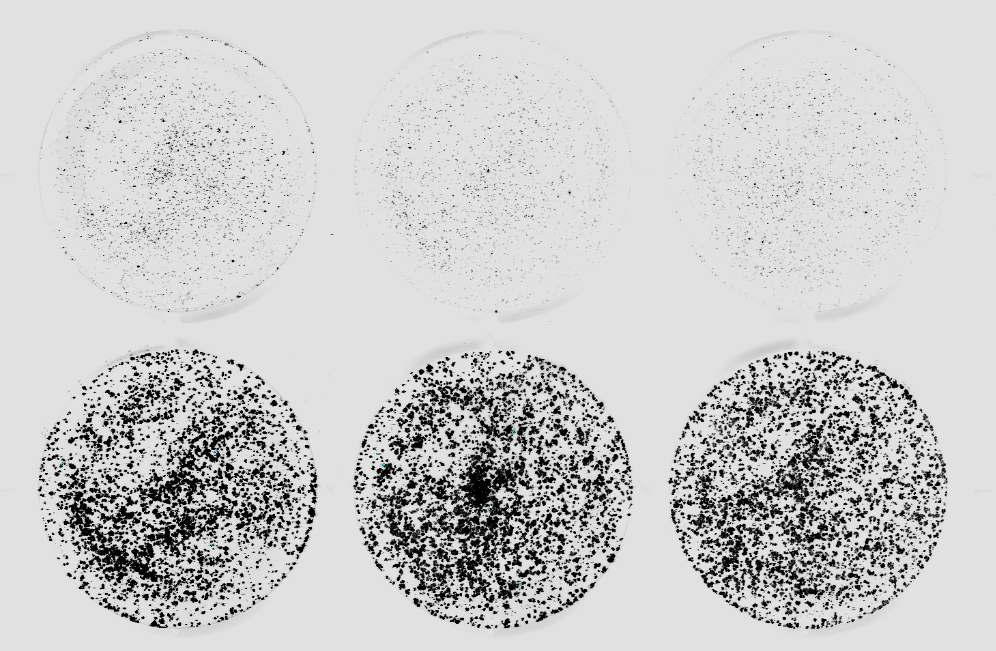

Supplement: Supplementary file 2 — Source data Fig. 1 [file 44321_2024_142_MOESM2_ESM.zip › Figure 1/E/exp#3 MM Na2SeO3.tif]

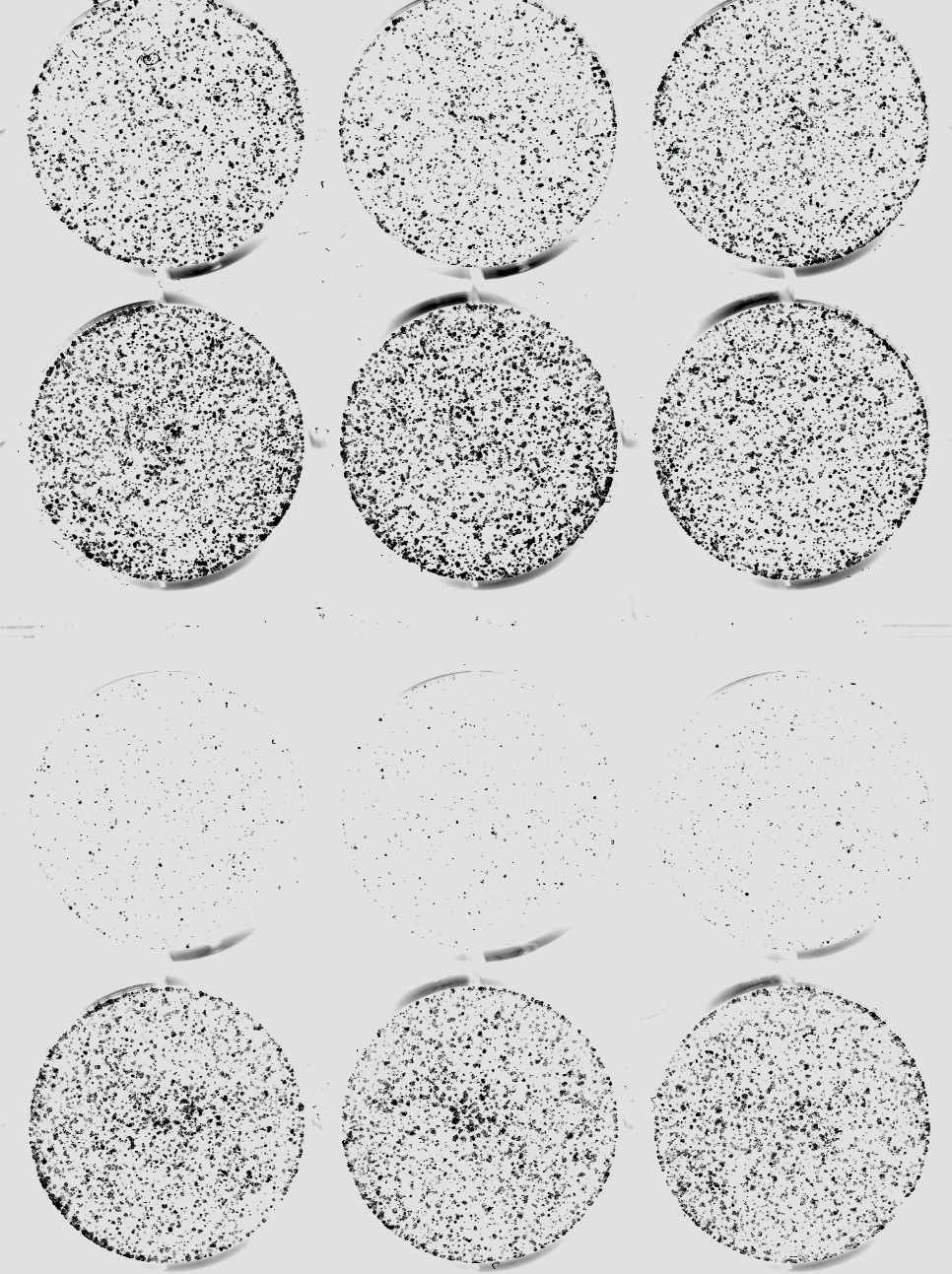

Supplement: Supplementary file 3 — Source data Fig. 2 [file 44321_2024_142_MOESM3_ESM.zip › Figure 2/E/exp#1.tif]

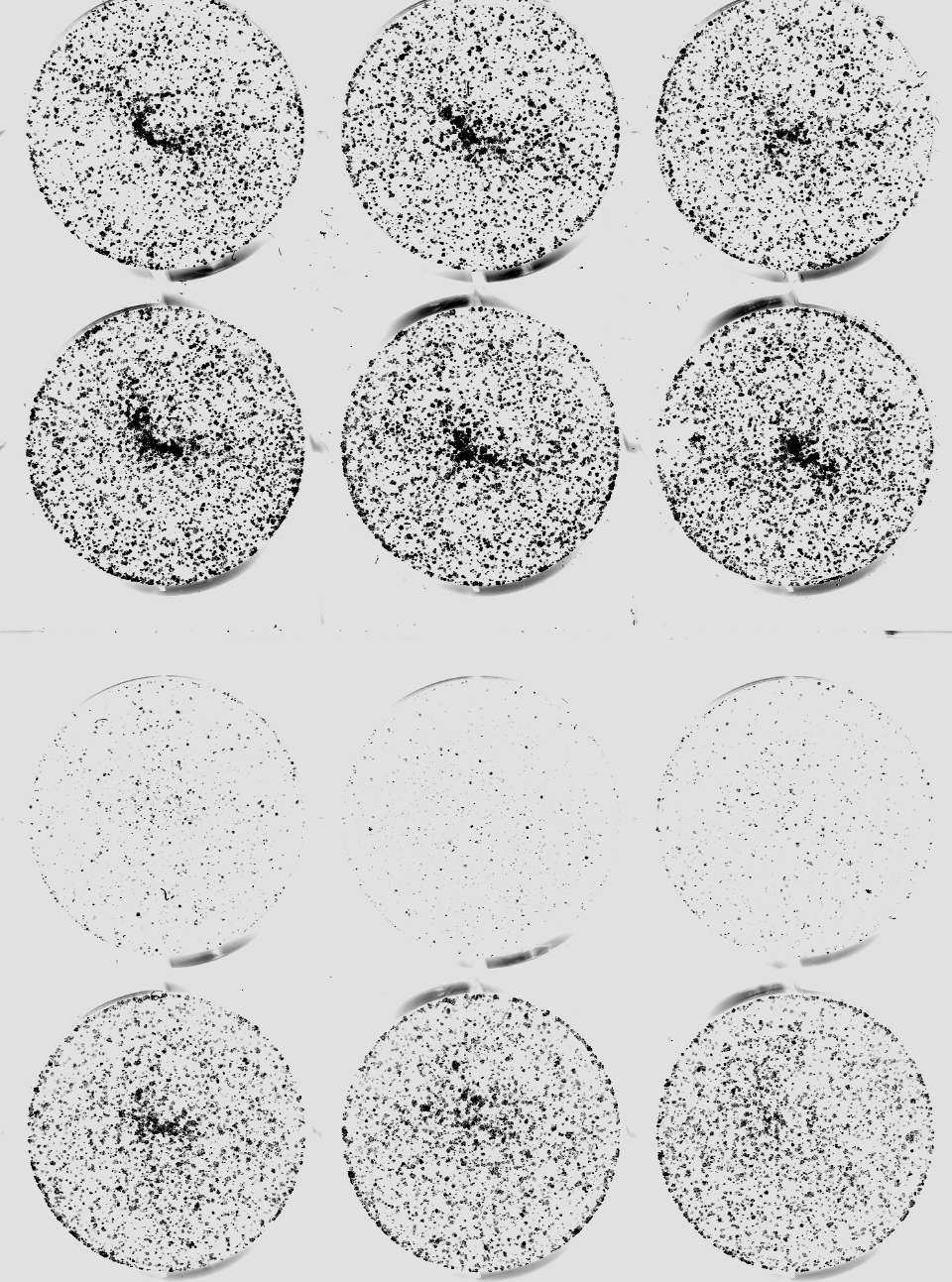

Supplement: Supplementary file 3 — Source data Fig. 2 [file 44321_2024_142_MOESM3_ESM.zip › Figure 2/E/exp#2.tif]

## Slide 1
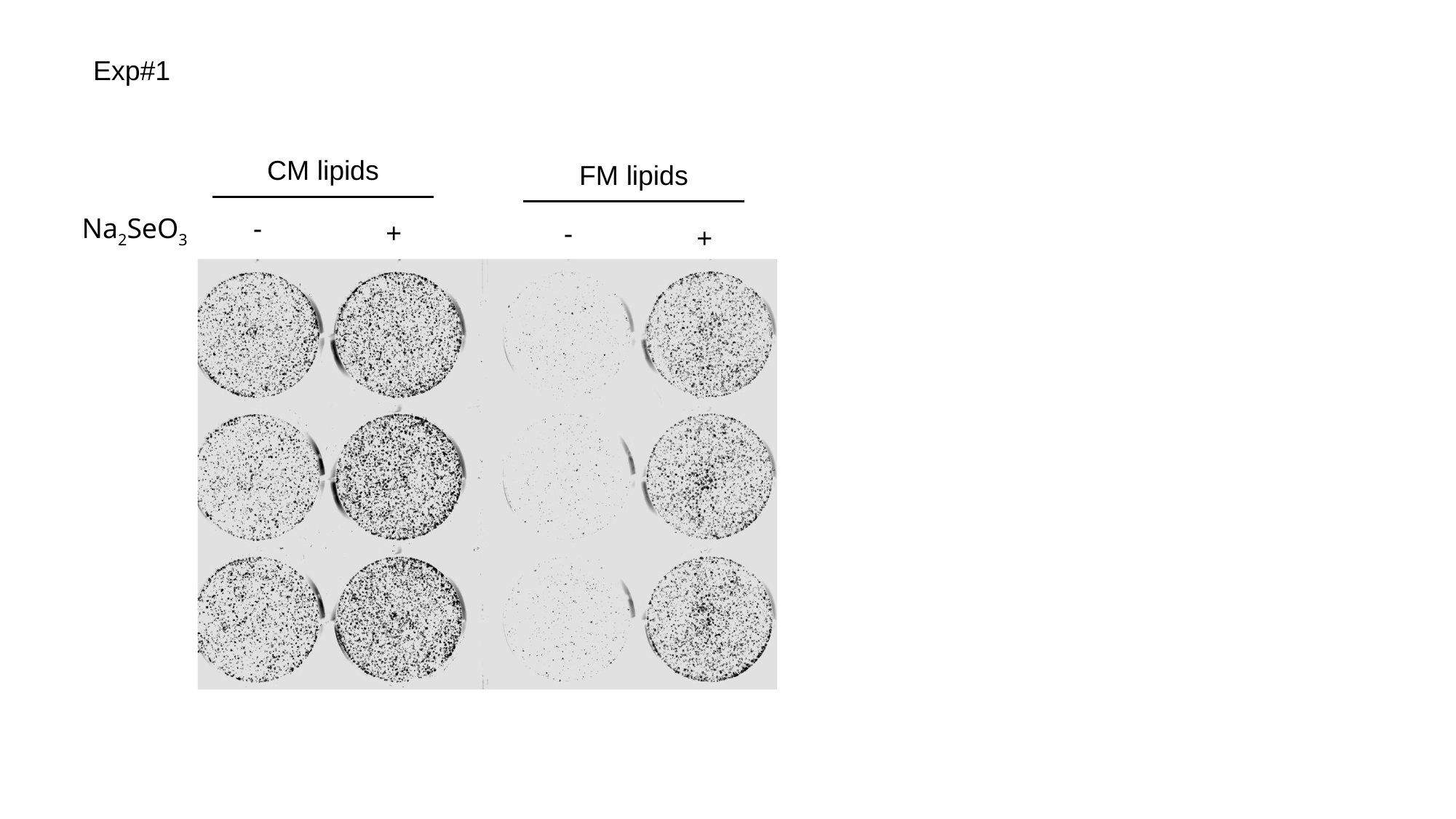

Exp#1
CM lipids
FM lipids
Na2SeO3
-
+
-
+

## Slide 2
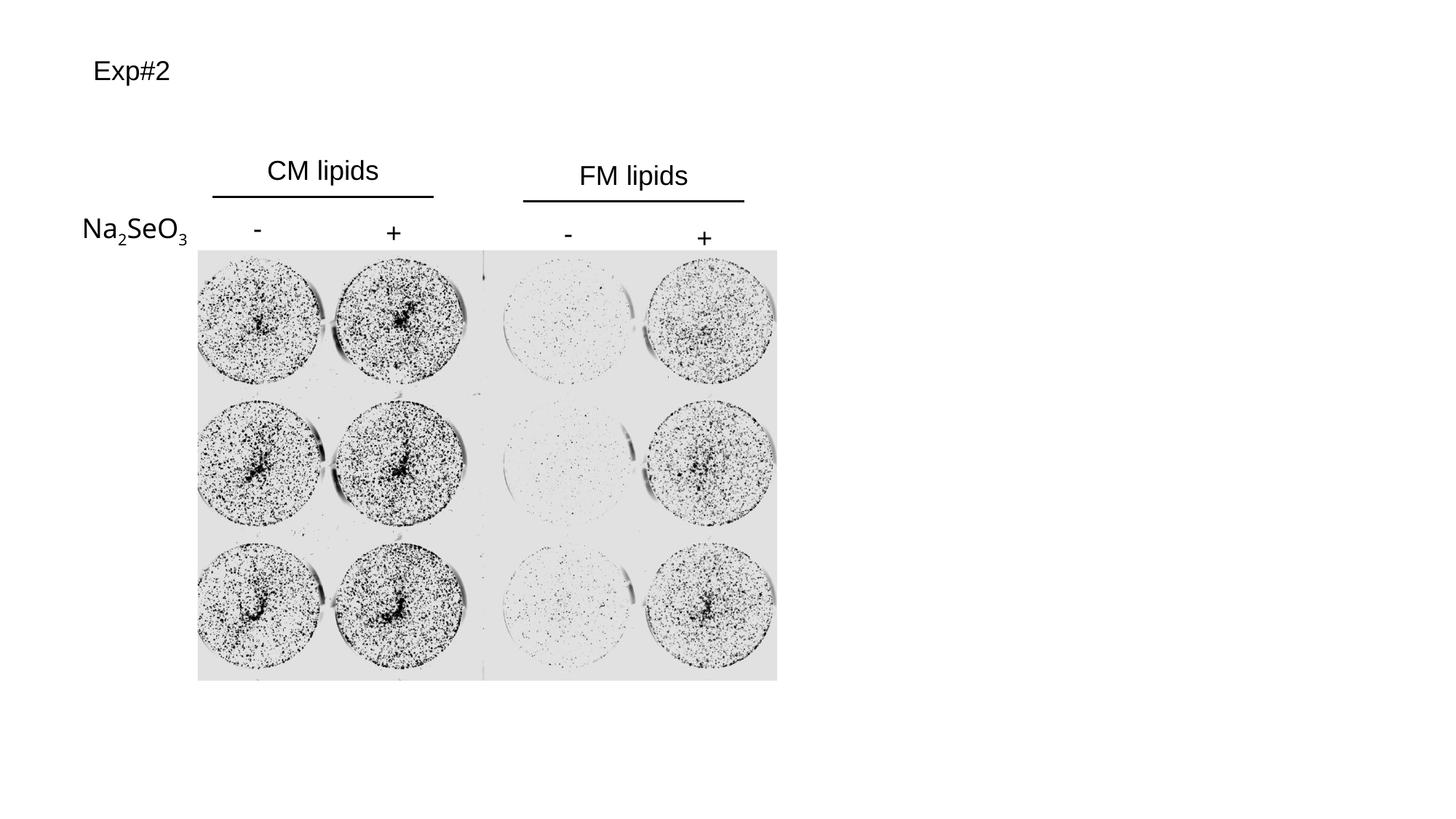

Exp#2
CM lipids
FM lipids
Na2SeO3
-
+
-
+

Supplement: Supplementary file 3 — Source data Fig. 2 [file 44321_2024_142_MOESM3_ESM.zip › Figure 2/E/pictures and labels.pptx]

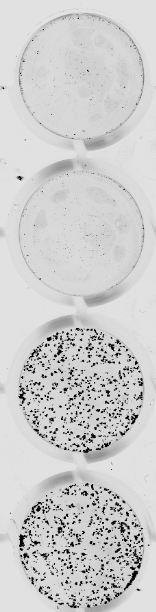

Supplement: Supplementary file 3 — Source data Fig. 2 [file 44321_2024_142_MOESM3_ESM.zip › Figure 2/G/raw pictures/exp1 ASCL3ko#2 MM.tif]

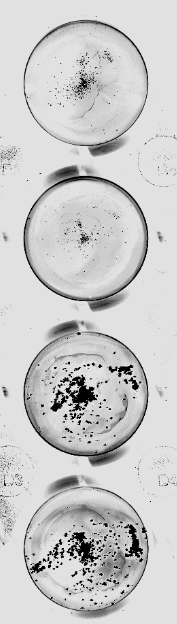

Supplement: Supplementary file 3 — Source data Fig. 2 [file 44321_2024_142_MOESM3_ESM.zip › Figure 2/G/raw pictures/exp2 ASCL3ko#2 CM.tif]

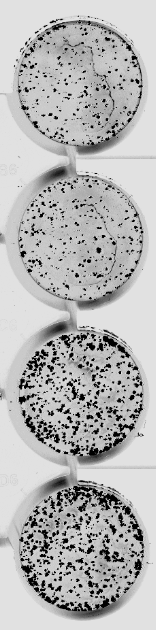

Supplement: Supplementary file 3 — Source data Fig. 2 [file 44321_2024_142_MOESM3_ESM.zip › Figure 2/G/raw pictures/exp3 NTC CM.tif]

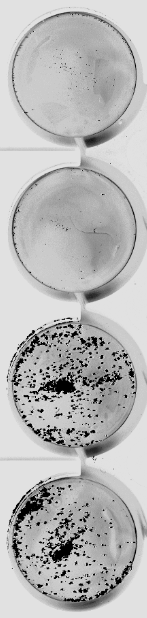

Supplement: Supplementary file 3 — Source data Fig. 2 [file 44321_2024_142_MOESM3_ESM.zip › Figure 2/G/raw pictures/exp3 ASCL3ko#1 MM.tif]

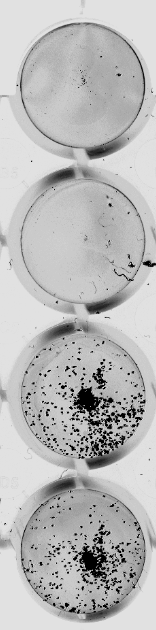

Supplement: Supplementary file 3 — Source data Fig. 2 [file 44321_2024_142_MOESM3_ESM.zip › Figure 2/G/raw pictures/exp3 ASCL3ko#2 CM.tif]

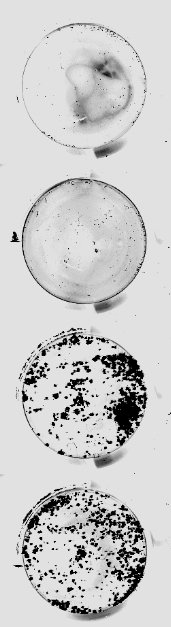

Supplement: Supplementary file 3 — Source data Fig. 2 [file 44321_2024_142_MOESM3_ESM.zip › Figure 2/G/raw pictures/exp2 NTC MM.tif]

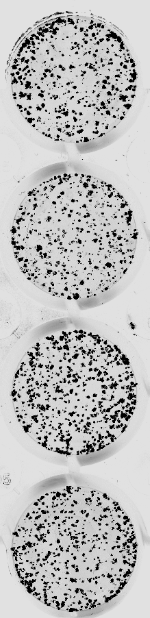

Supplement: Supplementary file 3 — Source data Fig. 2 [file 44321_2024_142_MOESM3_ESM.zip › Figure 2/G/raw pictures/exp1 NTC CM.tif]

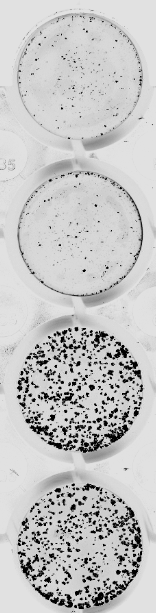

Supplement: Supplementary file 3 — Source data Fig. 2 [file 44321_2024_142_MOESM3_ESM.zip › Figure 2/G/raw pictures/exp1 ASCL3ko#1 CM.tif]

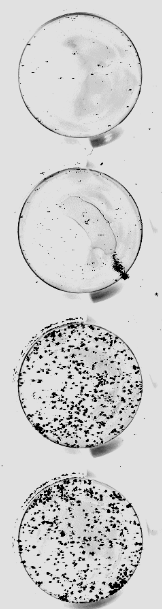

Supplement: Supplementary file 3 — Source data Fig. 2 [file 44321_2024_142_MOESM3_ESM.zip › Figure 2/G/raw pictures/exp2 ASCL3ko#1 MM.tif]

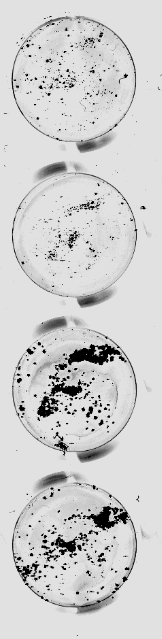

Supplement: Supplementary file 3 — Source data Fig. 2 [file 44321_2024_142_MOESM3_ESM.zip › Figure 2/G/raw pictures/exp2 NTC CM.tif]

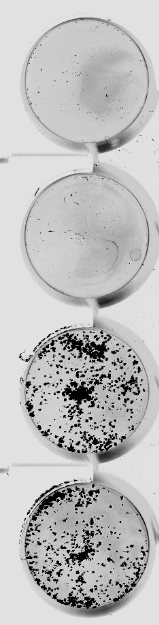

Supplement: Supplementary file 3 — Source data Fig. 2 [file 44321_2024_142_MOESM3_ESM.zip › Figure 2/G/raw pictures/exp3 ASCL3ko#2 MM.tif]

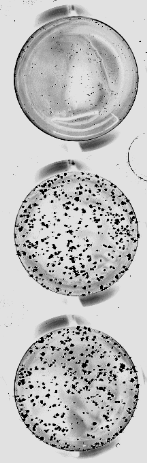

Supplement: Supplementary file 3 — Source data Fig. 2 [file 44321_2024_142_MOESM3_ESM.zip › Figure 2/G/raw pictures/exp2 ASCL3ko#1 CM.tif]

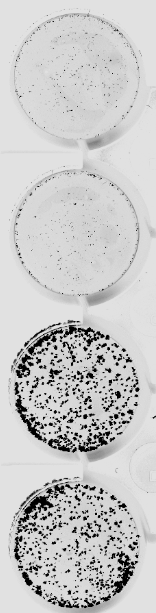

Supplement: Supplementary file 3 — Source data Fig. 2 [file 44321_2024_142_MOESM3_ESM.zip › Figure 2/G/raw pictures/exp1 ASCL3ko#1 MM.tif]

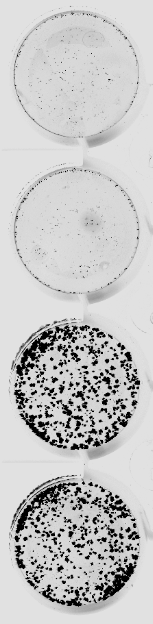

Supplement: Supplementary file 3 — Source data Fig. 2 [file 44321_2024_142_MOESM3_ESM.zip › Figure 2/G/raw pictures/exp1 NTC MM.tif]

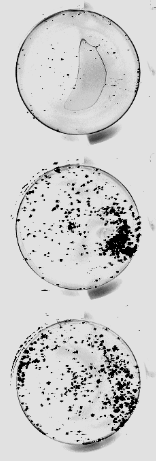

Supplement: Supplementary file 3 — Source data Fig. 2 [file 44321_2024_142_MOESM3_ESM.zip › Figure 2/G/raw pictures/exp2 ASCL3ko#2 MM.tif]

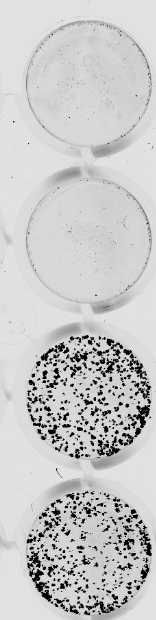

Supplement: Supplementary file 3 — Source data Fig. 2 [file 44321_2024_142_MOESM3_ESM.zip › Figure 2/G/raw pictures/exp1 ASCL3ko#2 CM.tif]

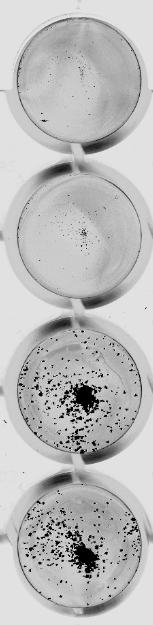

Supplement: Supplementary file 3 — Source data Fig. 2 [file 44321_2024_142_MOESM3_ESM.zip › Figure 2/G/raw pictures/exp3 ASCL3ko#1 CM.tif]

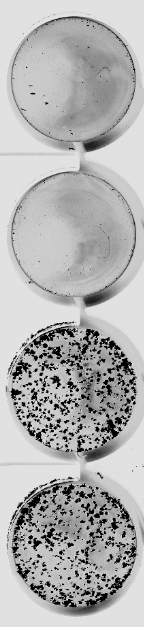

Supplement: Supplementary file 3 — Source data Fig. 2 [file 44321_2024_142_MOESM3_ESM.zip › Figure 2/G/raw pictures/exp3 NTC MM.tif]

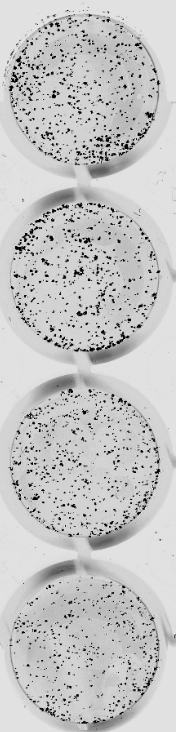

Supplement: Supplementary file 3 — Source data Fig. 2 [file 44321_2024_142_MOESM3_ESM.zip › Figure 2/A/pictures/exp#2 DMEMF-12 CM.tif]

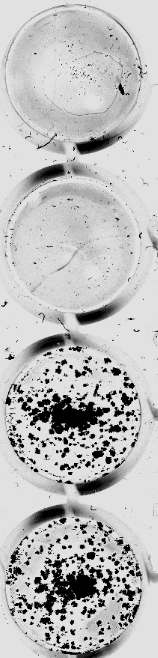

Supplement: Supplementary file 3 — Source data Fig. 2 [file 44321_2024_142_MOESM3_ESM.zip › Figure 2/A/pictures/exp#1 DMEMF-12 FM.tif]

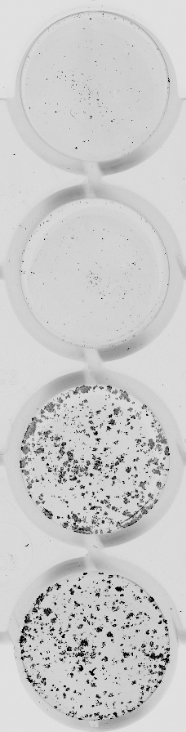

Supplement: Supplementary file 3 — Source data Fig. 2 [file 44321_2024_142_MOESM3_ESM.zip › Figure 2/A/pictures/exp#3 Plasmax FM.tif]

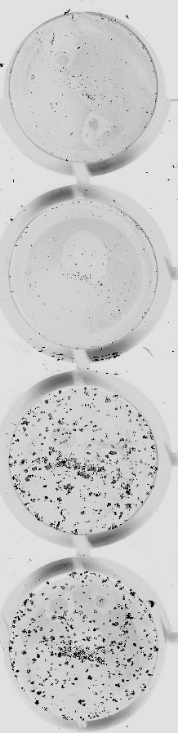

Supplement: Supplementary file 3 — Source data Fig. 2 [file 44321_2024_142_MOESM3_ESM.zip › Figure 2/A/pictures/exp#2 plasmax FM.tif]

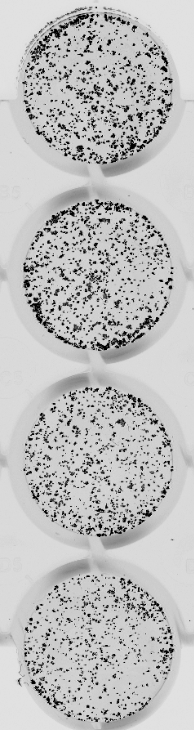

Supplement: Supplementary file 3 — Source data Fig. 2 [file 44321_2024_142_MOESM3_ESM.zip › Figure 2/A/pictures/exp#3 DMEMF-12 CM.tif]

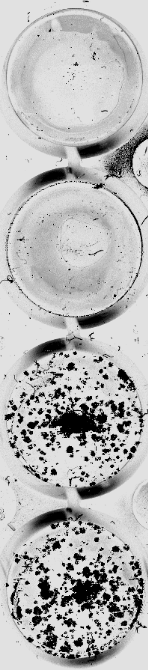

Supplement: Supplementary file 3 — Source data Fig. 2 [file 44321_2024_142_MOESM3_ESM.zip › Figure 2/A/pictures/exp#1 Plasmax FM.tif]

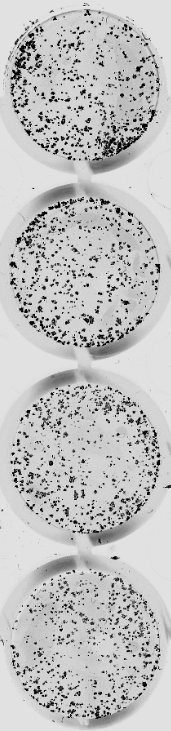

Supplement: Supplementary file 3 — Source data Fig. 2 [file 44321_2024_142_MOESM3_ESM.zip › Figure 2/A/pictures/exp#2 plasmax CM.tif]

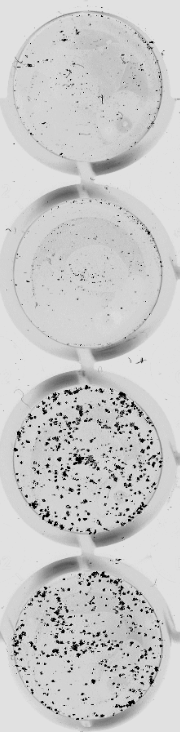

Supplement: Supplementary file 3 — Source data Fig. 2 [file 44321_2024_142_MOESM3_ESM.zip › Figure 2/A/pictures/exp#2 DMEMF-12 FM.tif]

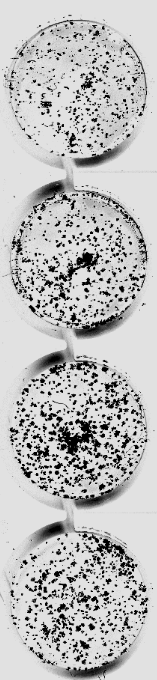

Supplement: Supplementary file 3 — Source data Fig. 2 [file 44321_2024_142_MOESM3_ESM.zip › Figure 2/A/pictures/exp#1 DMEMF-12 CM.tif]

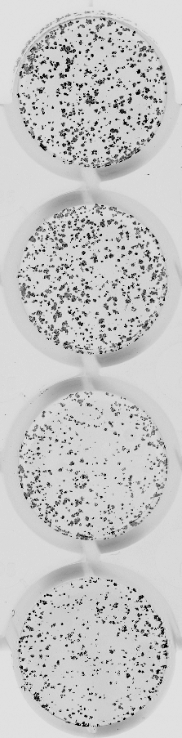

Supplement: Supplementary file 3 — Source data Fig. 2 [file 44321_2024_142_MOESM3_ESM.zip › Figure 2/A/pictures/exp#3 Plasmax CM.tif]

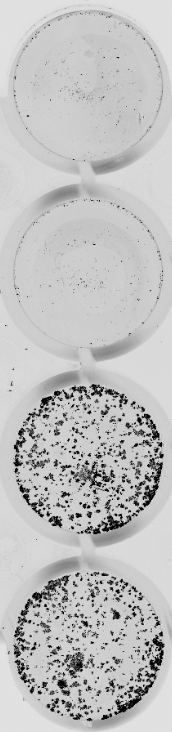

Supplement: Supplementary file 3 — Source data Fig. 2 [file 44321_2024_142_MOESM3_ESM.zip › Figure 2/A/pictures/exp#3 DMEMF-12 FM.tif]

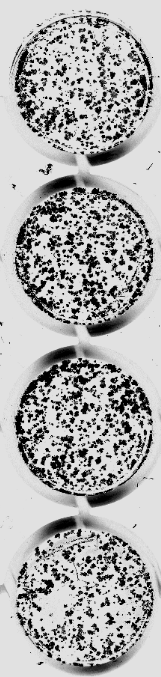

Supplement: Supplementary file 3 — Source data Fig. 2 [file 44321_2024_142_MOESM3_ESM.zip › Figure 2/A/pictures/exp#1 Plasmax CM.tif]

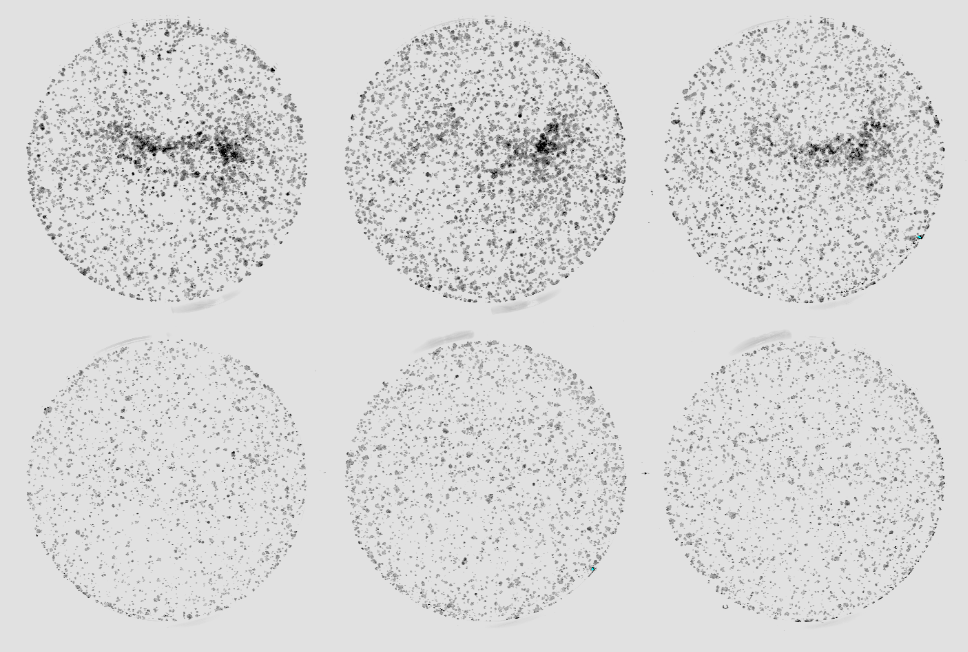

Supplement: Supplementary file 3 — Source data Fig. 2 [file 44321_2024_142_MOESM3_ESM.zip › Figure 2/C/pictures/exp#3 fractionated CM.tif]

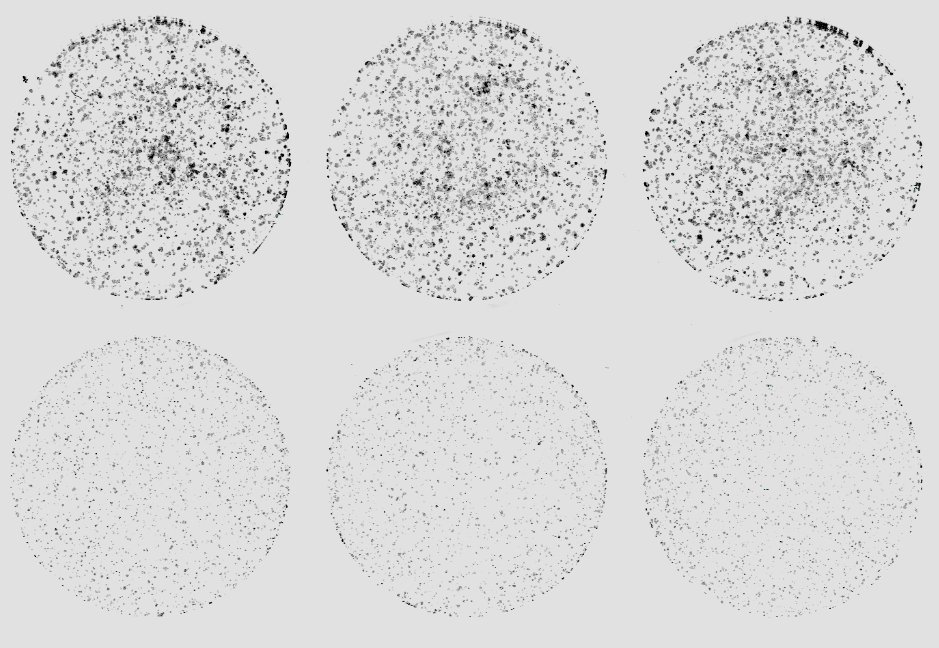

Supplement: Supplementary file 3 — Source data Fig. 2 [file 44321_2024_142_MOESM3_ESM.zip › Figure 2/C/pictures/exp#2 fractionated CM.tif]

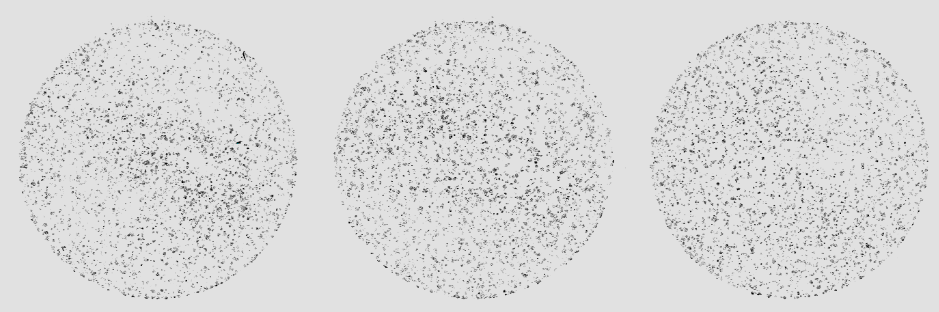

Supplement: Supplementary file 3 — Source data Fig. 2 [file 44321_2024_142_MOESM3_ESM.zip › Figure 2/C/pictures/exp#1 CM.tif]

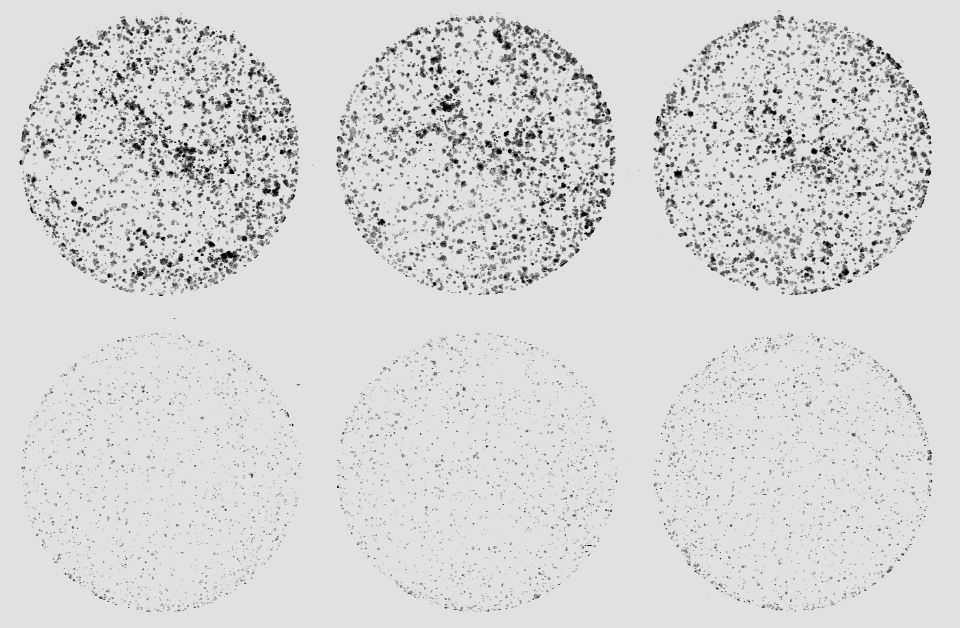

Supplement: Supplementary file 3 — Source data Fig. 2 [file 44321_2024_142_MOESM3_ESM.zip › Figure 2/C/pictures/exp#1 fractionated CM.tif]

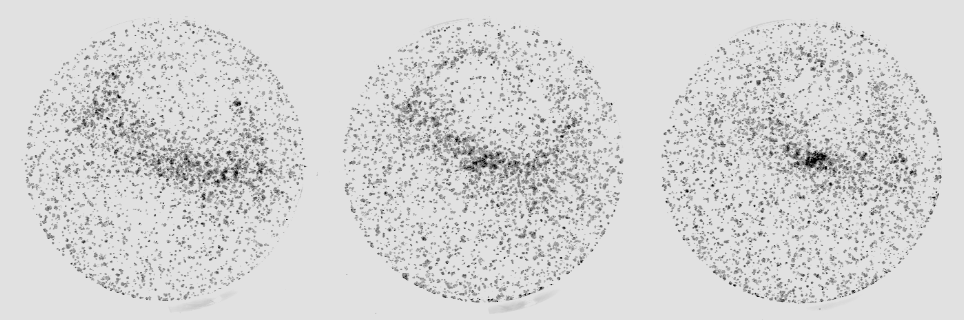

Supplement: Supplementary file 3 — Source data Fig. 2 [file 44321_2024_142_MOESM3_ESM.zip › Figure 2/C/pictures/exp#3 CM.tif]

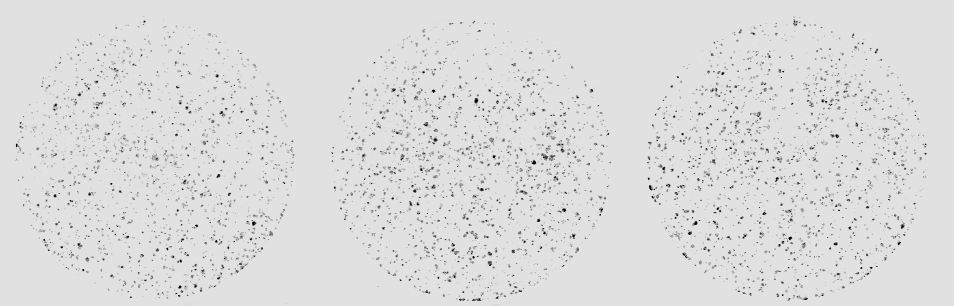

Supplement: Supplementary file 3 — Source data Fig. 2 [file 44321_2024_142_MOESM3_ESM.zip › Figure 2/C/pictures/exp#2 MM.tif]

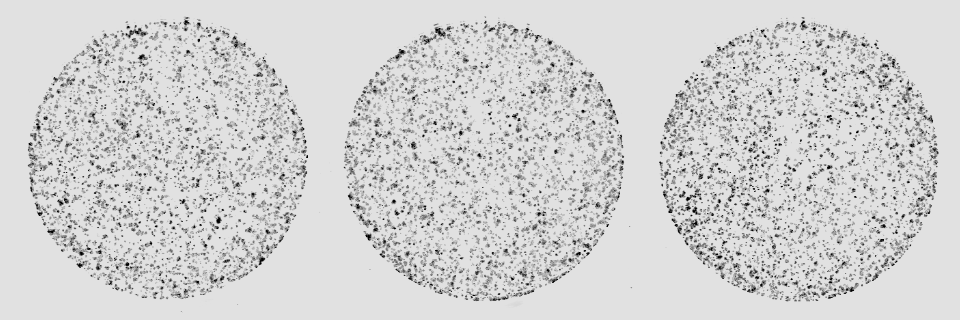

Supplement: Supplementary file 3 — Source data Fig. 2 [file 44321_2024_142_MOESM3_ESM.zip › Figure 2/C/pictures/exp#2 CM.tif]

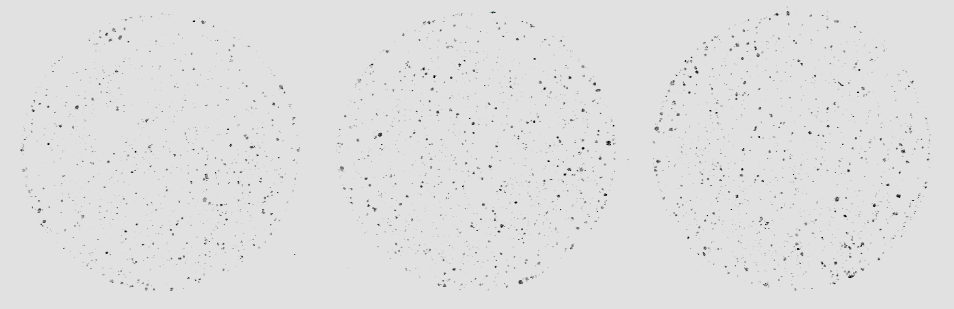

Supplement: Supplementary file 3 — Source data Fig. 2 [file 44321_2024_142_MOESM3_ESM.zip › Figure 2/C/pictures/exp#1 MM.tif]

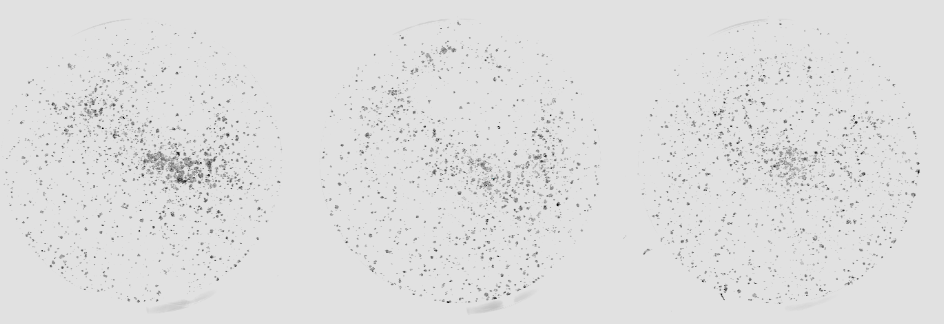

Supplement: Supplementary file 3 — Source data Fig. 2 [file 44321_2024_142_MOESM3_ESM.zip › Figure 2/C/pictures/exp#3 MM.tif]

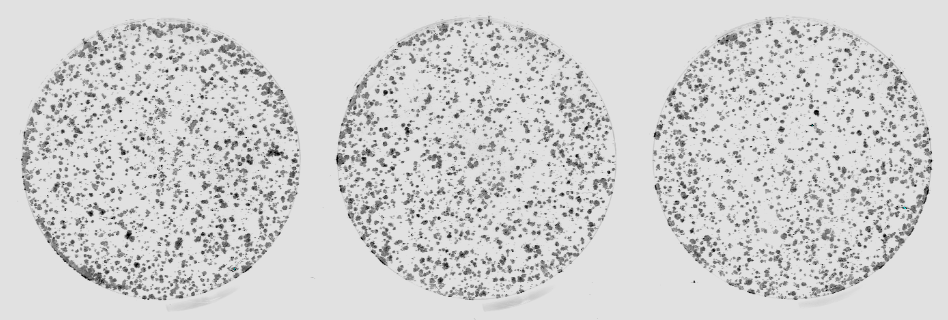

Supplement: Supplementary file 3 — Source data Fig. 2 [file 44321_2024_142_MOESM3_ESM.zip › Figure 2/D/pictures/exp#1 95C.tif]

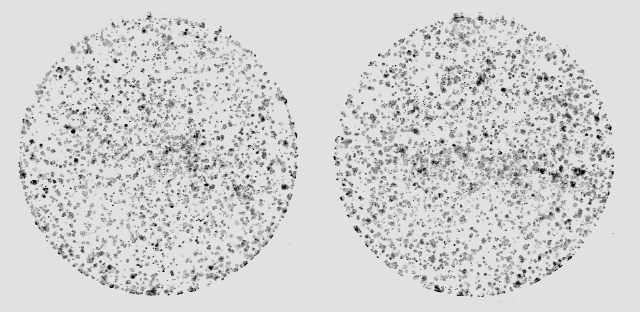

Supplement: Supplementary file 3 — Source data Fig. 2 [file 44321_2024_142_MOESM3_ESM.zip › Figure 2/D/pictures/exp#2 95C.tif]

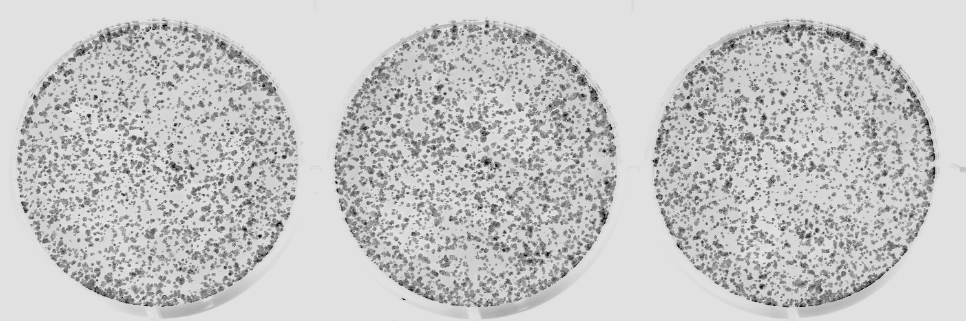

Supplement: Supplementary file 3 — Source data Fig. 2 [file 44321_2024_142_MOESM3_ESM.zip › Figure 2/D/pictures/exp#3 RT.tif]

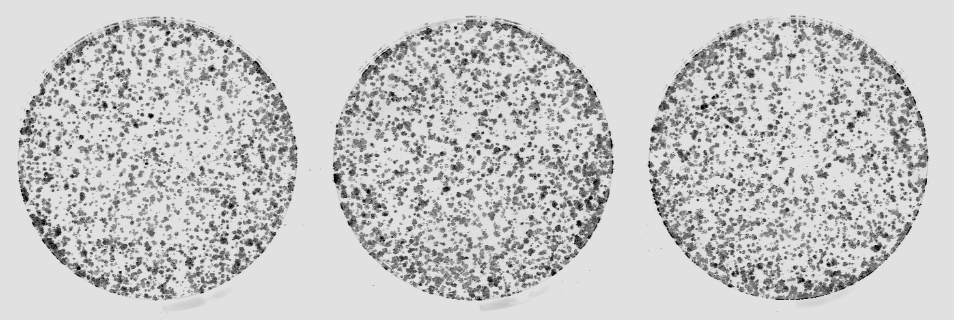

Supplement: Supplementary file 3 — Source data Fig. 2 [file 44321_2024_142_MOESM3_ESM.zip › Figure 2/D/pictures/exp#1 RT.tif]

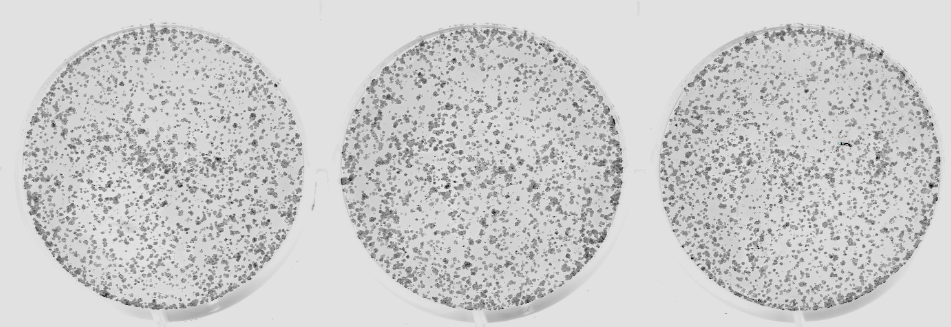

Supplement: Supplementary file 3 — Source data Fig. 2 [file 44321_2024_142_MOESM3_ESM.zip › Figure 2/D/pictures/exp#3 95C.tif]

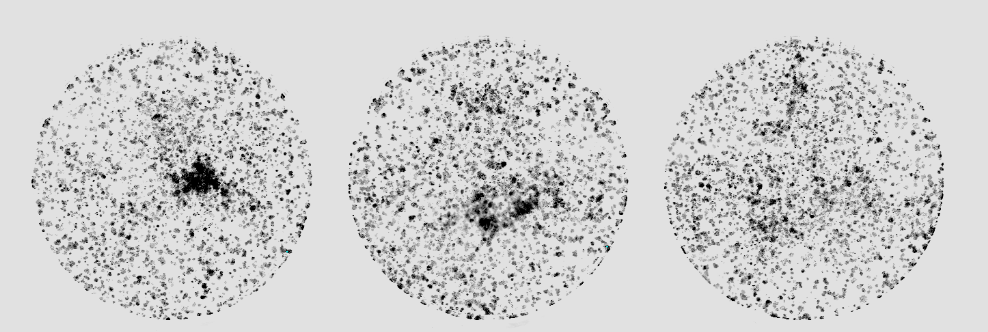

Supplement: Supplementary file 3 — Source data Fig. 2 [file 44321_2024_142_MOESM3_ESM.zip › Figure 2/D/pictures/exp#2 RT.tif]

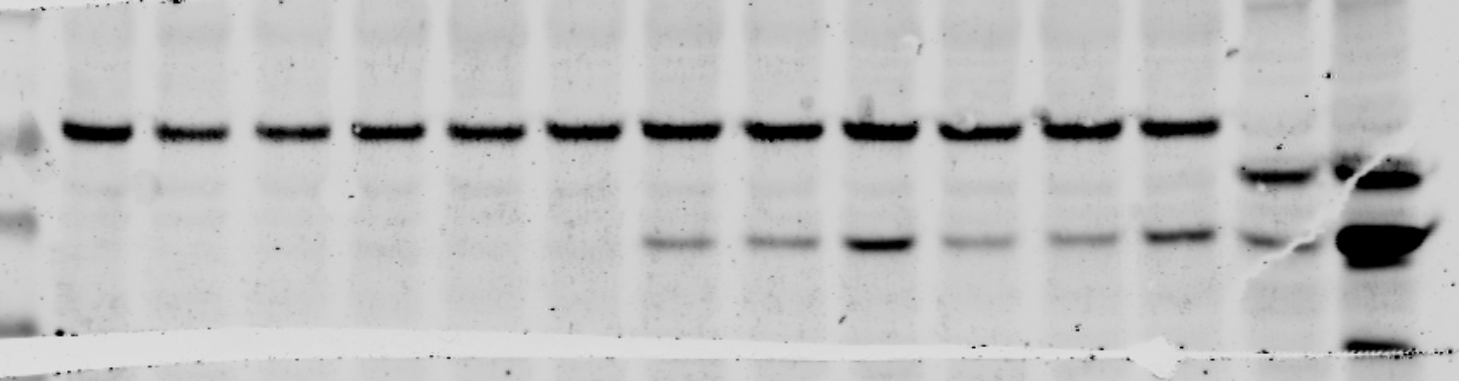

Supplement: Supplementary file 5 — Source data Fig. 4 [file 44321_2024_142_MOESM5_ESM.zip › Figure 4/H/SCD.tif]

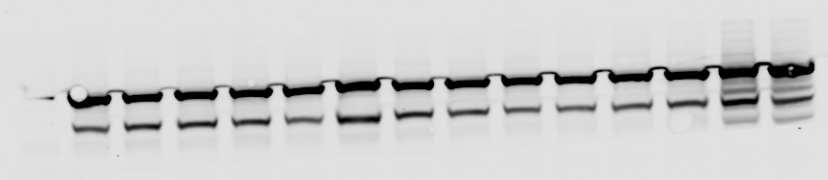

Supplement: Supplementary file 5 — Source data Fig. 4 [file 44321_2024_142_MOESM5_ESM.zip › Figure 4/H/vinculin.tif]

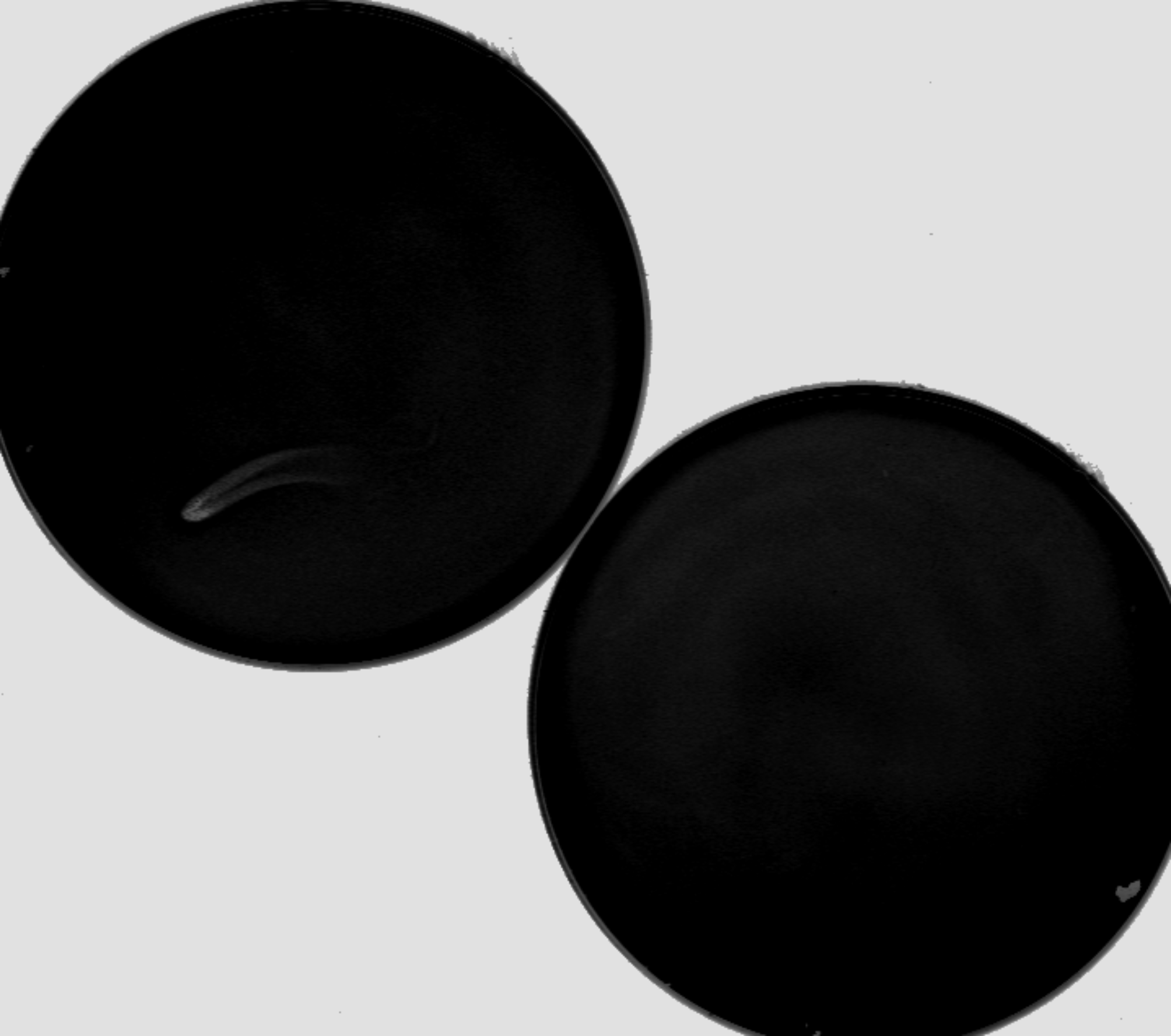

Supplement: Supplementary file 5 — Source data Fig. 4 [file 44321_2024_142_MOESM5_ESM.zip › Figure 4/B/exp4.tif]

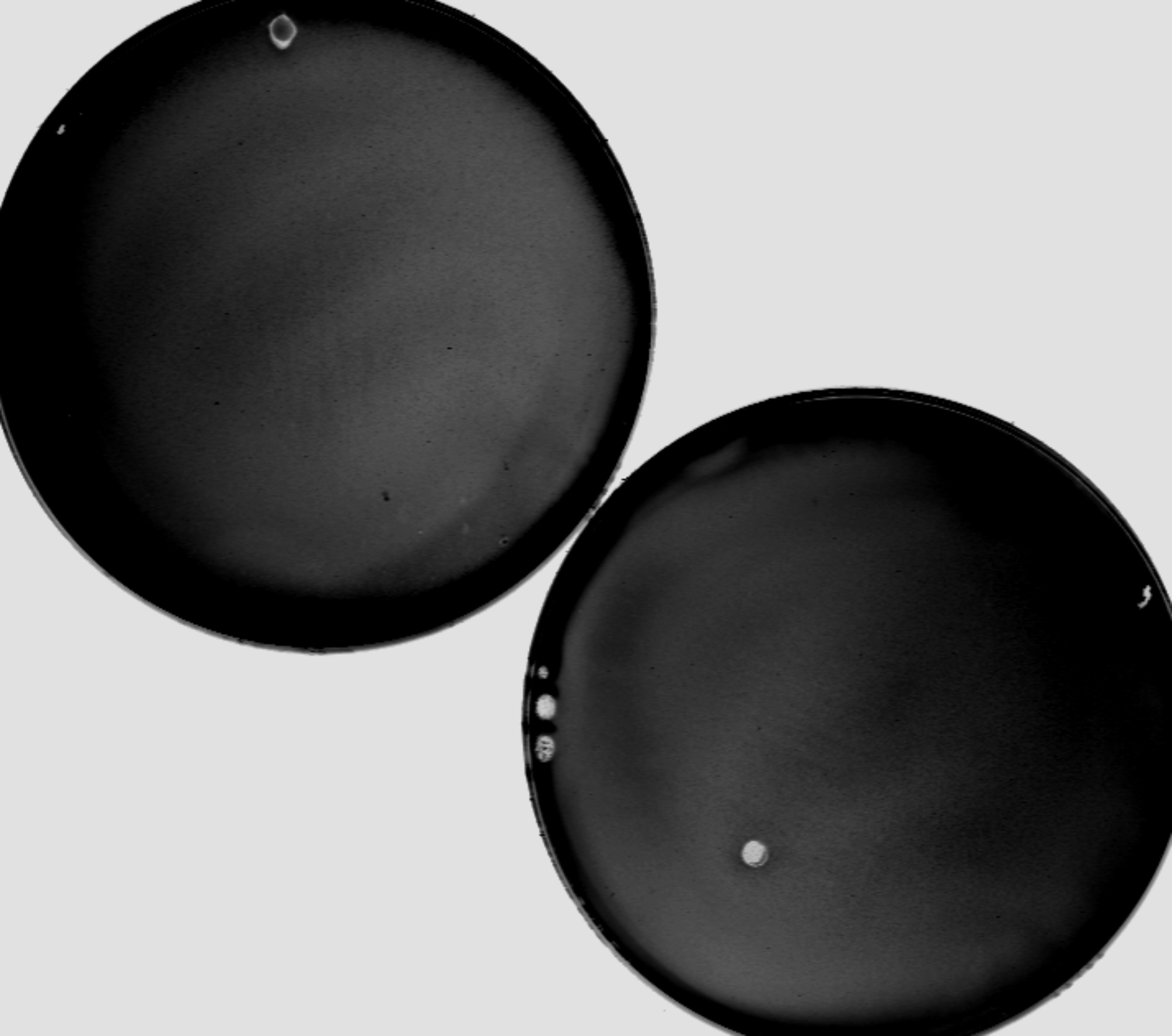

Supplement: Supplementary file 5 — Source data Fig. 4 [file 44321_2024_142_MOESM5_ESM.zip › Figure 4/B/exp2.tif]

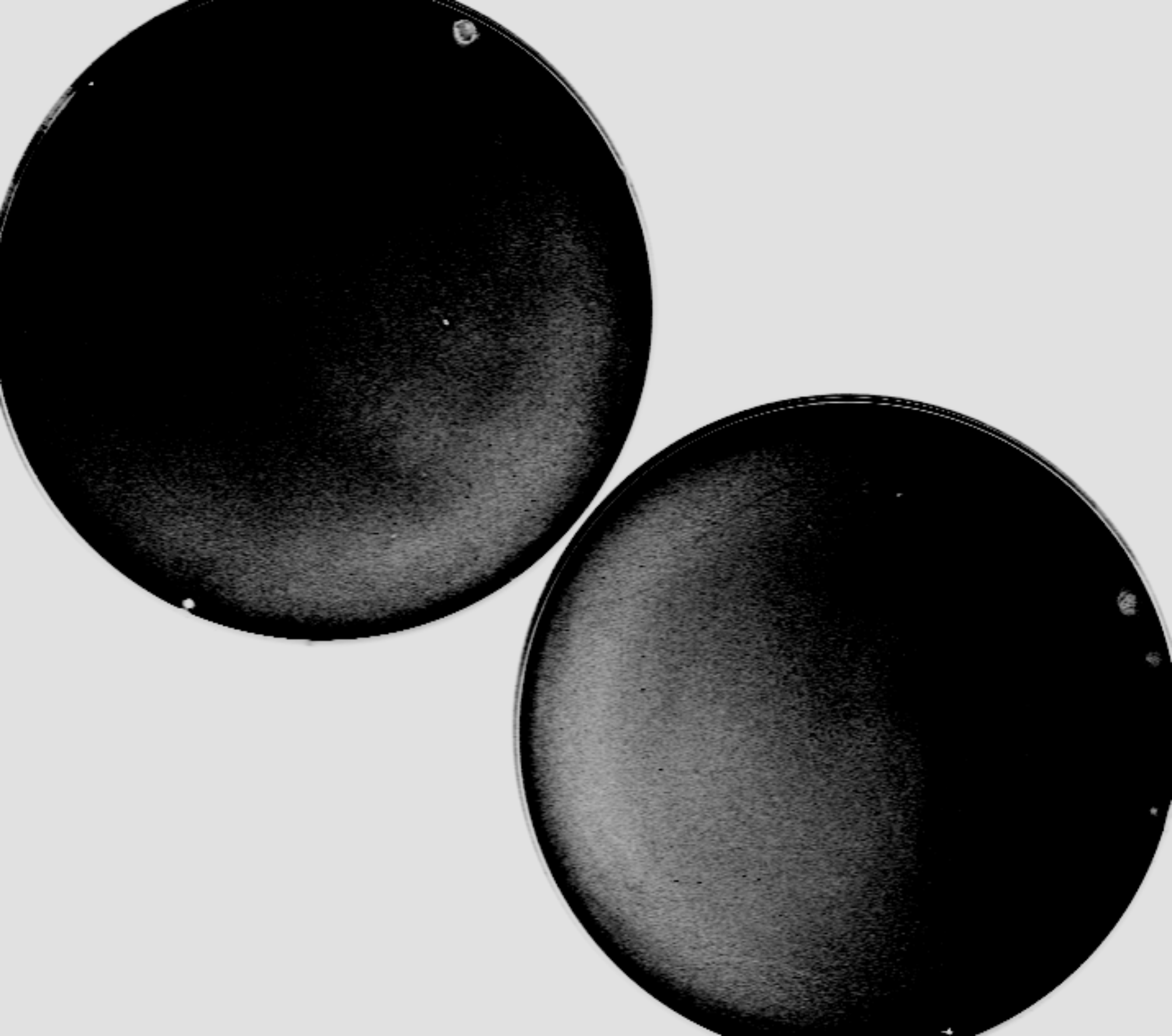

Supplement: Supplementary file 5 — Source data Fig. 4 [file 44321_2024_142_MOESM5_ESM.zip › Figure 4/B/exp3.tif]

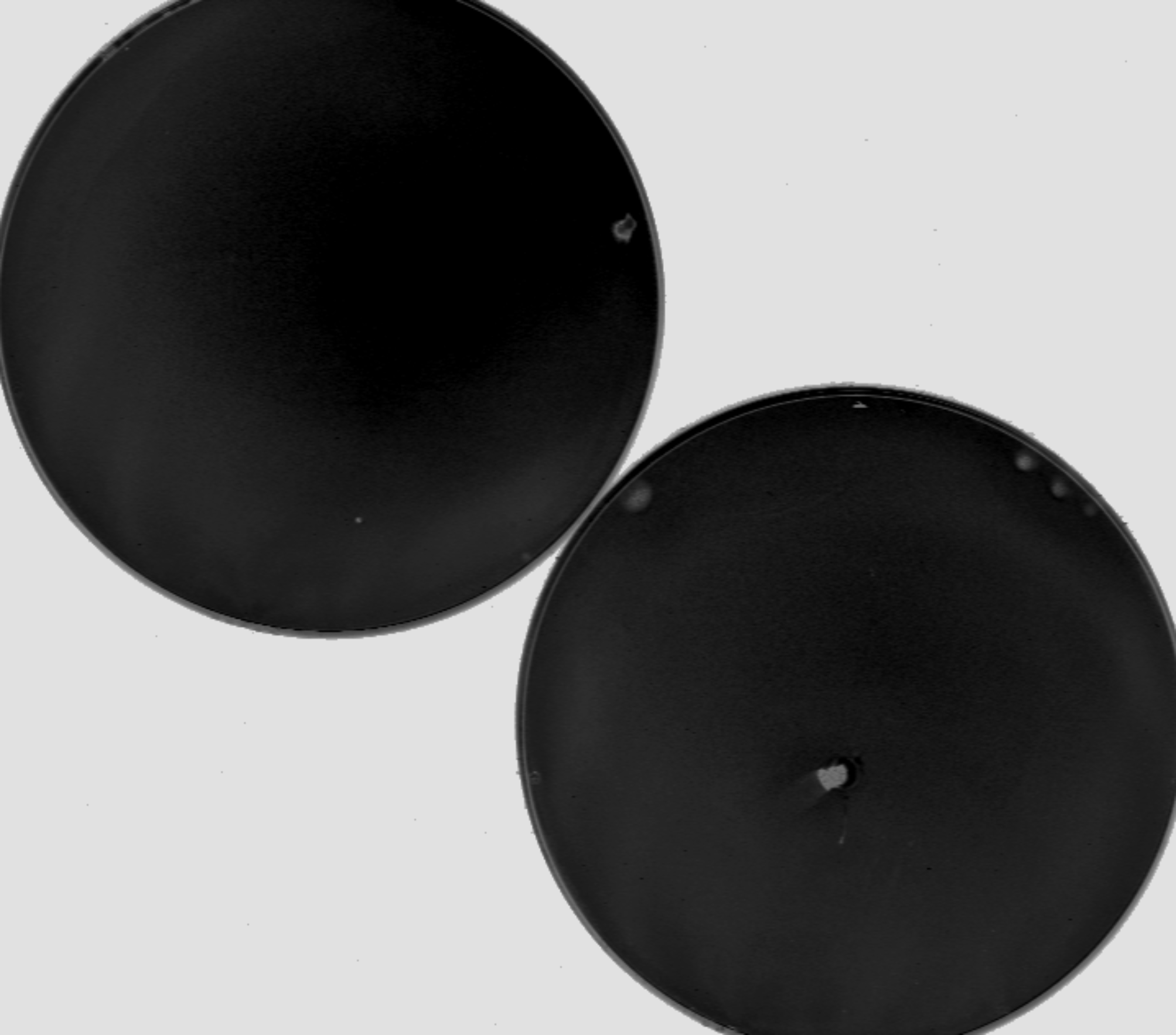

Supplement: Supplementary file 5 — Source data Fig. 4 [file 44321_2024_142_MOESM5_ESM.zip › Figure 4/B/exp1.tif]

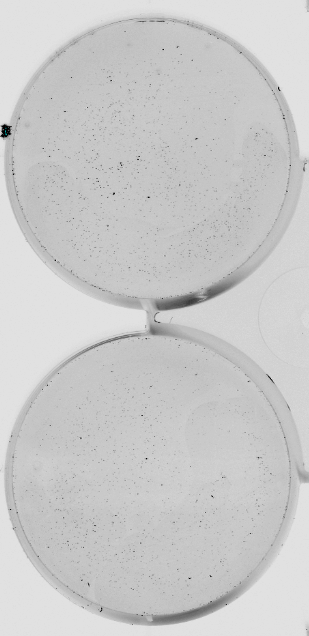

Supplement: Supplementary file 5 — Source data Fig. 4 [file 44321_2024_142_MOESM5_ESM.zip › Figure 4/G/pictures/exp3 MM.tif]

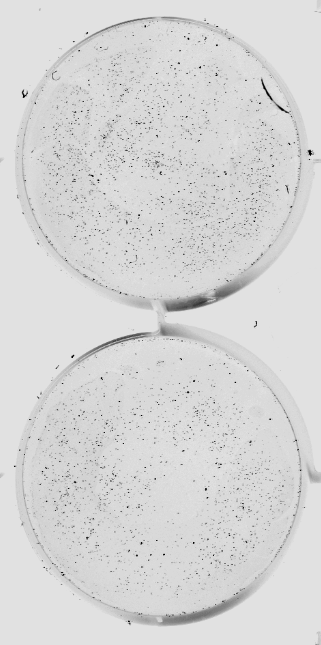

Supplement: Supplementary file 5 — Source data Fig. 4 [file 44321_2024_142_MOESM5_ESM.zip › Figure 4/G/pictures/exp1 MM.tif]

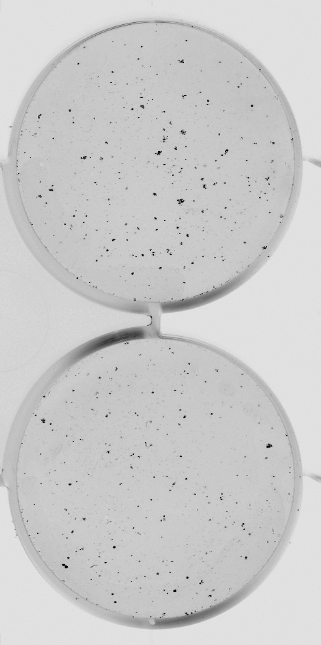

Supplement: Supplementary file 5 — Source data Fig. 4 [file 44321_2024_142_MOESM5_ESM.zip › Figure 4/G/pictures/exp3 SCDko#2.tif]

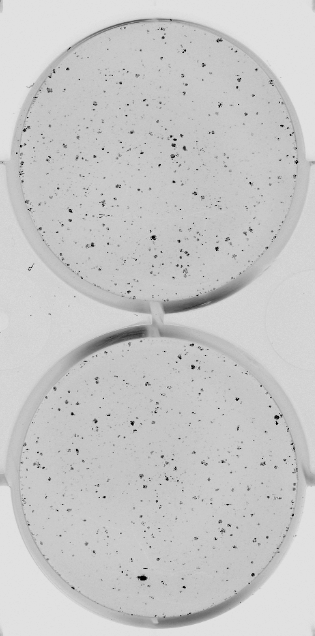

Supplement: Supplementary file 5 — Source data Fig. 4 [file 44321_2024_142_MOESM5_ESM.zip › Figure 4/G/pictures/exp3 SCDko#1.tif]

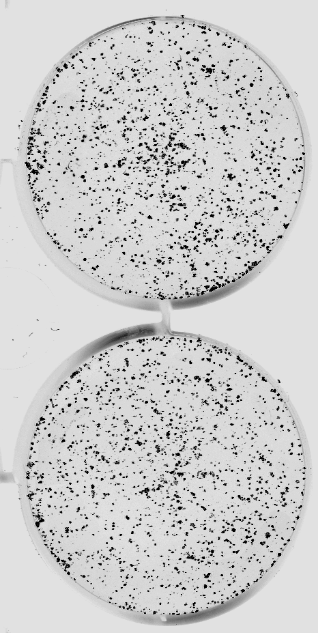

Supplement: Supplementary file 5 — Source data Fig. 4 [file 44321_2024_142_MOESM5_ESM.zip › Figure 4/G/pictures/exp1 NTC.tif]

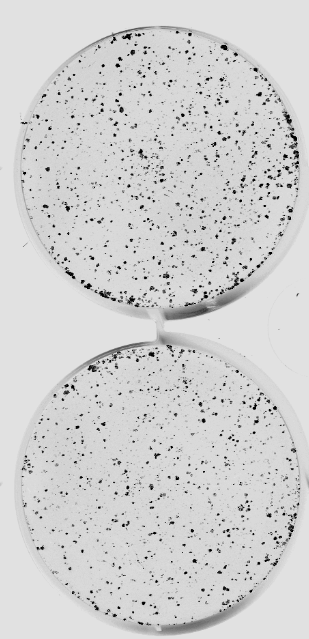

Supplement: Supplementary file 5 — Source data Fig. 4 [file 44321_2024_142_MOESM5_ESM.zip › Figure 4/G/pictures/exp1 SCDko#2.tif]

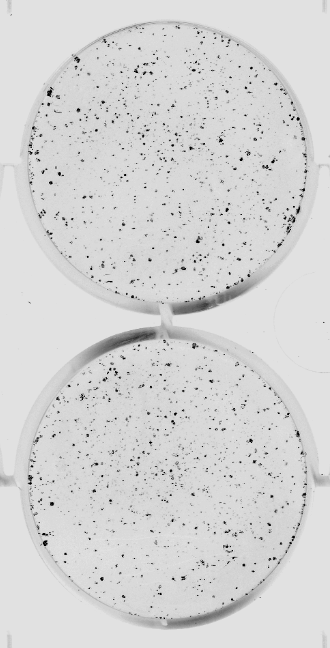

Supplement: Supplementary file 5 — Source data Fig. 4 [file 44321_2024_142_MOESM5_ESM.zip › Figure 4/G/pictures/exp1 SCDko#1.tif]

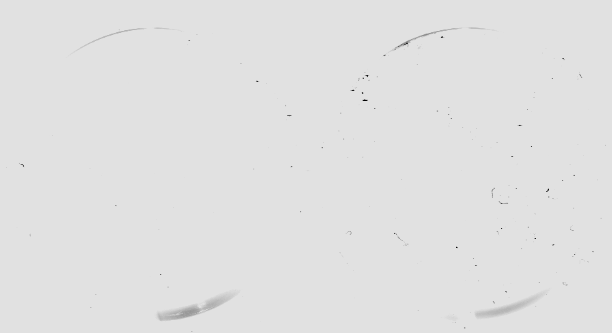

Supplement: Supplementary file 5 — Source data Fig. 4 [file 44321_2024_142_MOESM5_ESM.zip › Figure 4/G/pictures/exp2 MM.tif]

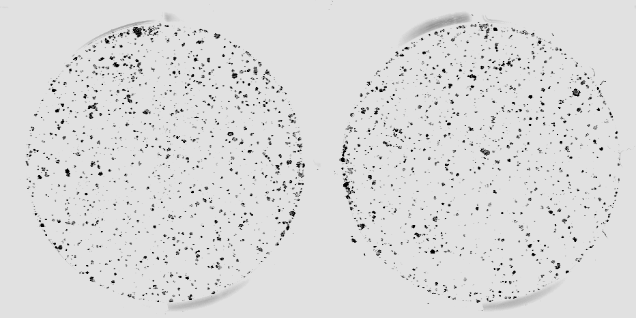

Supplement: Supplementary file 5 — Source data Fig. 4 [file 44321_2024_142_MOESM5_ESM.zip › Figure 4/G/pictures/exp2 NTC.tif]

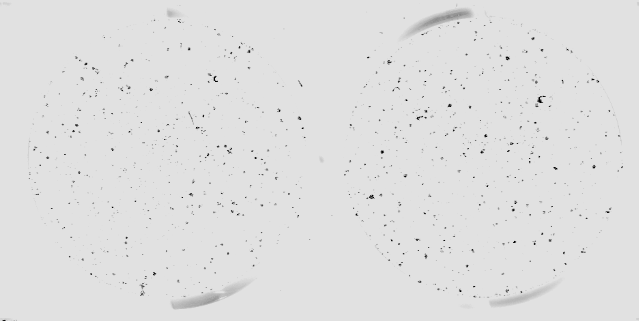

Supplement: Supplementary file 5 — Source data Fig. 4 [file 44321_2024_142_MOESM5_ESM.zip › Figure 4/G/pictures/exp2 SCDko#1.tif]

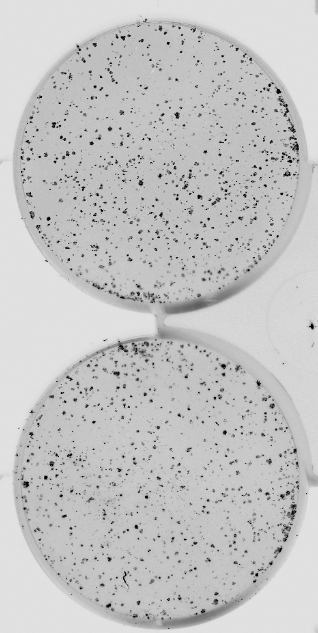

Supplement: Supplementary file 5 — Source data Fig. 4 [file 44321_2024_142_MOESM5_ESM.zip › Figure 4/G/pictures/exp3 NTC.tif]

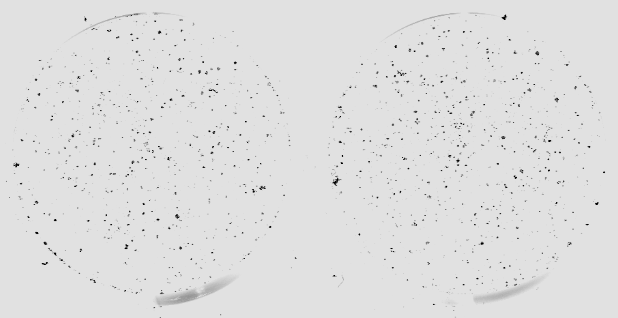

Supplement: Supplementary file 5 — Source data Fig. 4 [file 44321_2024_142_MOESM5_ESM.zip › Figure 4/G/pictures/exp2 SCDko#2.tif]

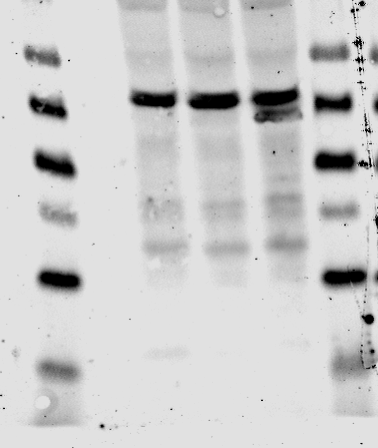

Supplement: Supplementary file 5 — Source data Fig. 4 [file 44321_2024_142_MOESM5_ESM.zip › Figure 4/E/western blot full scan/SCD#2 clone#2(C1) vs clone #1 (B9) vs NTC.tif]

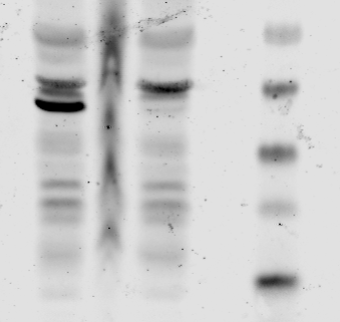

Supplement: Supplementary file 5 — Source data Fig. 4 [file 44321_2024_142_MOESM5_ESM.zip › Figure 4/E/western blot full scan/SCD#3 NTC vs clone #1 (B9) vs clone#2(C1).tif]
