# Supplementary material for: Breast cancer secretes anti-ferroptotic MUFAs and depends on selenoprotein synthesis for metastasis
Source: EMBO Mol Med. 2024 Oct 21;16(11):7. doi: 10.1038/s44321-024-00142-x (PMC11555046; doi:10.1038/s44321-024-00142-x)
Supplement: Supplementary file 3 — Source data Fig. 2 [file 44321_2024_142_MOESM3_ESM.zip › Figure 2/F/western images.pptx]

## Slide 1
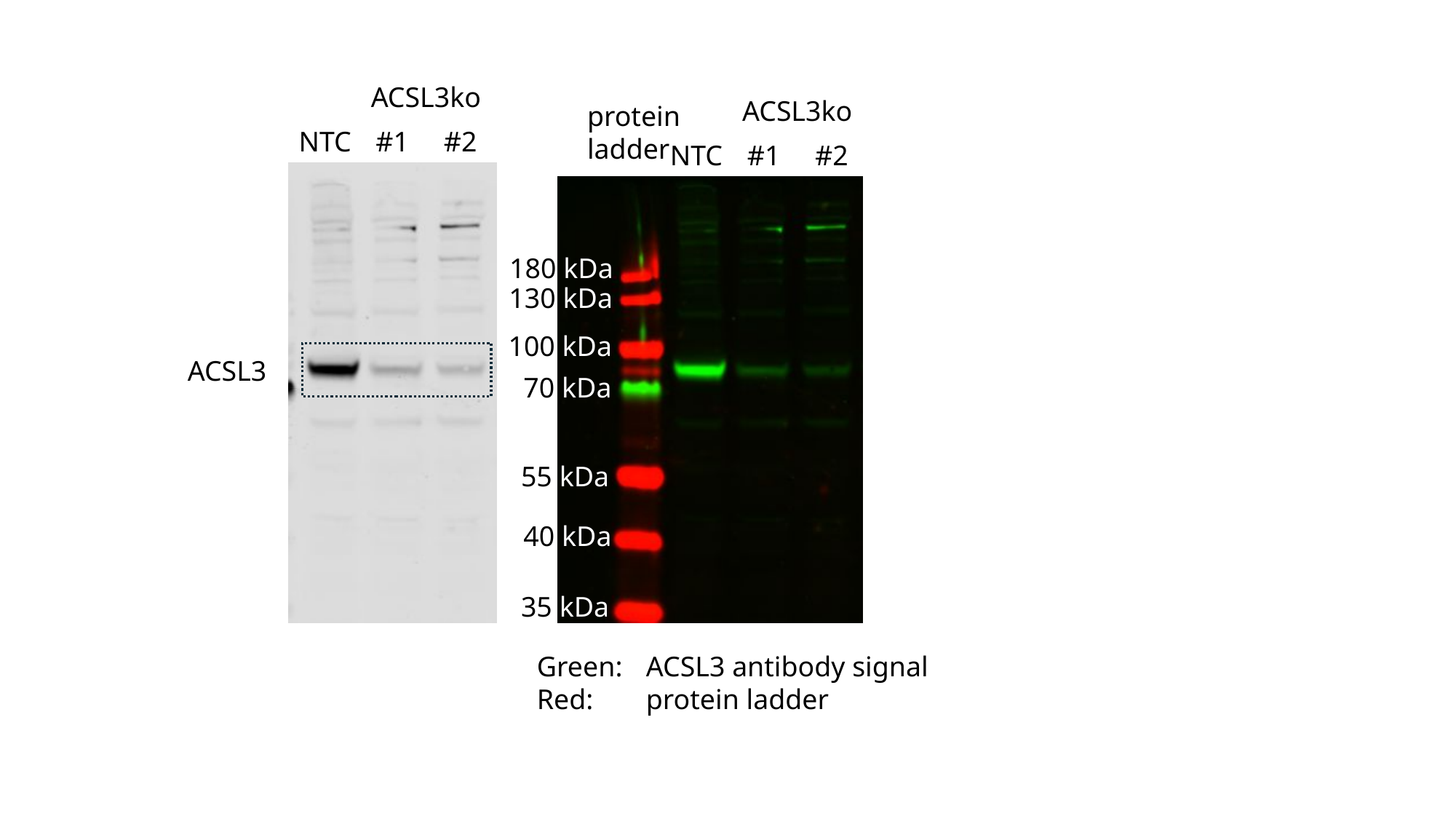

ACSL3ko
ACSL3ko
protein
ladder
NTC
#1
#2
NTC
#1
#2
180 kDa
130 kDa
100 kDa
ACSL3
70 kDa
55 kDa
40 kDa
35 kDa
Green: 	ACSL3 antibody signal
Red: 	protein ladder

## Slide 2
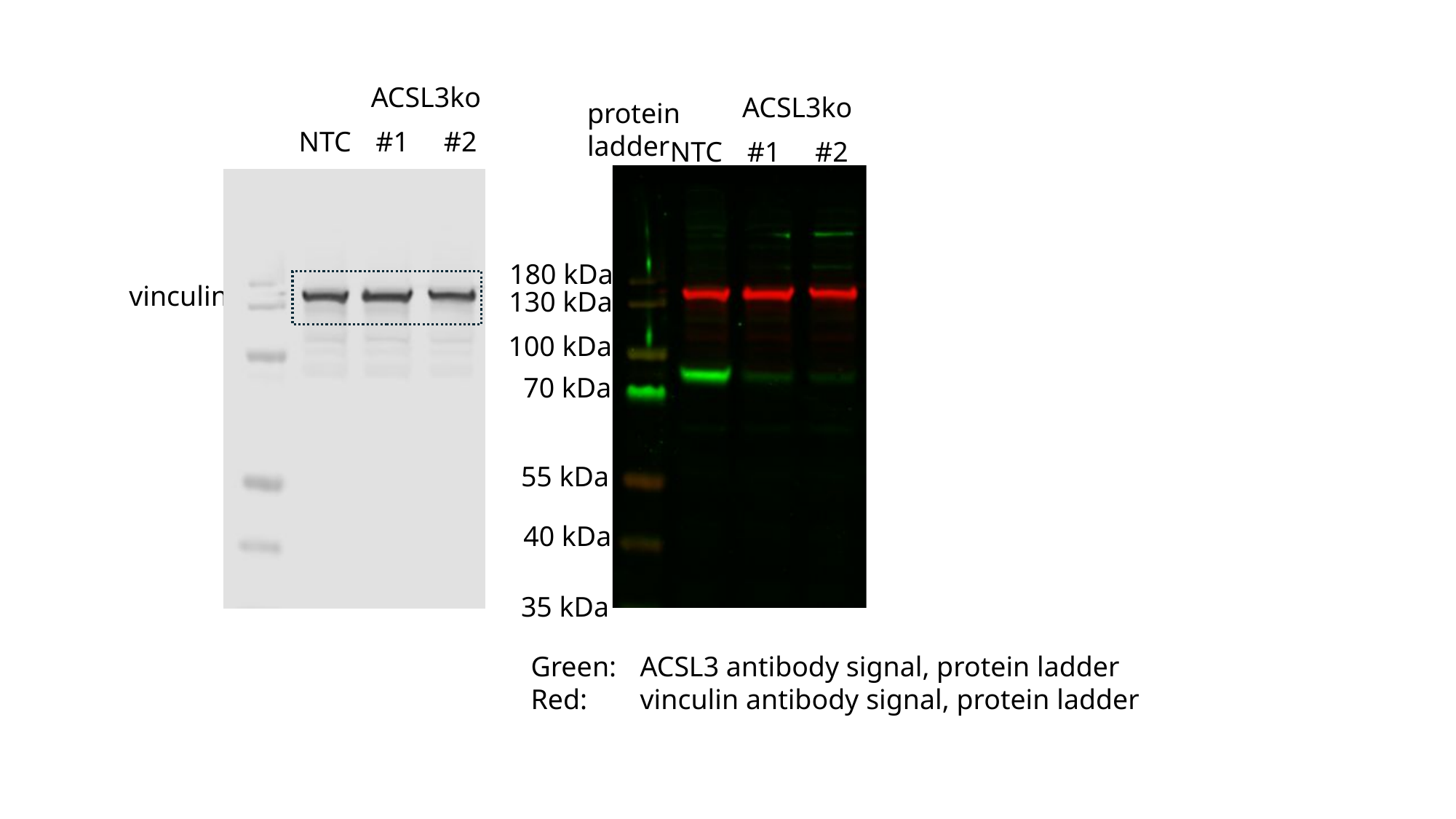

ACSL3ko
ACSL3ko
protein
ladder
NTC
#1
#2
NTC
#1
#2
180 kDa
vinculin
130 kDa
100 kDa
70 kDa
55 kDa
40 kDa
35 kDa
Green: 	ACSL3 antibody signal, protein ladder
Red: 	vinculin antibody signal, protein ladder
