# Supplementary material for: Breast cancer secretes anti-ferroptotic MUFAs and depends on selenoprotein synthesis for metastasis
Source: EMBO Mol Med. 2024 Oct 21;16(11):7. doi: 10.1038/s44321-024-00142-x (PMC11555046; doi:10.1038/s44321-024-00142-x)
Supplement: Supplementary file 3 — Source data Fig. 2 [file 44321_2024_142_MOESM3_ESM.zip › Figure 2/C/pictures and labels.pptx]

## Slide 1
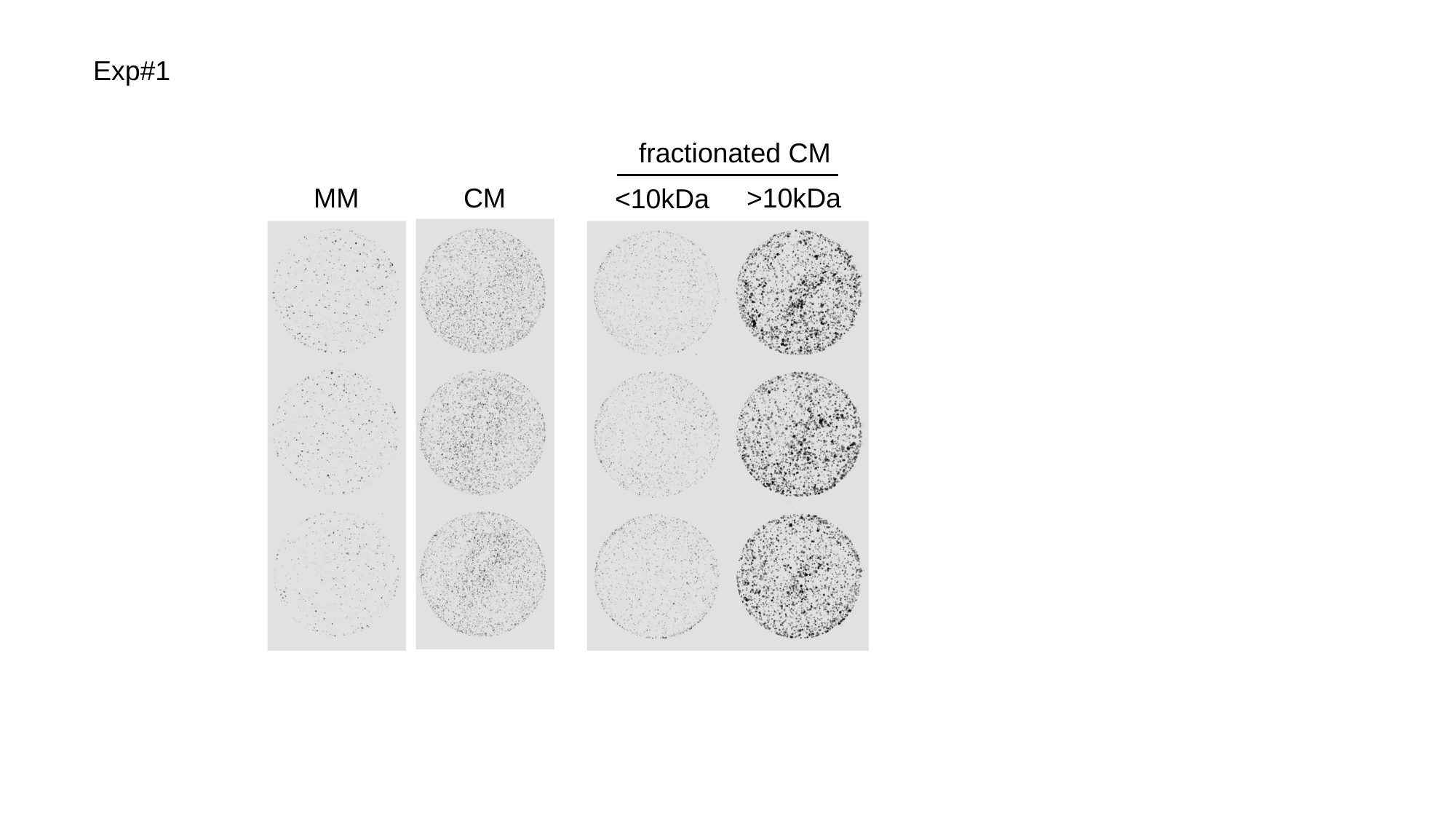

Exp#1
fractionated CM
CM
>10kDa
MM
<10kDa

## Slide 2
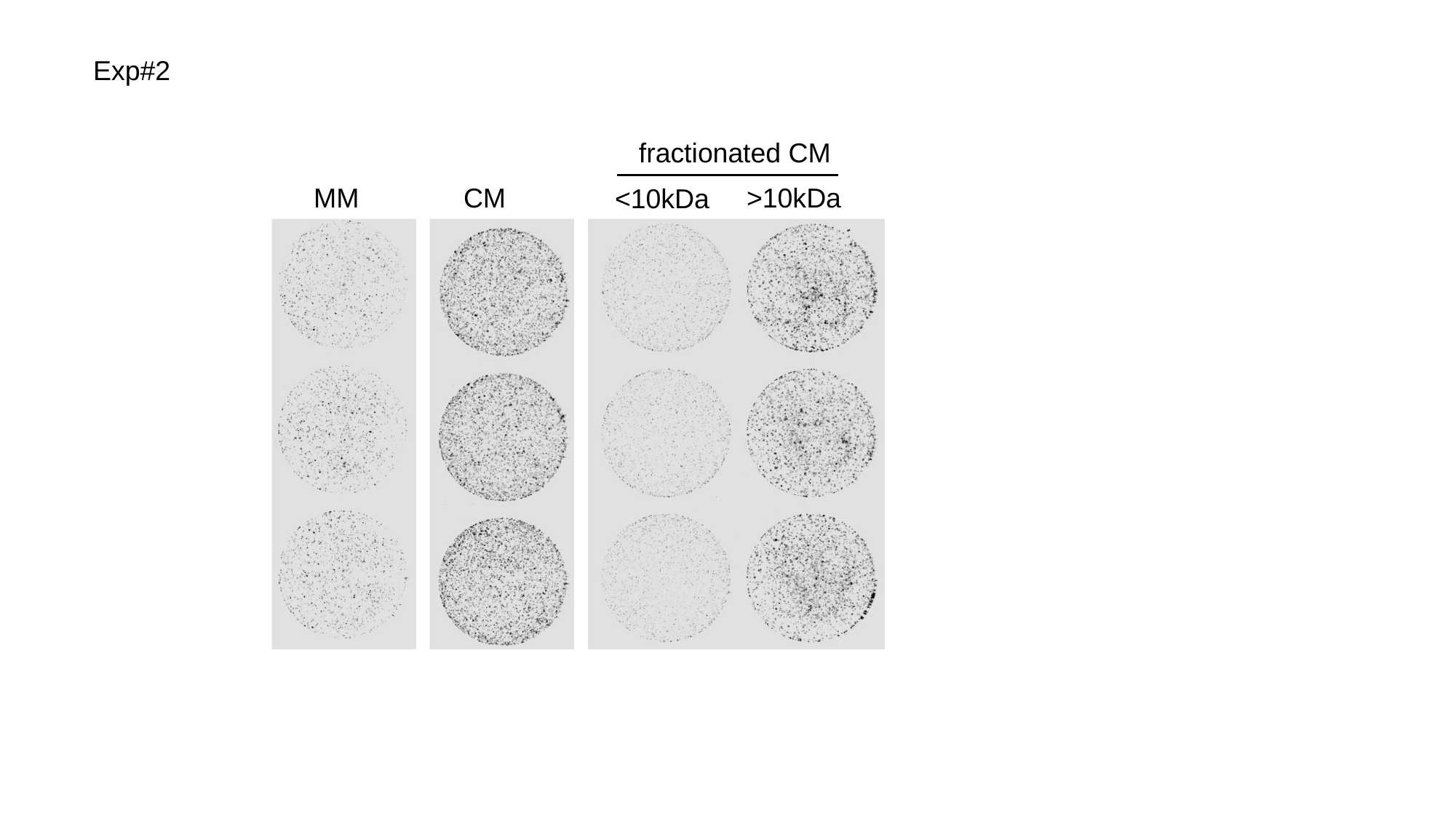

Exp#2
fractionated CM
CM
>10kDa
MM
<10kDa

## Slide 3
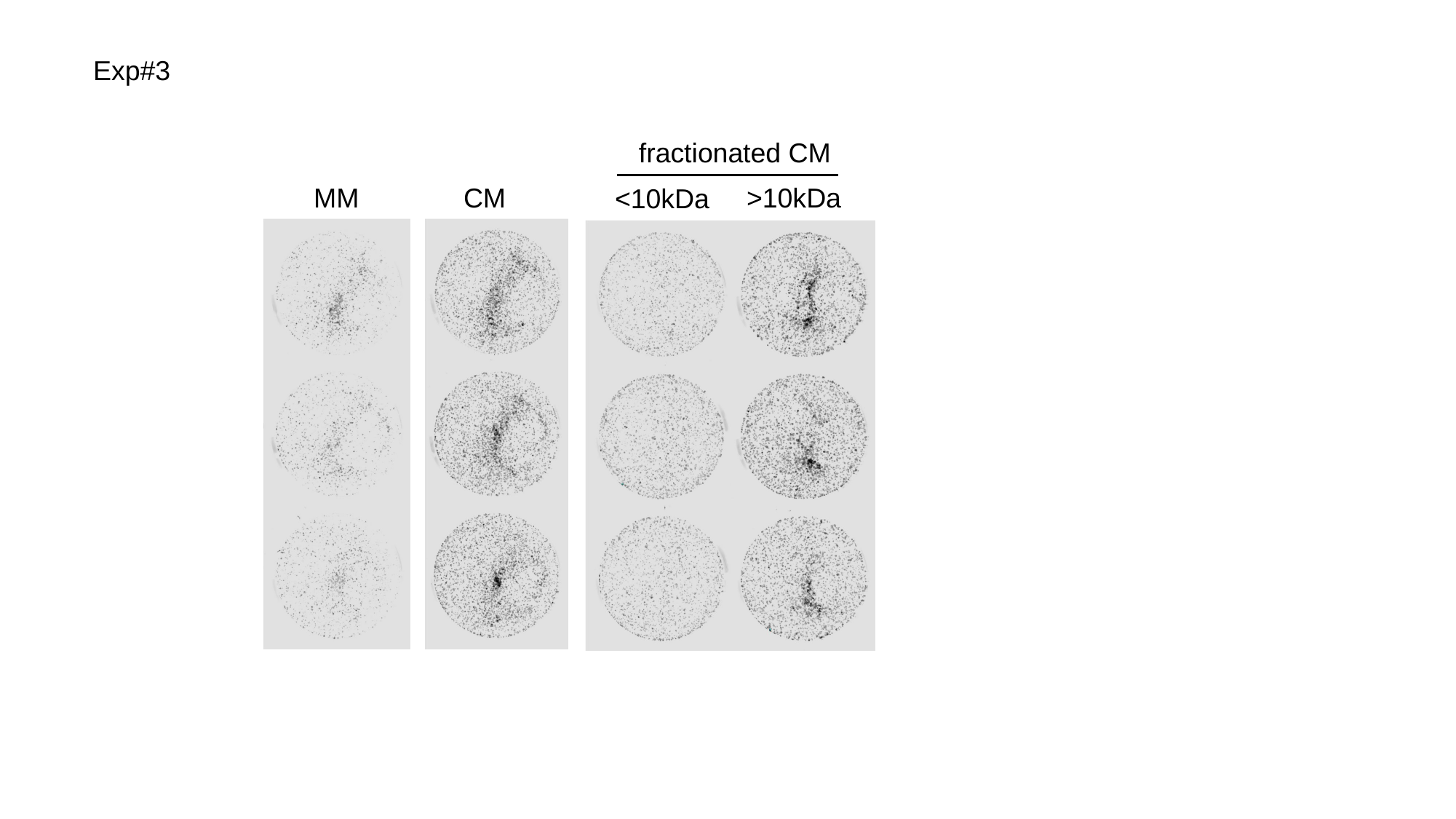

Exp#3
fractionated CM
CM
>10kDa
MM
<10kDa
