# Supplementary material for: Breast cancer secretes anti-ferroptotic MUFAs and depends on selenoprotein synthesis for metastasis
Source: EMBO Mol Med. 2024 Oct 21;16(11):7. doi: 10.1038/s44321-024-00142-x (PMC11555046; doi:10.1038/s44321-024-00142-x)
Supplement: Supplementary file 5 — Source data Fig. 4 [file 44321_2024_142_MOESM5_ESM.zip › Figure 4/G/pictures and labels.pptx]

## Slide 1
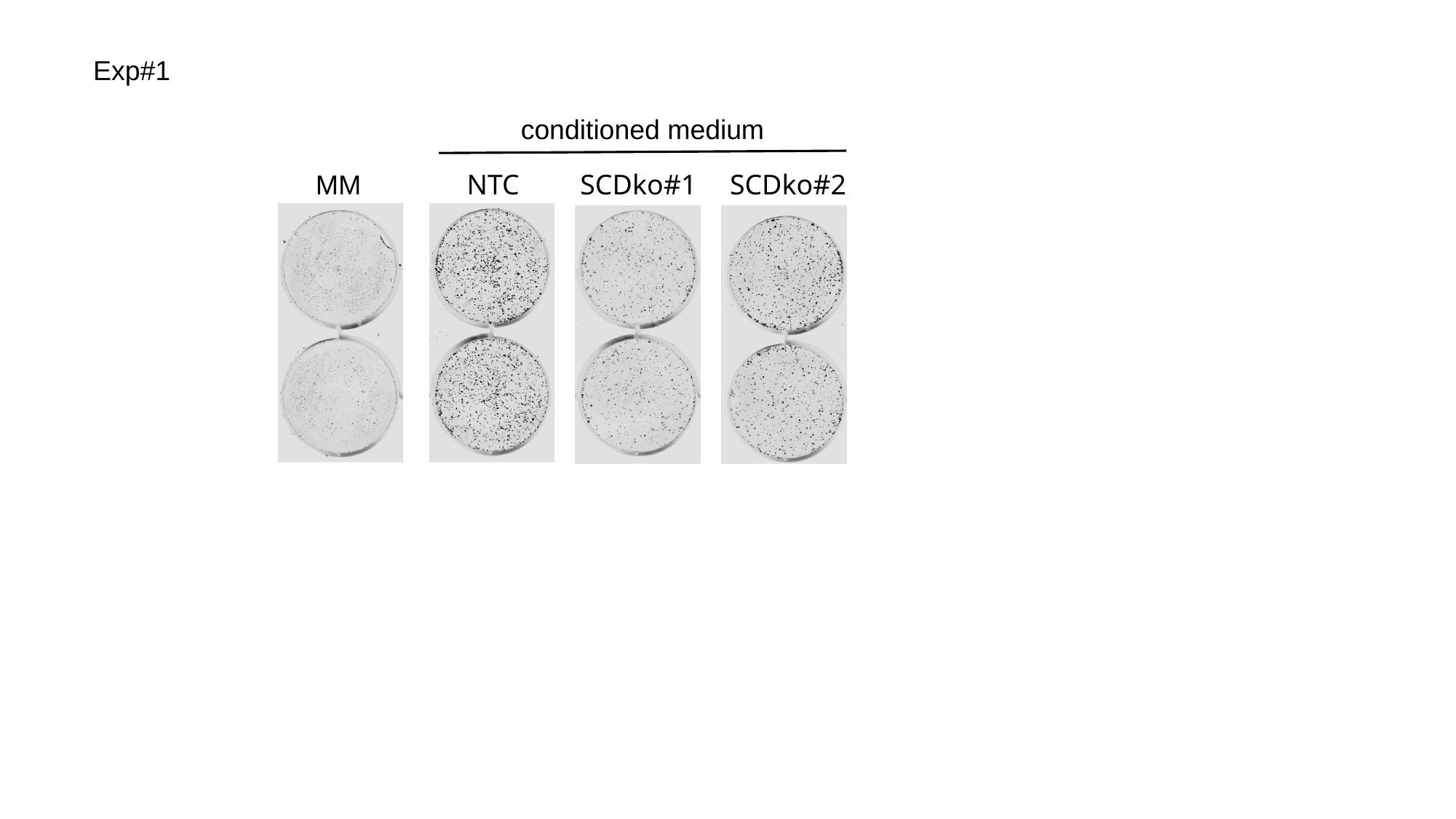

Exp#1
conditioned medium
MM
NTC
SCDko#1
SCDko#2

## Slide 2
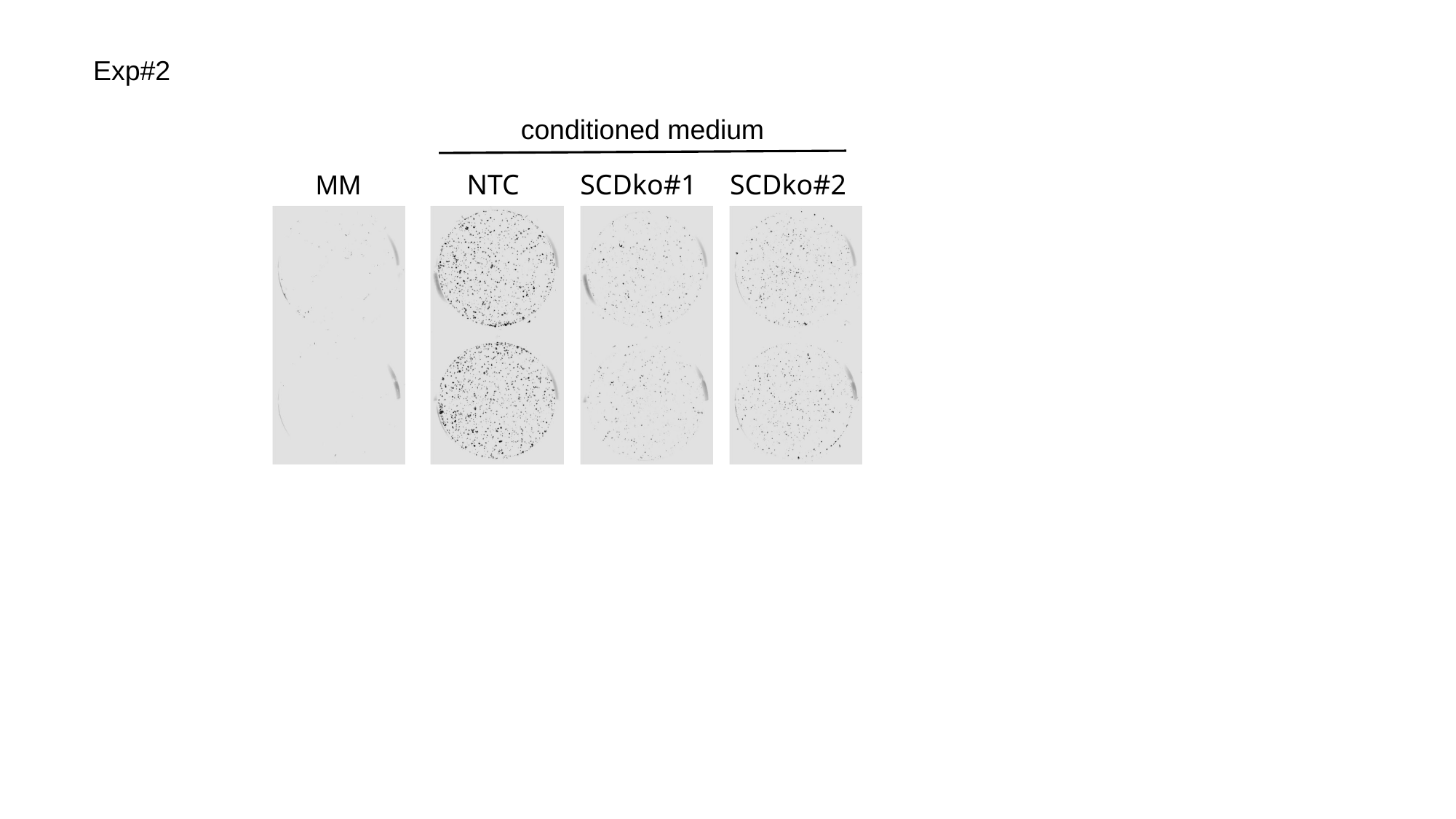

Exp#2
conditioned medium
MM
NTC
SCDko#1
SCDko#2

## Slide 3
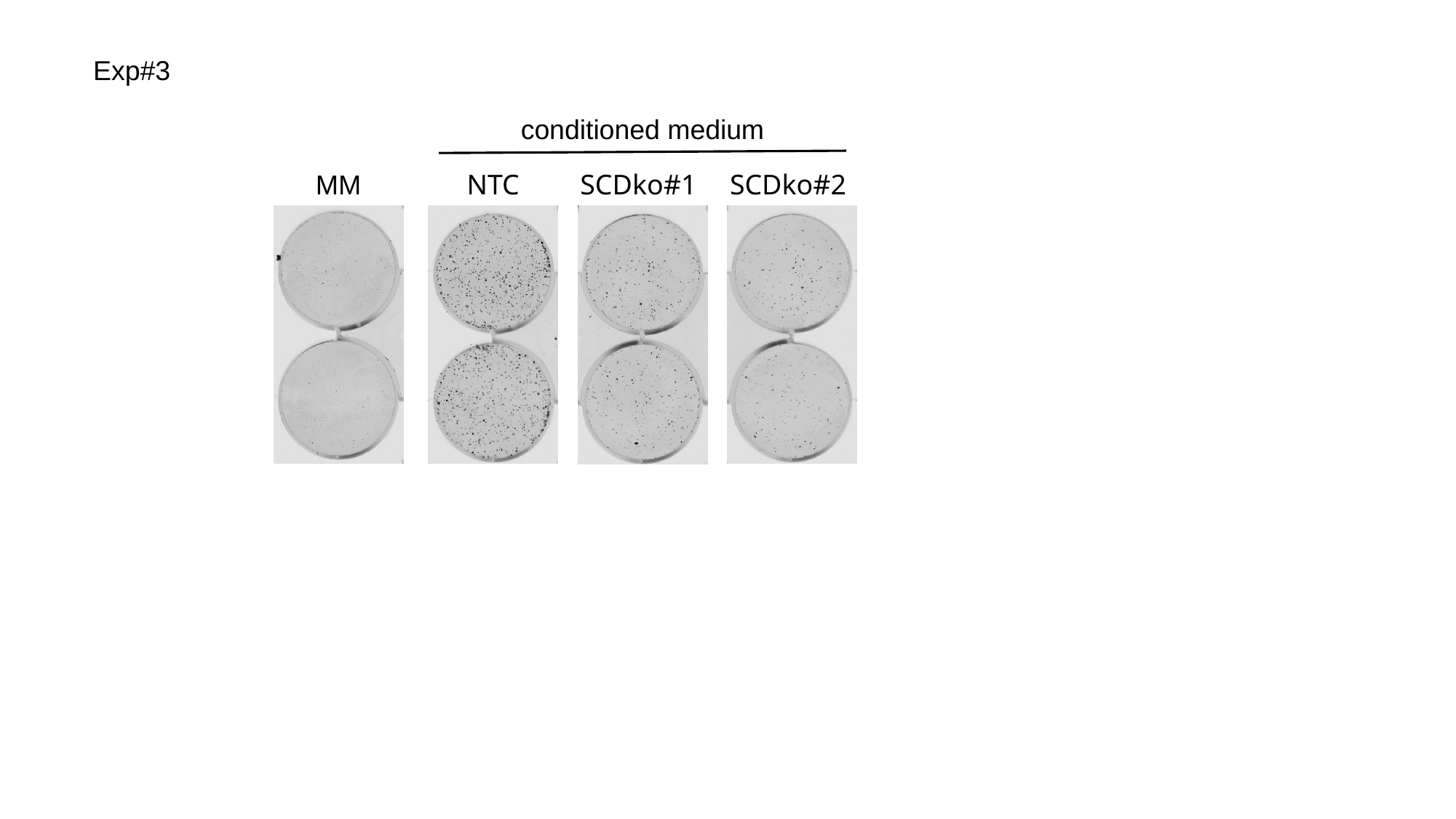

Exp#3
conditioned medium
MM
NTC
SCDko#1
SCDko#2
