# Supplementary material for: Breast cancer secretes anti-ferroptotic MUFAs and depends on selenoprotein synthesis for metastasis
Source: EMBO Mol Med. 2024 Oct 21;16(11):7. doi: 10.1038/s44321-024-00142-x (PMC11555046; doi:10.1038/s44321-024-00142-x)
Supplement: Supplementary file 5 — Source data Fig. 4 [file 44321_2024_142_MOESM5_ESM.zip › Figure 4/H/full scan and labels.pptx]

## Slide 1
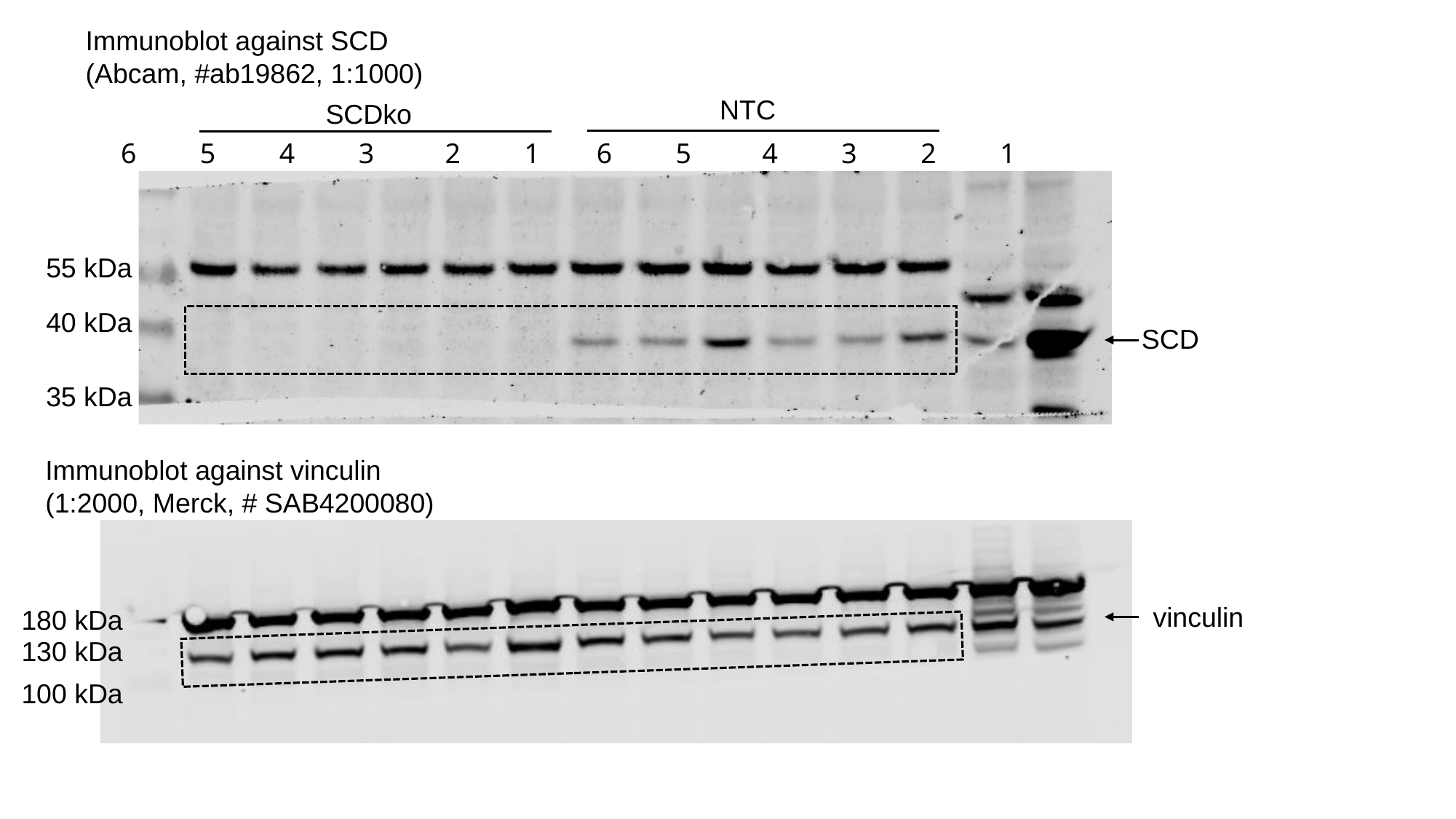

Immunoblot against SCD
(Abcam, #ab19862, 1:1000)
NTC
SCDko
6 5 4 3 2 1 6 5 4 3 2 1
55 kDa
40 kDa
SCD
35 kDa
Immunoblot against vinculin
(1:2000, Merck, # SAB4200080)
vinculin
180 kDa
130 kDa
100 kDa
