# Supplementary material for: Breast cancer secretes anti-ferroptotic MUFAs and depends on selenoprotein synthesis for metastasis
Source: EMBO Mol Med. 2024 Oct 21;16(11):7. doi: 10.1038/s44321-024-00142-x (PMC11555046; doi:10.1038/s44321-024-00142-x)
Supplement: Supplementary file 5 — Source data Fig. 4 [file 44321_2024_142_MOESM5_ESM.zip › Figure 4/B/pictures and labels.pptx]

## Slide 1
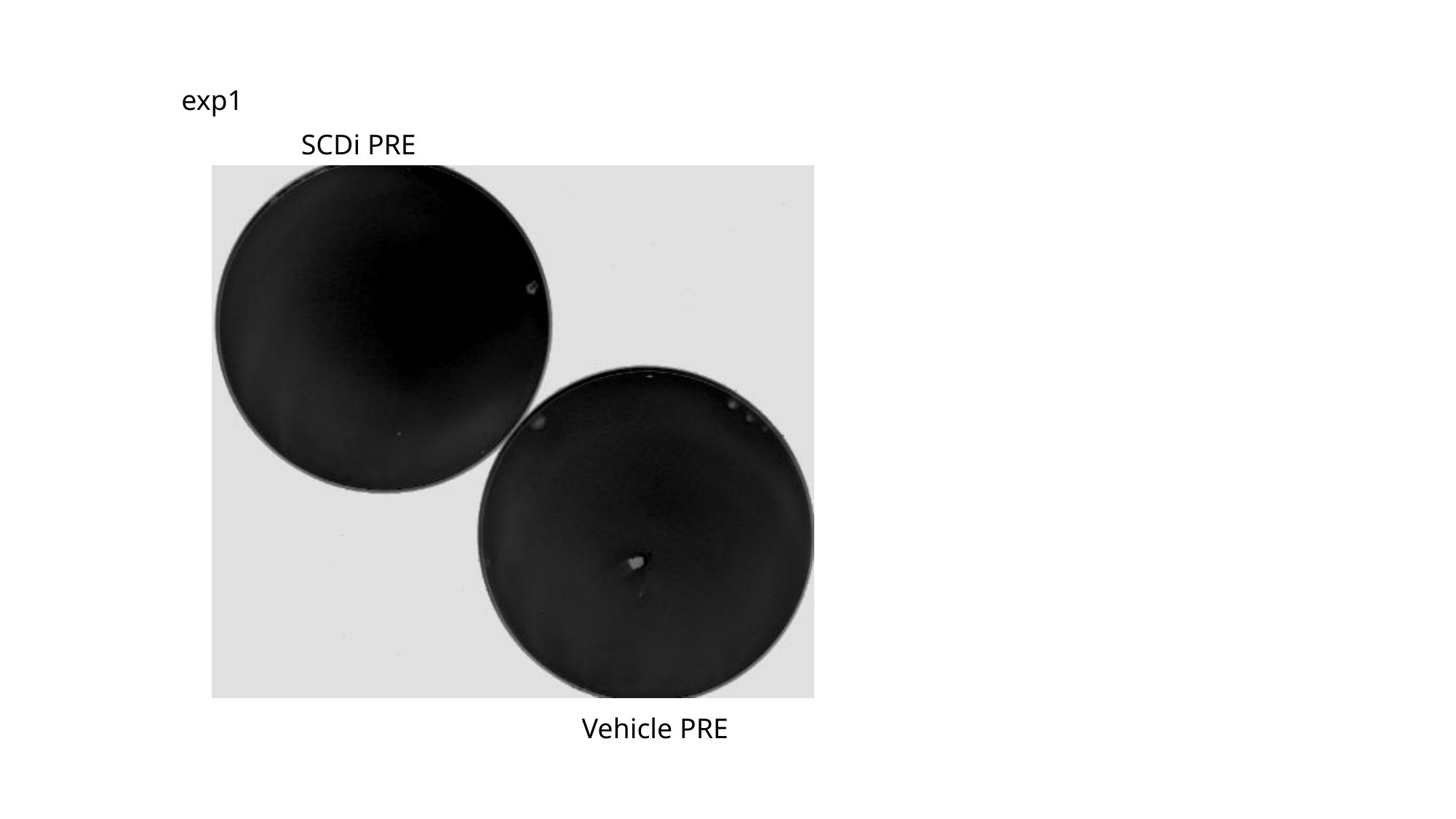

exp1
SCDi PRE
Vehicle PRE

## Slide 2
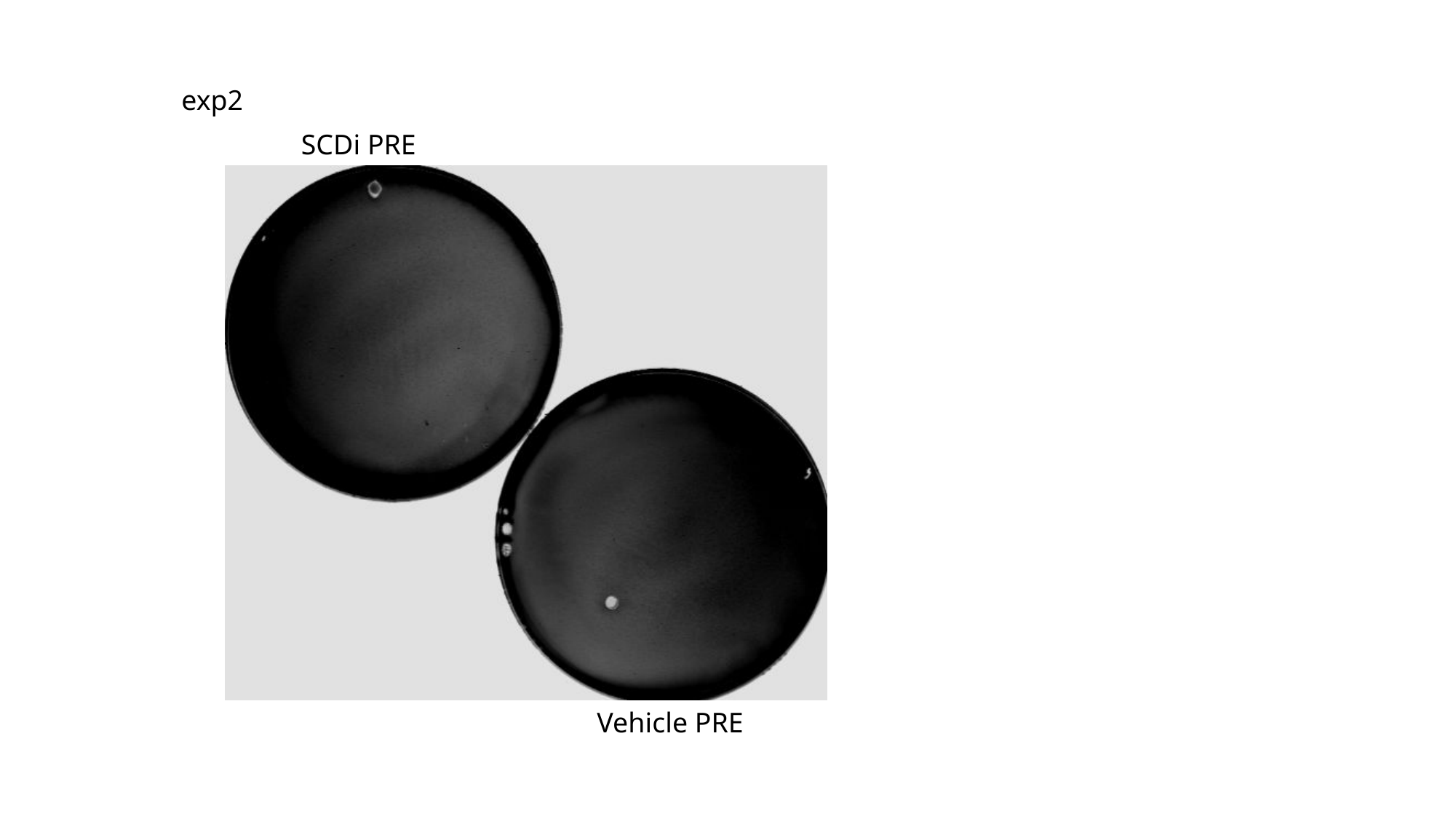

exp2
SCDi PRE
Vehicle PRE

## Slide 3
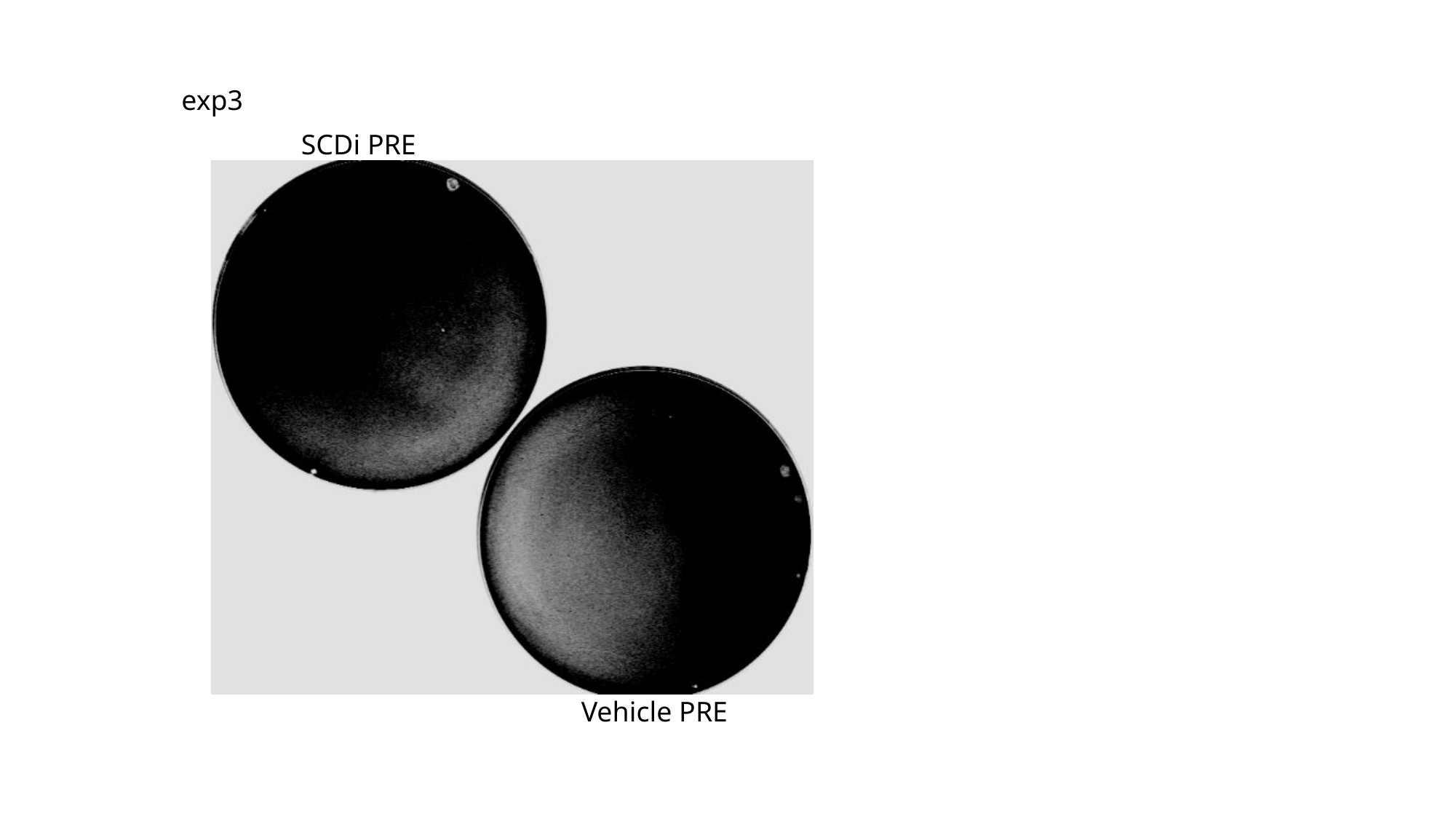

exp3
SCDi PRE
Vehicle PRE

## Slide 4
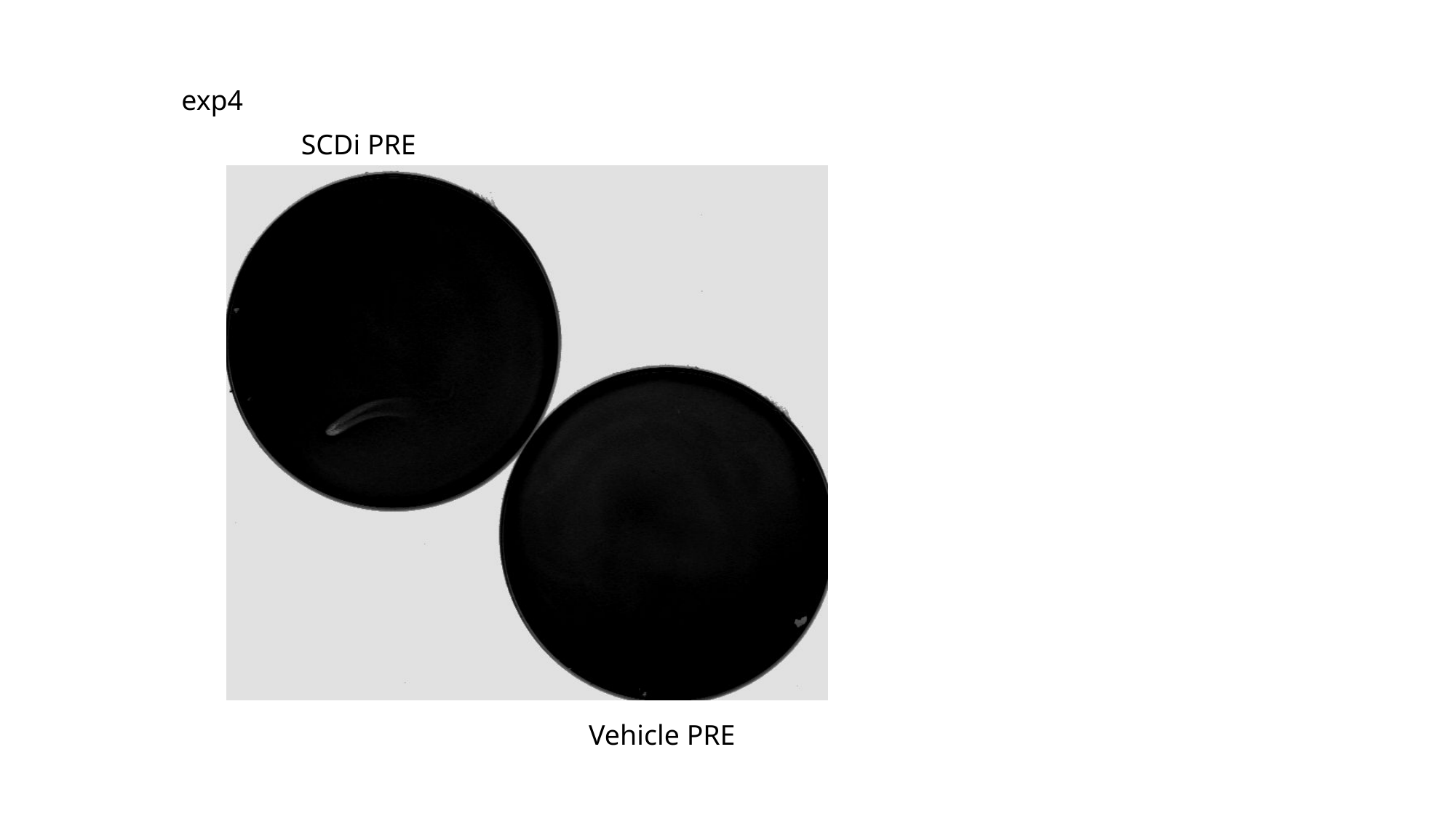

exp4
SCDi PRE
Vehicle PRE
