# Supplementary figures and images for: Breast cancer secretes anti-ferroptotic MUFAs and depends on selenoprotein synthesis for metastasis (part 2 of 2)
Source: EMBO Mol Med. 2024 Oct 21;16(11):7. doi: 10.1038/s44321-024-00142-x (PMC11555046; doi:10.1038/s44321-024-00142-x)

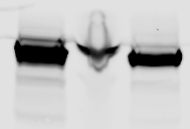

Supplement: Supplementary file 5 — Source data Fig. 4 [file 44321_2024_142_MOESM5_ESM.zip › Figure 4/E/western blot full scan/vinculin#3 NTC vs clone #1 (B9) vs clone#2(C1).tif]

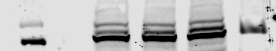

Supplement: Supplementary file 5 — Source data Fig. 4 [file 44321_2024_142_MOESM5_ESM.zip › Figure 4/E/western blot full scan/vinculin#2 clone#2(C1) vs clone #1 (B9) vs NTC.tif]

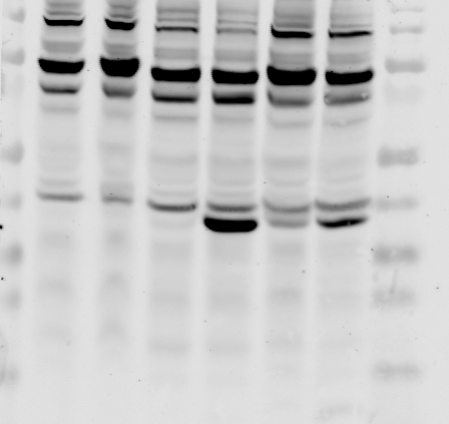

Supplement: Supplementary file 5 — Source data Fig. 4 [file 44321_2024_142_MOESM5_ESM.zip › Figure 4/E/western blot full scan/4E SCD clone C10 (not used) vs clone#2(C1) vs clone #1 (B9) vs NTC vs EV vs SCDoe.tif]

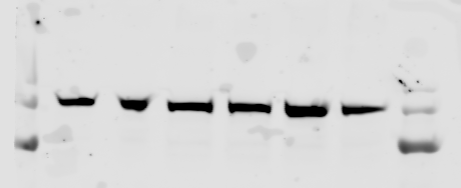

Supplement: Supplementary file 5 — Source data Fig. 4 [file 44321_2024_142_MOESM5_ESM.zip › Figure 4/E/western blot full scan/4E vinculin clone C10 (not used) vs clone#2(C1) vs clone #1 (B9) vs NTC vs EV vs SCDoe.tif]

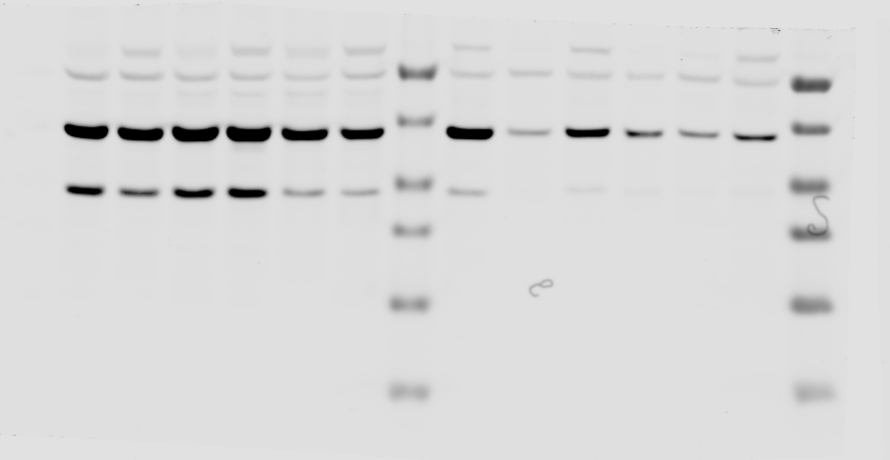

Supplement: Supplementary file 6 — Source data Fig. 5 [file 44321_2024_142_MOESM6_ESM.zip › Figure 5/G/SCD MCF7 and BT549.tif]

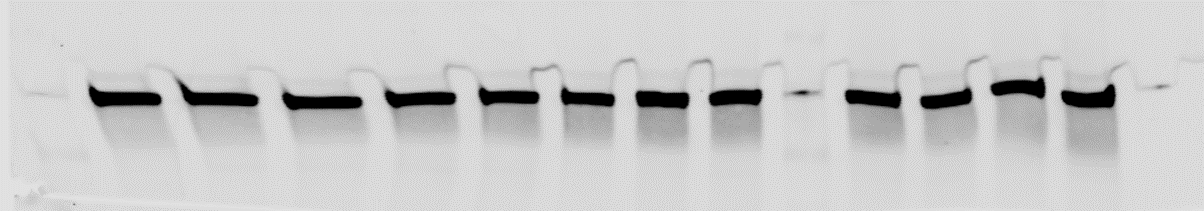

Supplement: Supplementary file 6 — Source data Fig. 5 [file 44321_2024_142_MOESM6_ESM.zip › Figure 5/G/vinculin MDA468.tif]

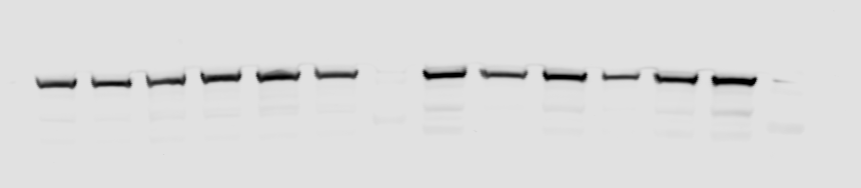

Supplement: Supplementary file 6 — Source data Fig. 5 [file 44321_2024_142_MOESM6_ESM.zip › Figure 5/G/vinculin MCF7 and BT549.tif]

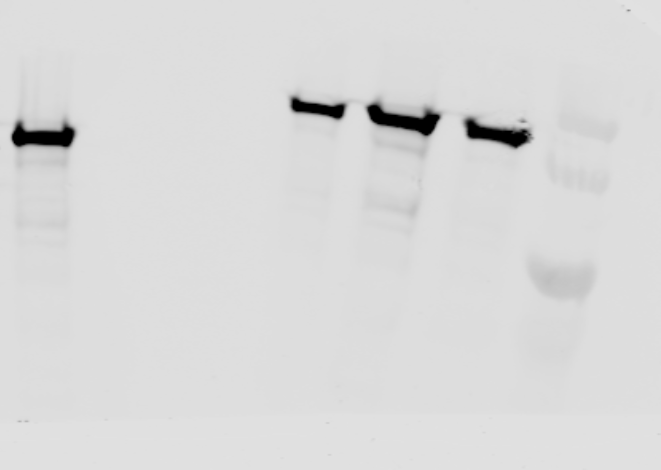

Supplement: Supplementary file 6 — Source data Fig. 5 [file 44321_2024_142_MOESM6_ESM.zip › Figure 5/G/vinculin bt549 exp 4 and 5.tif]

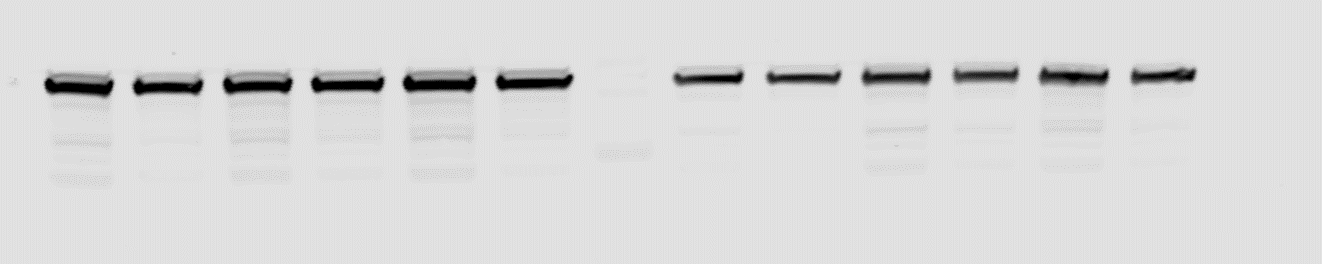

Supplement: Supplementary file 6 — Source data Fig. 5 [file 44321_2024_142_MOESM6_ESM.zip › Figure 5/G/vinculin MDA231 and CAL120.tif]

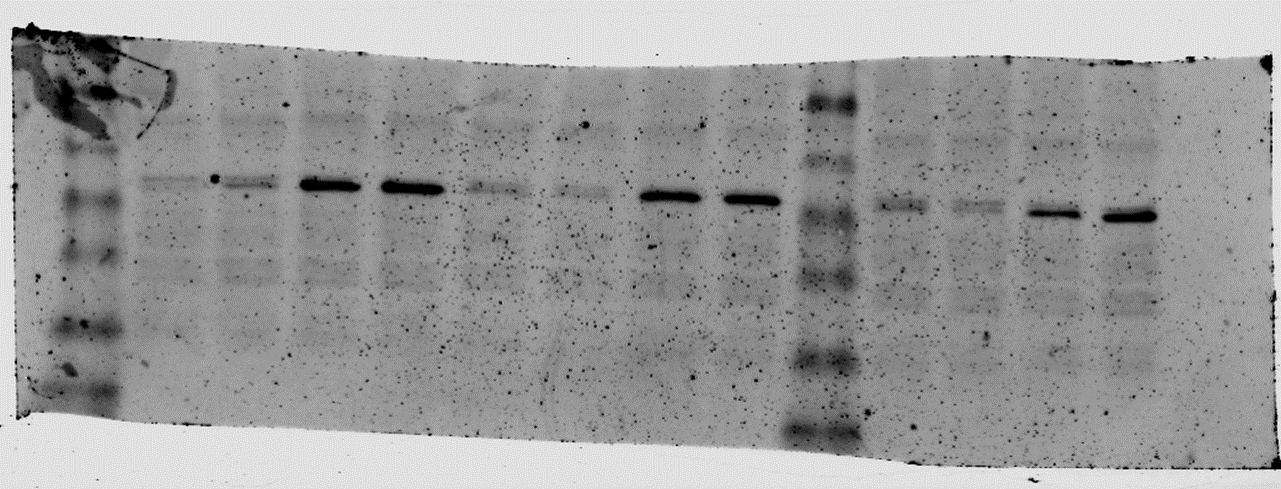

Supplement: Supplementary file 6 — Source data Fig. 5 [file 44321_2024_142_MOESM6_ESM.zip › Figure 5/G/SCD MDA468.tif]

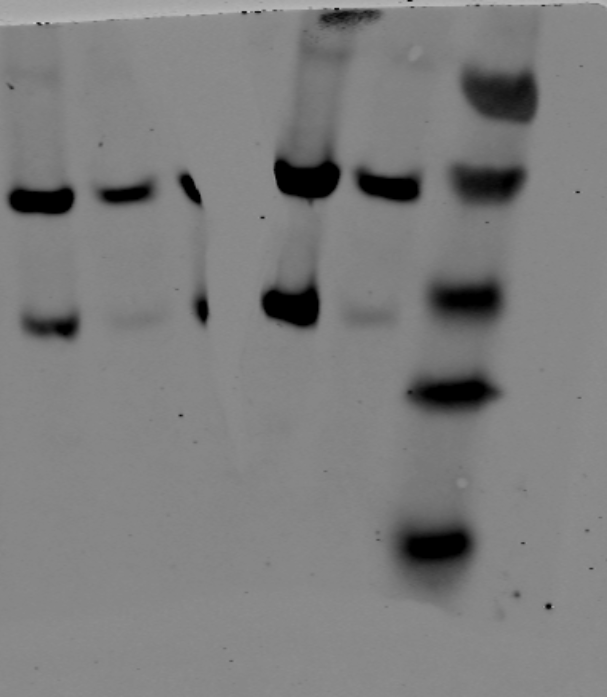

Supplement: Supplementary file 6 — Source data Fig. 5 [file 44321_2024_142_MOESM6_ESM.zip › Figure 5/G/SCD bt549 exp4 and 5.tif]

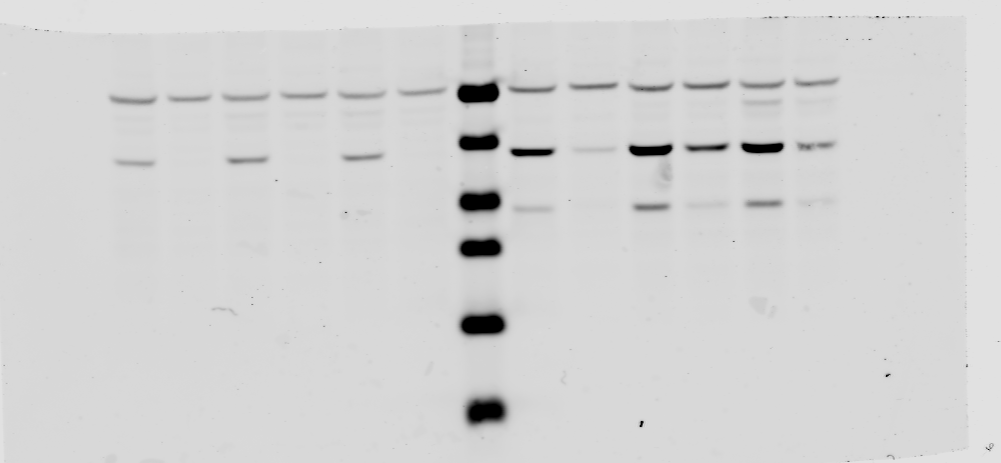

Supplement: Supplementary file 6 — Source data Fig. 5 [file 44321_2024_142_MOESM6_ESM.zip › Figure 5/G/SCD MDA231 and CAl120.tif]

## Slide 1
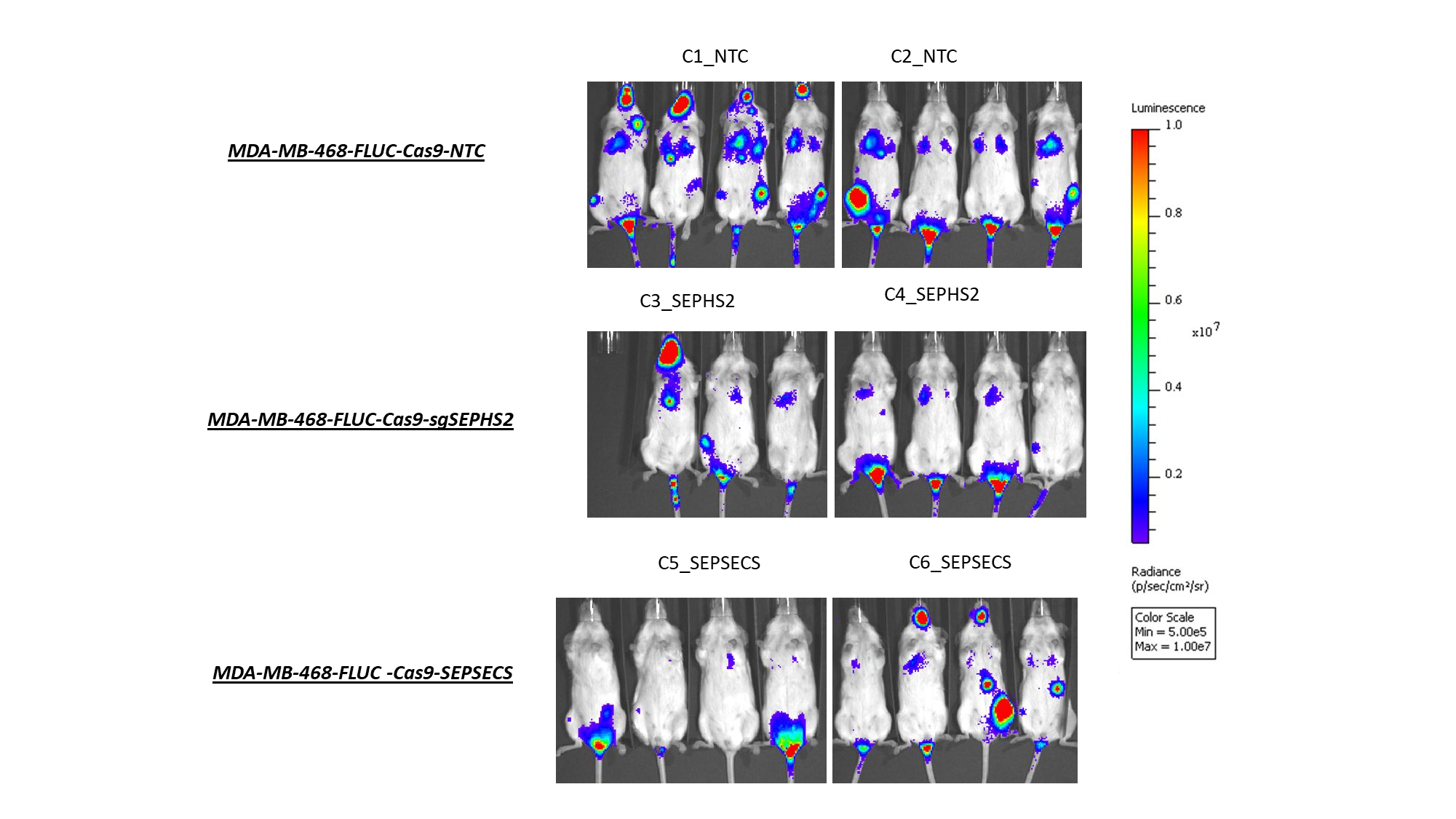

Supplement: Supplementary file 7 — Source data Fig. 6 [file 44321_2024_142_MOESM7_ESM.zip › Figure 6/I/images.pptx]

## Slide 1
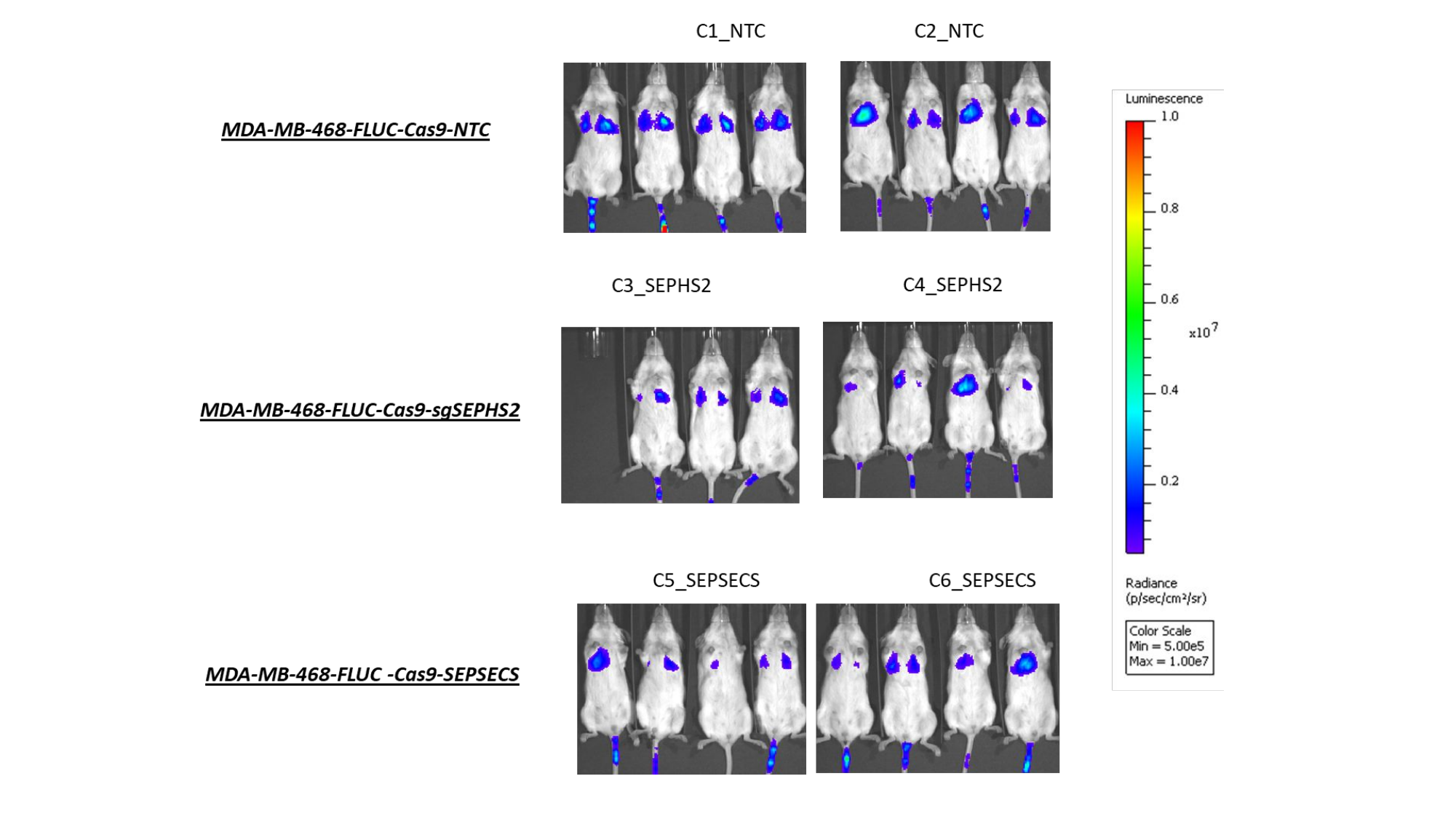

Supplement: Supplementary file 7 — Source data Fig. 6 [file 44321_2024_142_MOESM7_ESM.zip › Figure 6/H/images.pptx]

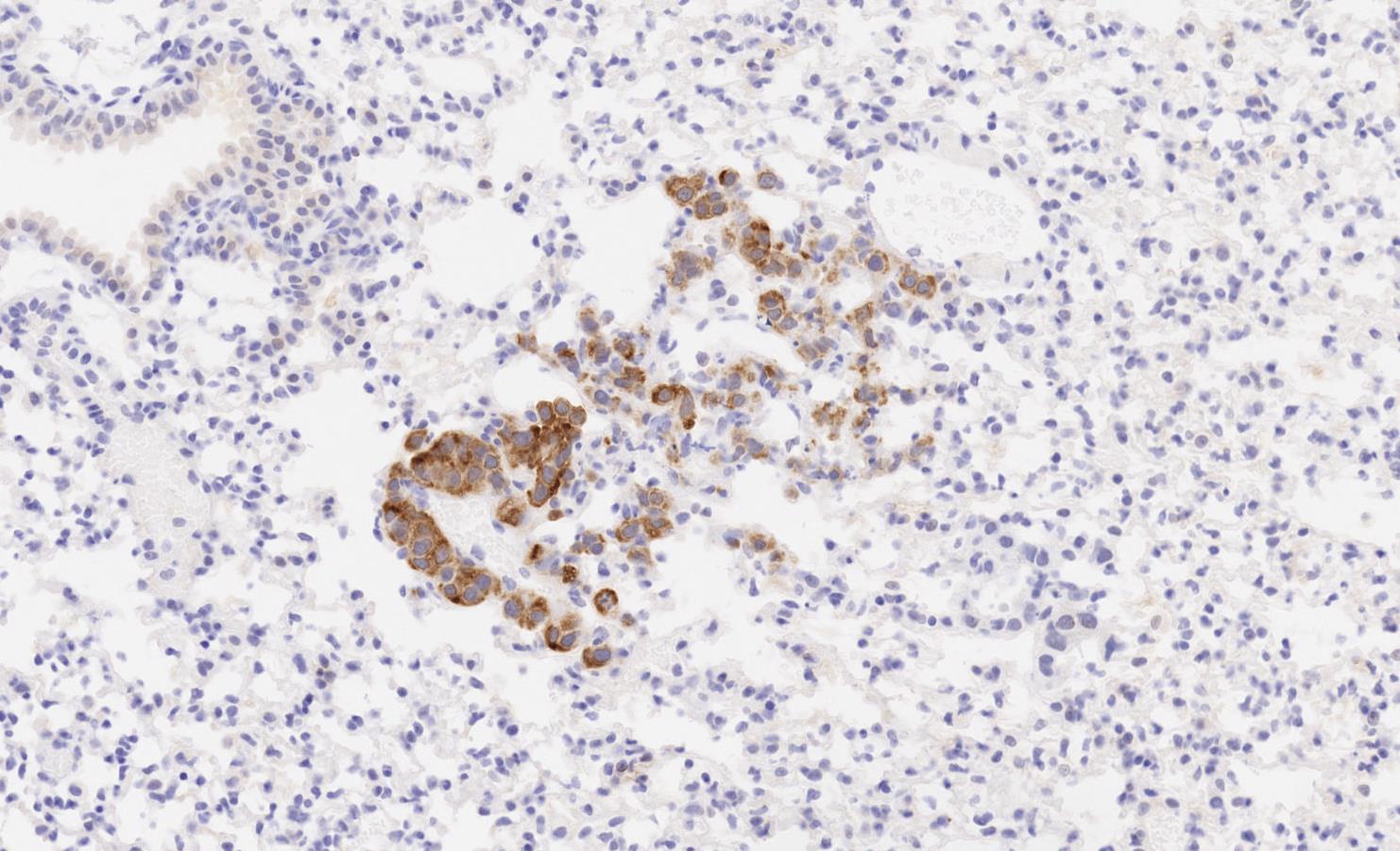

Supplement: Supplementary file 7 — Source data Fig. 6 [file 44321_2024_142_MOESM7_ESM.zip › Figure 6/J/NTC 90 2d s13 cas9 20x.jpg]

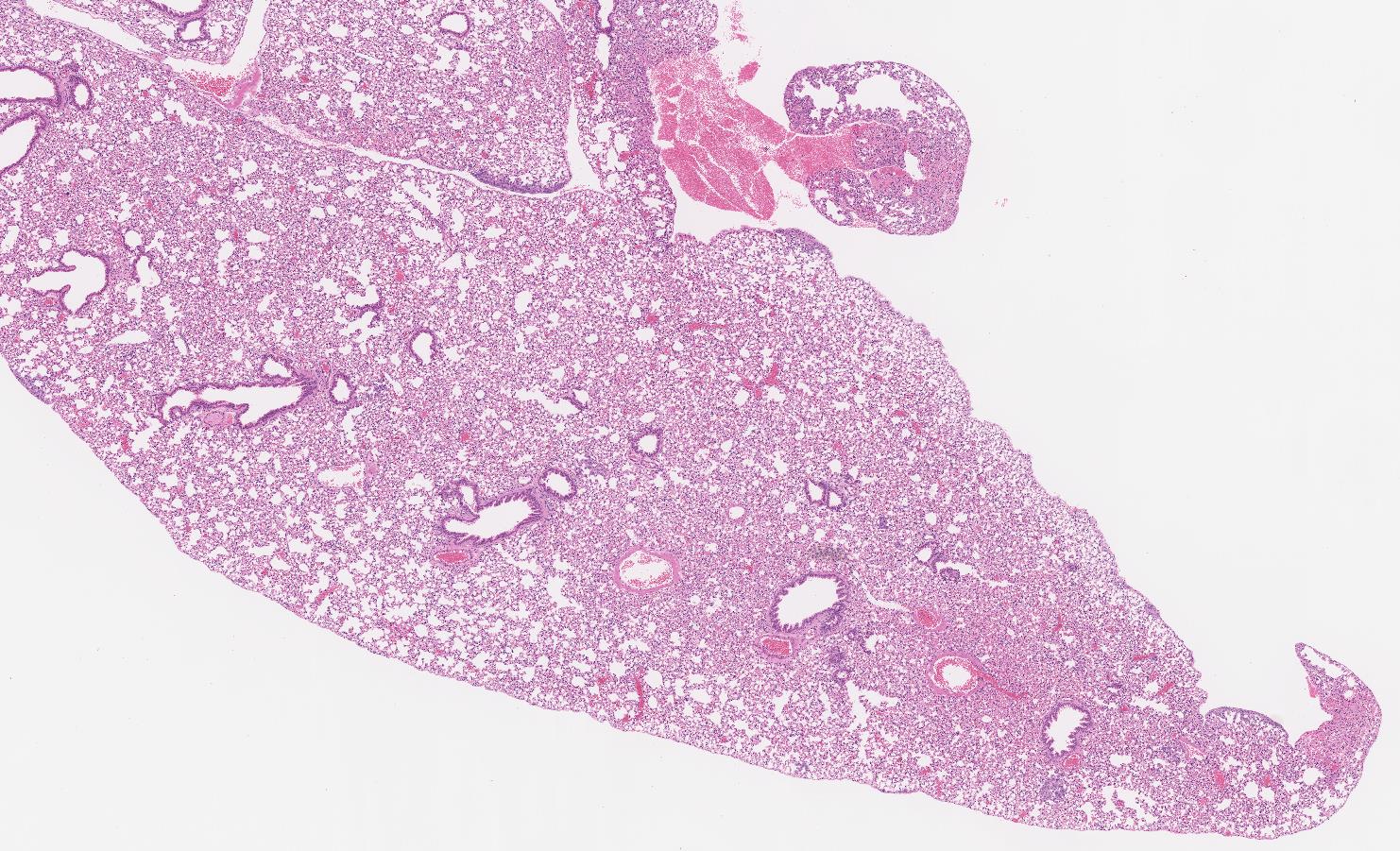

Supplement: Supplementary file 7 — Source data Fig. 6 [file 44321_2024_142_MOESM7_ESM.zip › Figure 6/J/sgSEPHS2 90 4b s11 HE 2x.jpg]

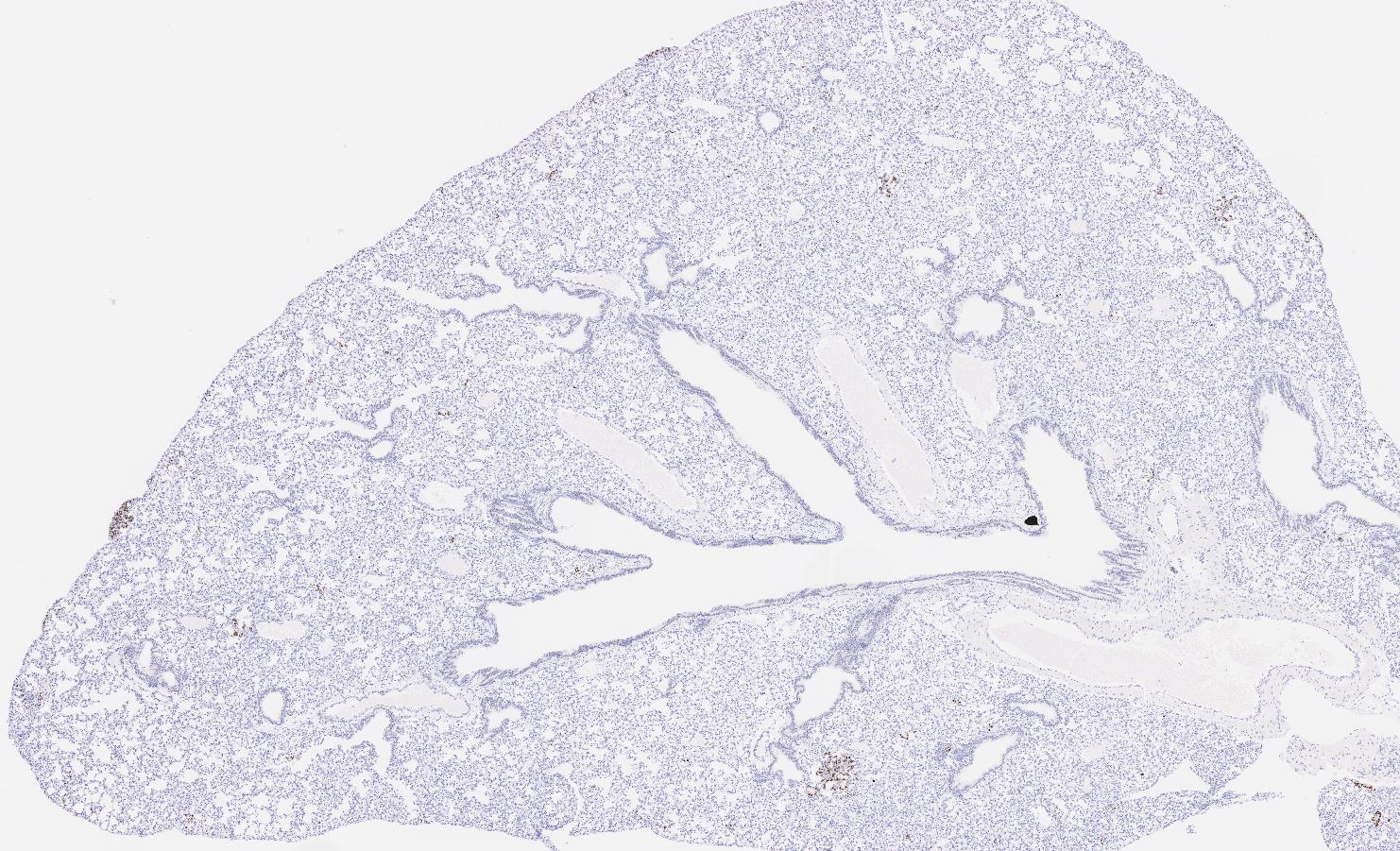

Supplement: Supplementary file 7 — Source data Fig. 6 [file 44321_2024_142_MOESM7_ESM.zip › Figure 6/J/NTC 90 2d s13 ku80 2x.jpg]

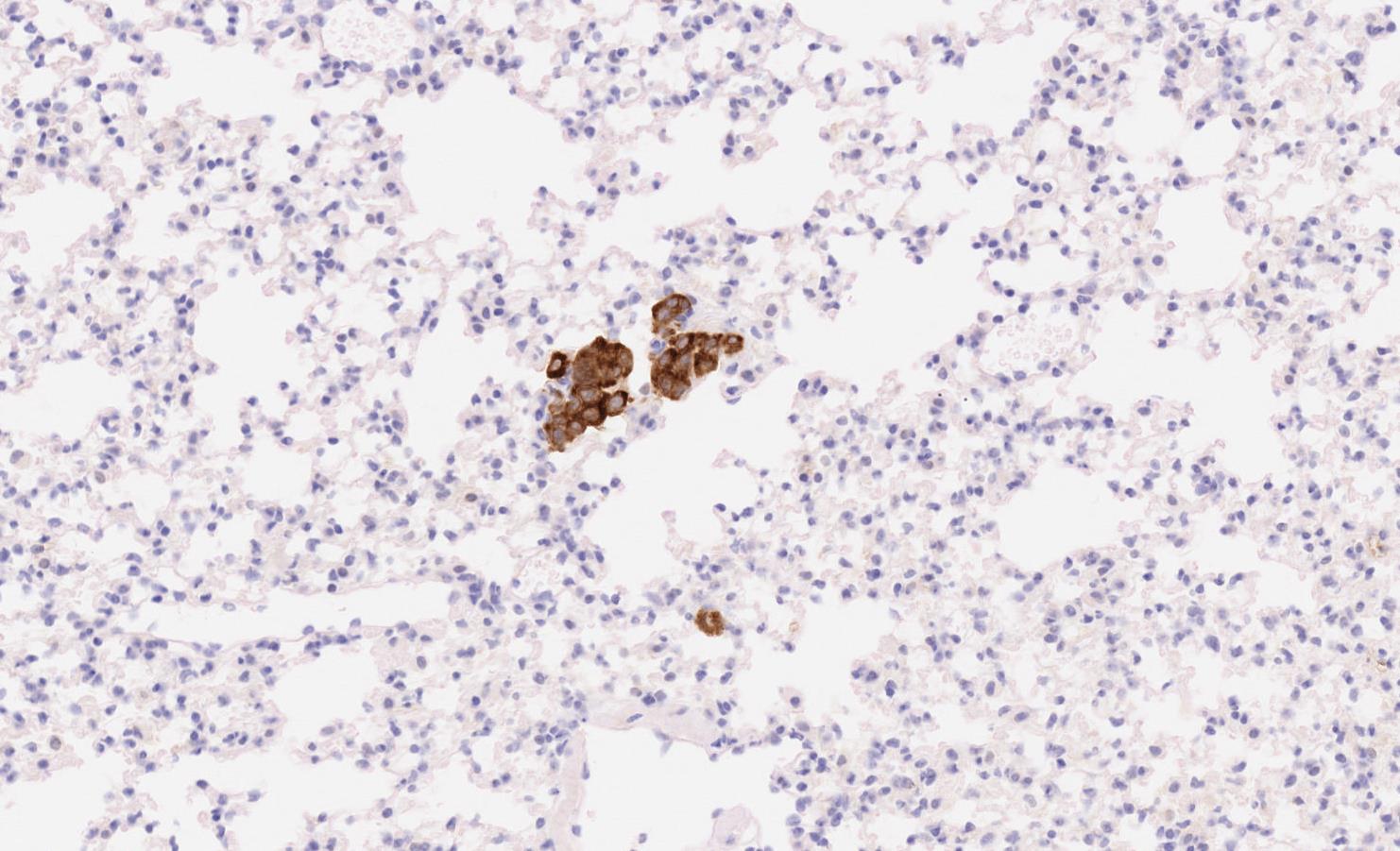

Supplement: Supplementary file 7 — Source data Fig. 6 [file 44321_2024_142_MOESM7_ESM.zip › Figure 6/J/sgSEPSECS 90 5c s3 Cas9 20x.jpg]

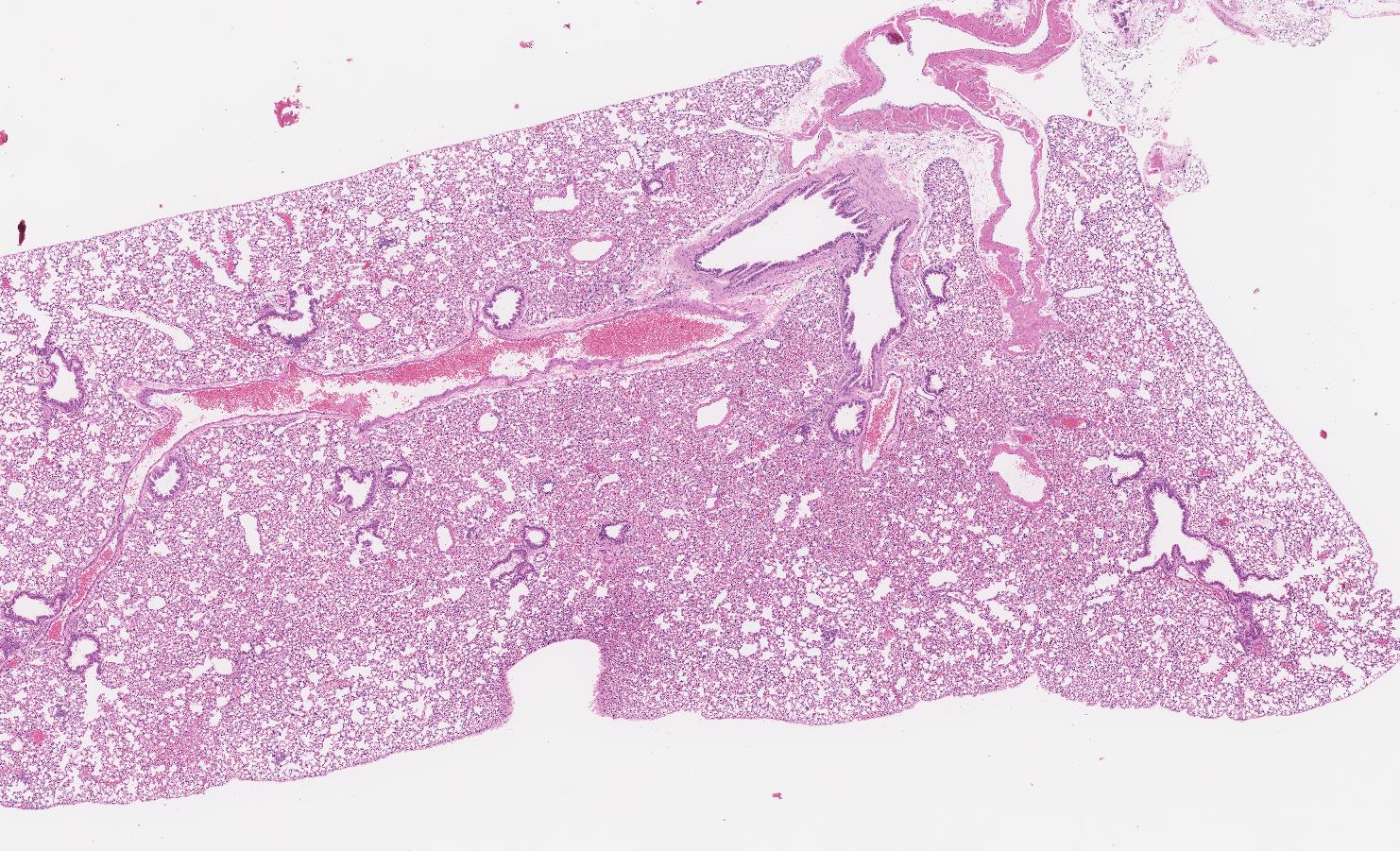

Supplement: Supplementary file 7 — Source data Fig. 6 [file 44321_2024_142_MOESM7_ESM.zip › Figure 6/J/sgSEPSECS 90 5c s1 HE 2x.jpg]

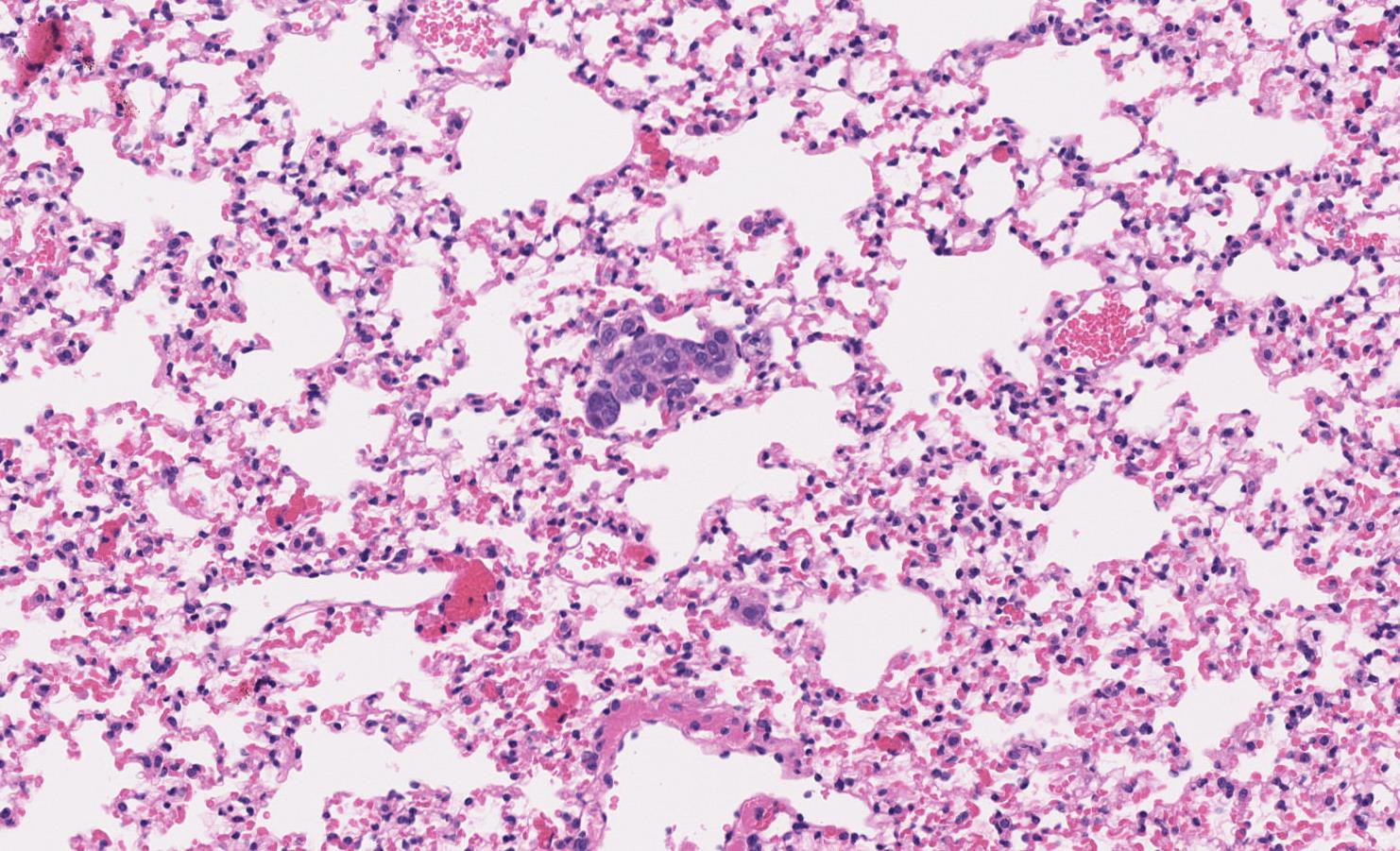

Supplement: Supplementary file 7 — Source data Fig. 6 [file 44321_2024_142_MOESM7_ESM.zip › Figure 6/J/sgSEPSECS 90 5c s1 HE 20x.jpg]

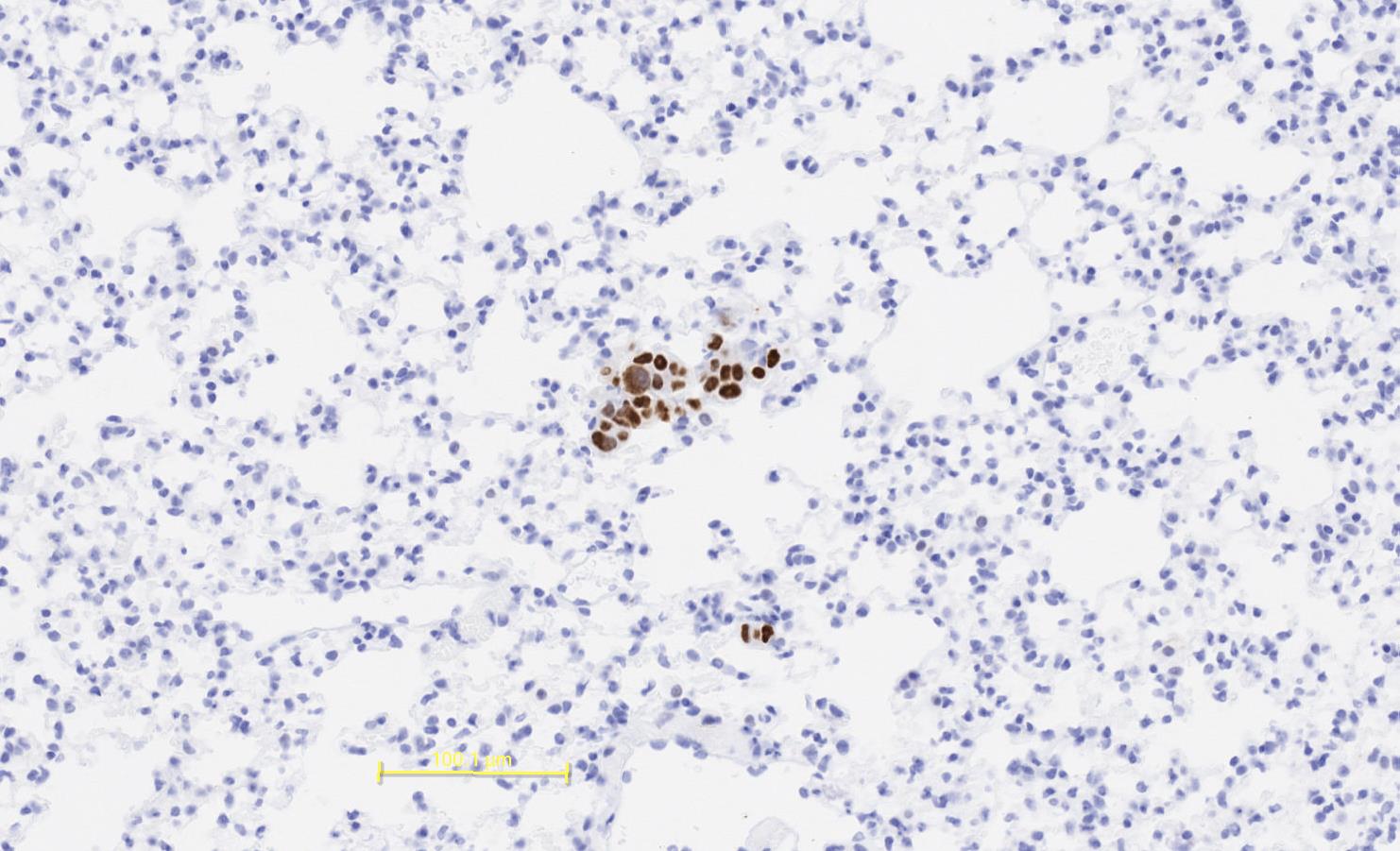

Supplement: Supplementary file 7 — Source data Fig. 6 [file 44321_2024_142_MOESM7_ESM.zip › Figure 6/J/sgSEPSECS 90 5c s2 Ku80 20x with scale.jpg]

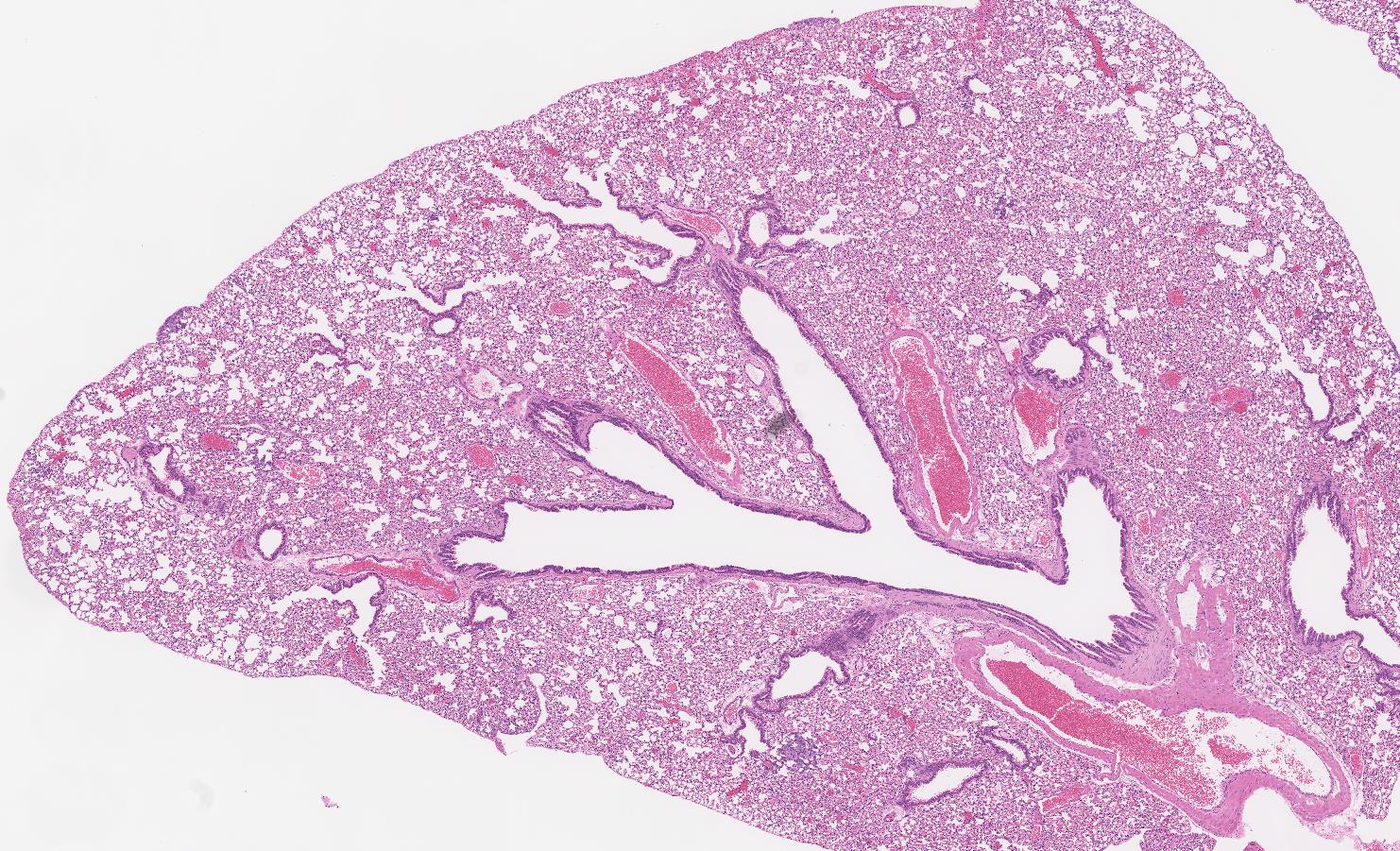

Supplement: Supplementary file 7 — Source data Fig. 6 [file 44321_2024_142_MOESM7_ESM.zip › Figure 6/J/NTC 90 2d s11 HE 2x.jpg]

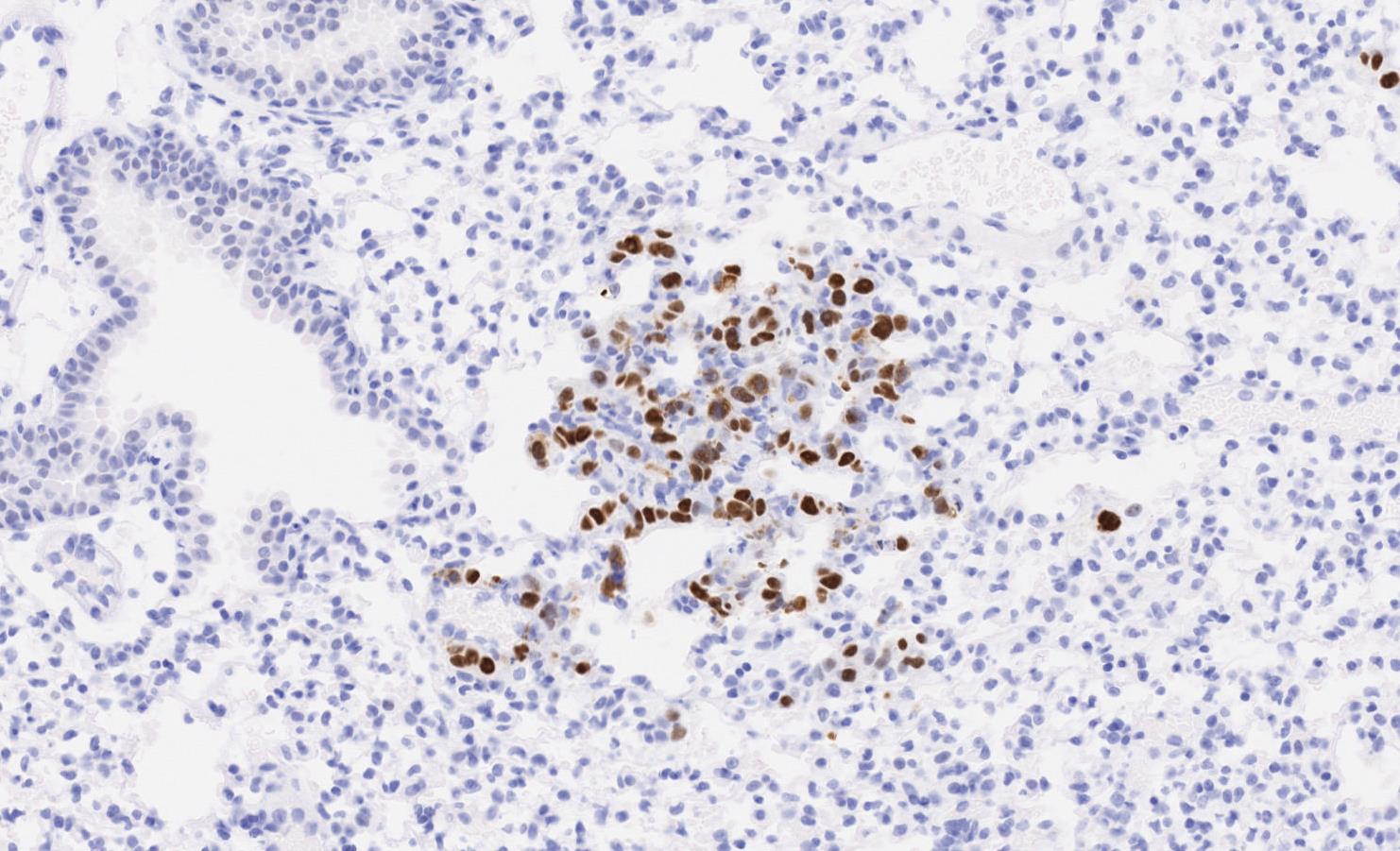

Supplement: Supplementary file 7 — Source data Fig. 6 [file 44321_2024_142_MOESM7_ESM.zip › Figure 6/J/NTC 90 2d s13 ku80 20x.jpg]

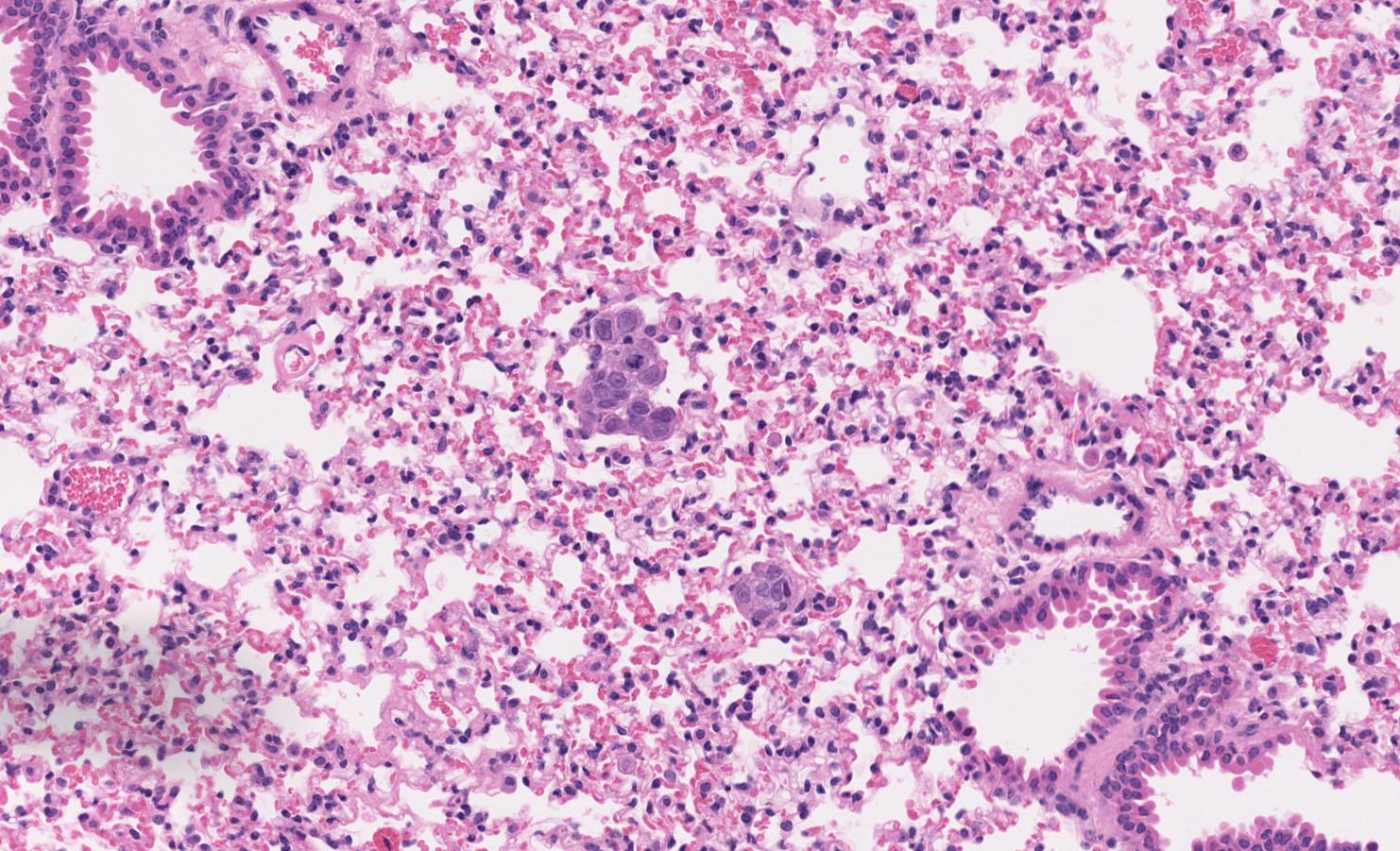

Supplement: Supplementary file 7 — Source data Fig. 6 [file 44321_2024_142_MOESM7_ESM.zip › Figure 6/J/sgSEPHS2 90 4b s11 HE 20x.jpg]

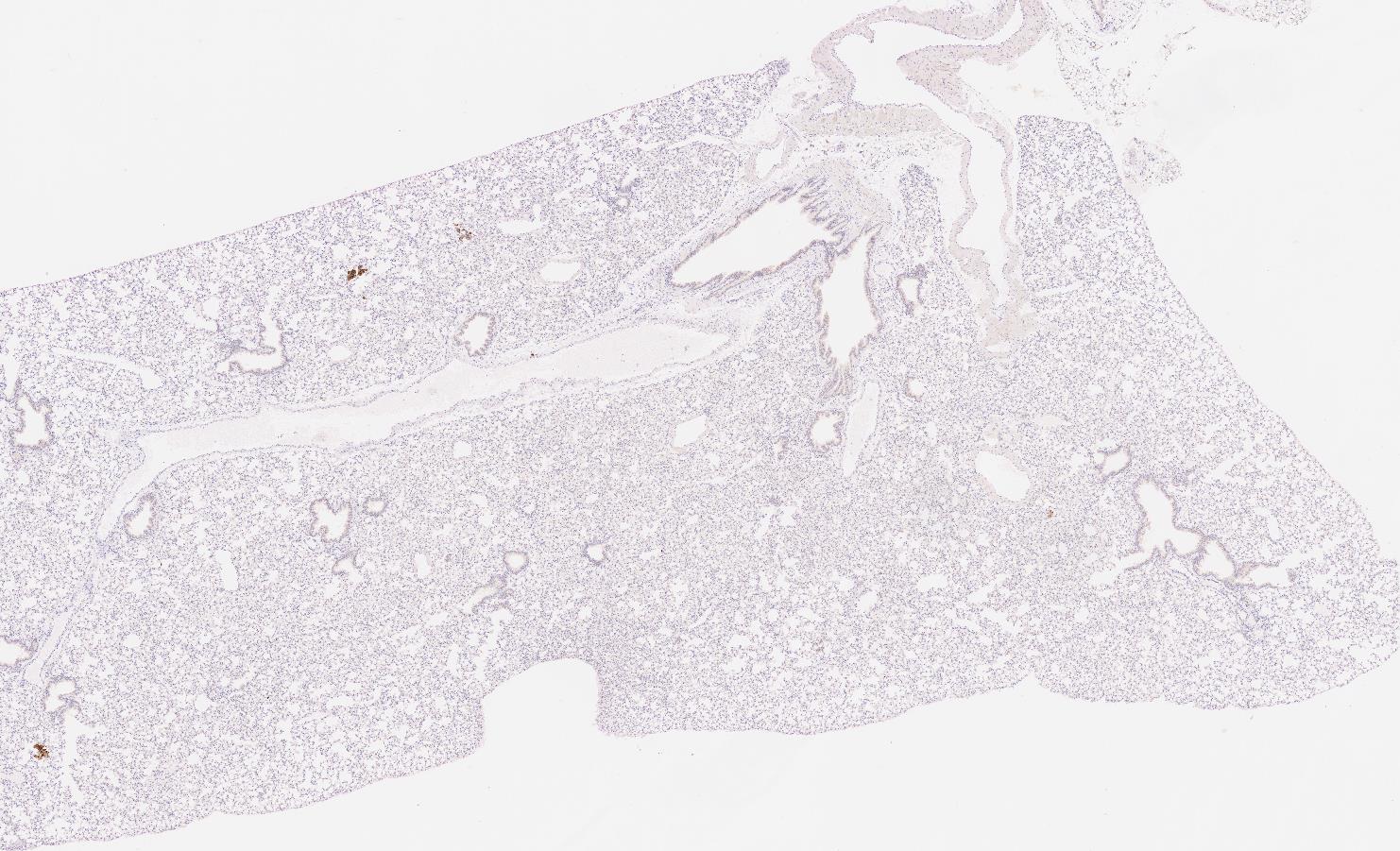

Supplement: Supplementary file 7 — Source data Fig. 6 [file 44321_2024_142_MOESM7_ESM.zip › Figure 6/J/sgSEPSECS 90 5c s3 Cas9 2x.jpg]

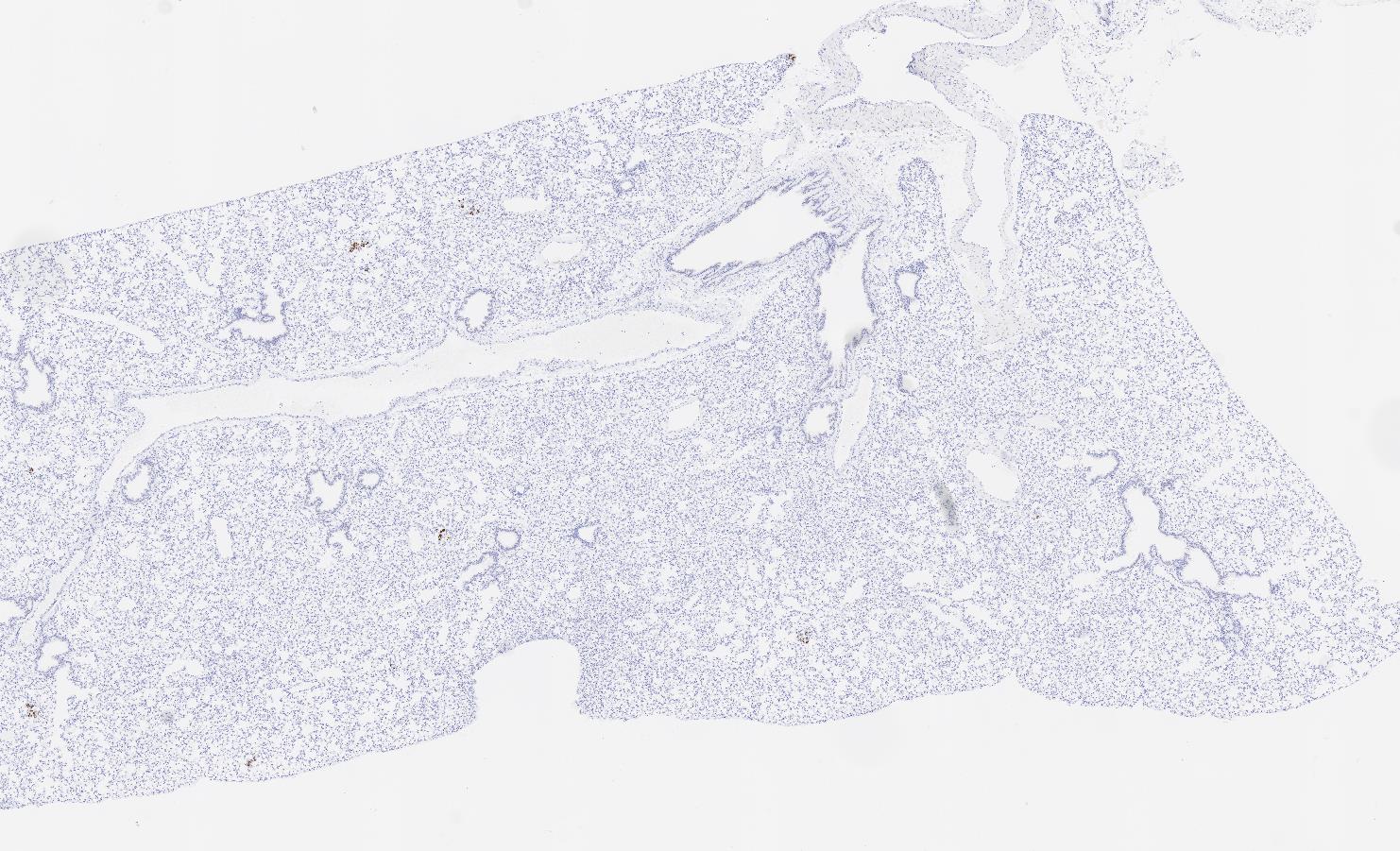

Supplement: Supplementary file 7 — Source data Fig. 6 [file 44321_2024_142_MOESM7_ESM.zip › Figure 6/J/sgSEPSECS 90 5c s2 Ku80 2x.jpg]

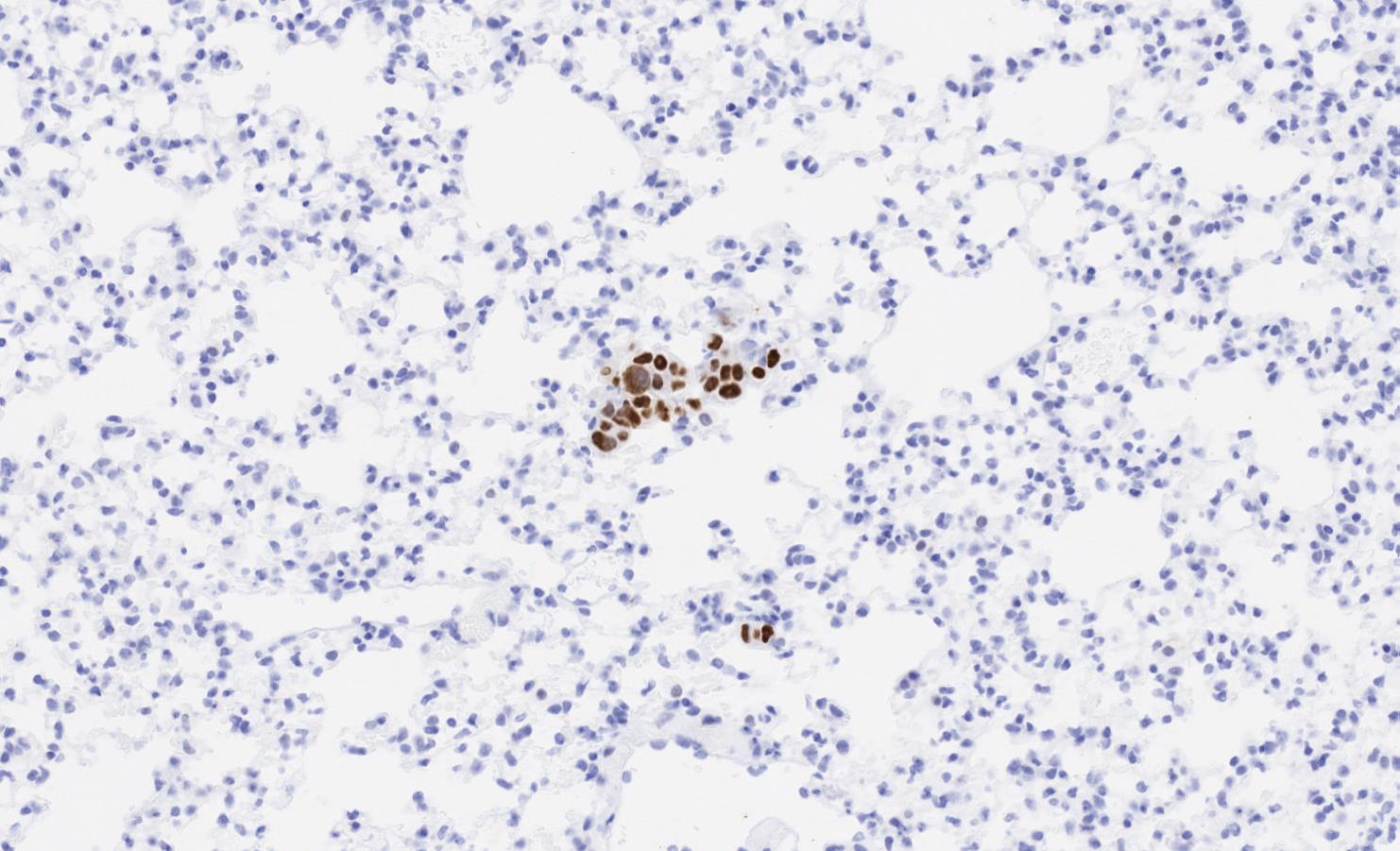

Supplement: Supplementary file 7 — Source data Fig. 6 [file 44321_2024_142_MOESM7_ESM.zip › Figure 6/J/sgSEPSECS 90 5c s2 Ku80 20x.jpg]

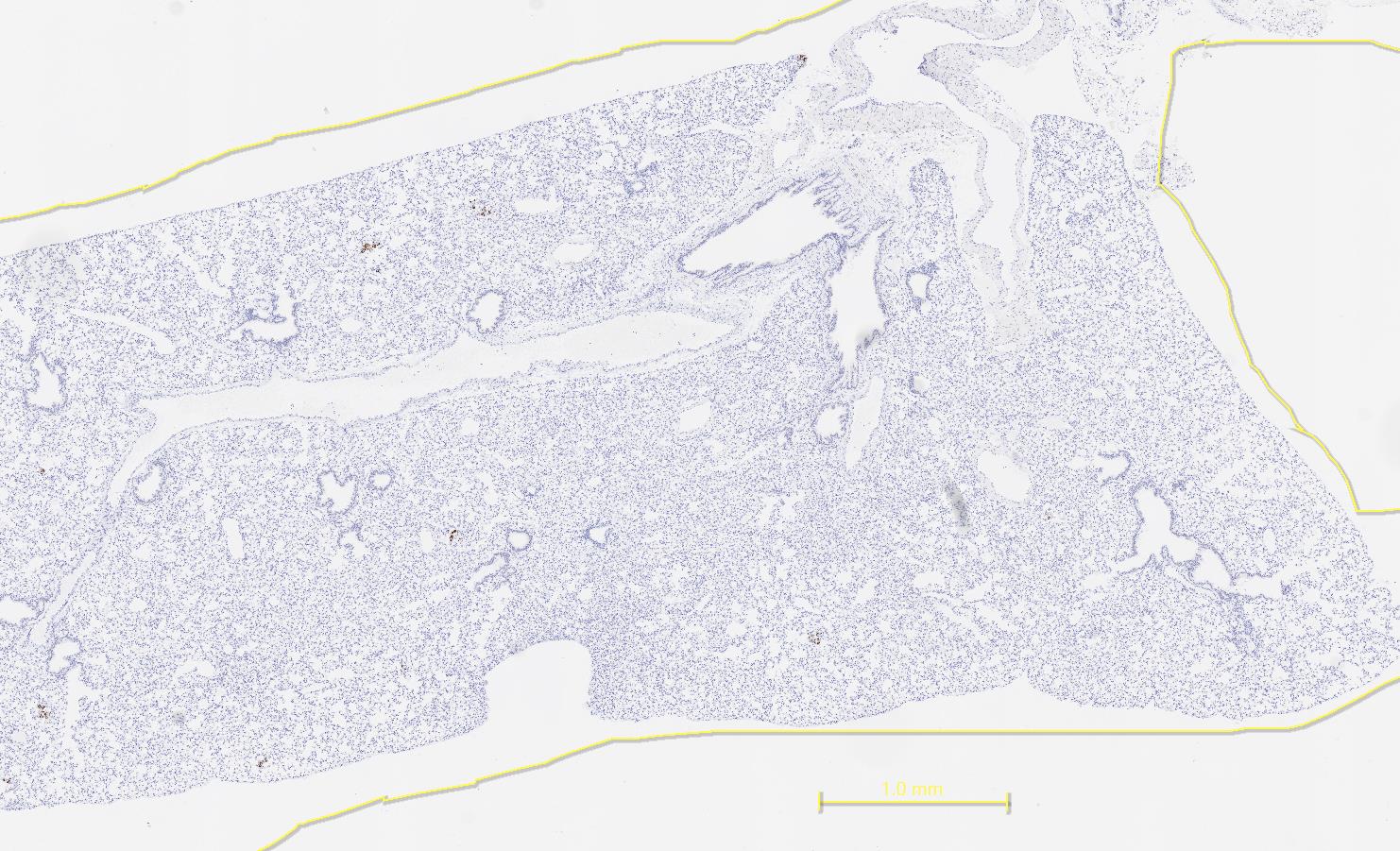

Supplement: Supplementary file 7 — Source data Fig. 6 [file 44321_2024_142_MOESM7_ESM.zip › Figure 6/J/sgSEPSECS 90 5c s2 Ku80 2x with scale.jpg]

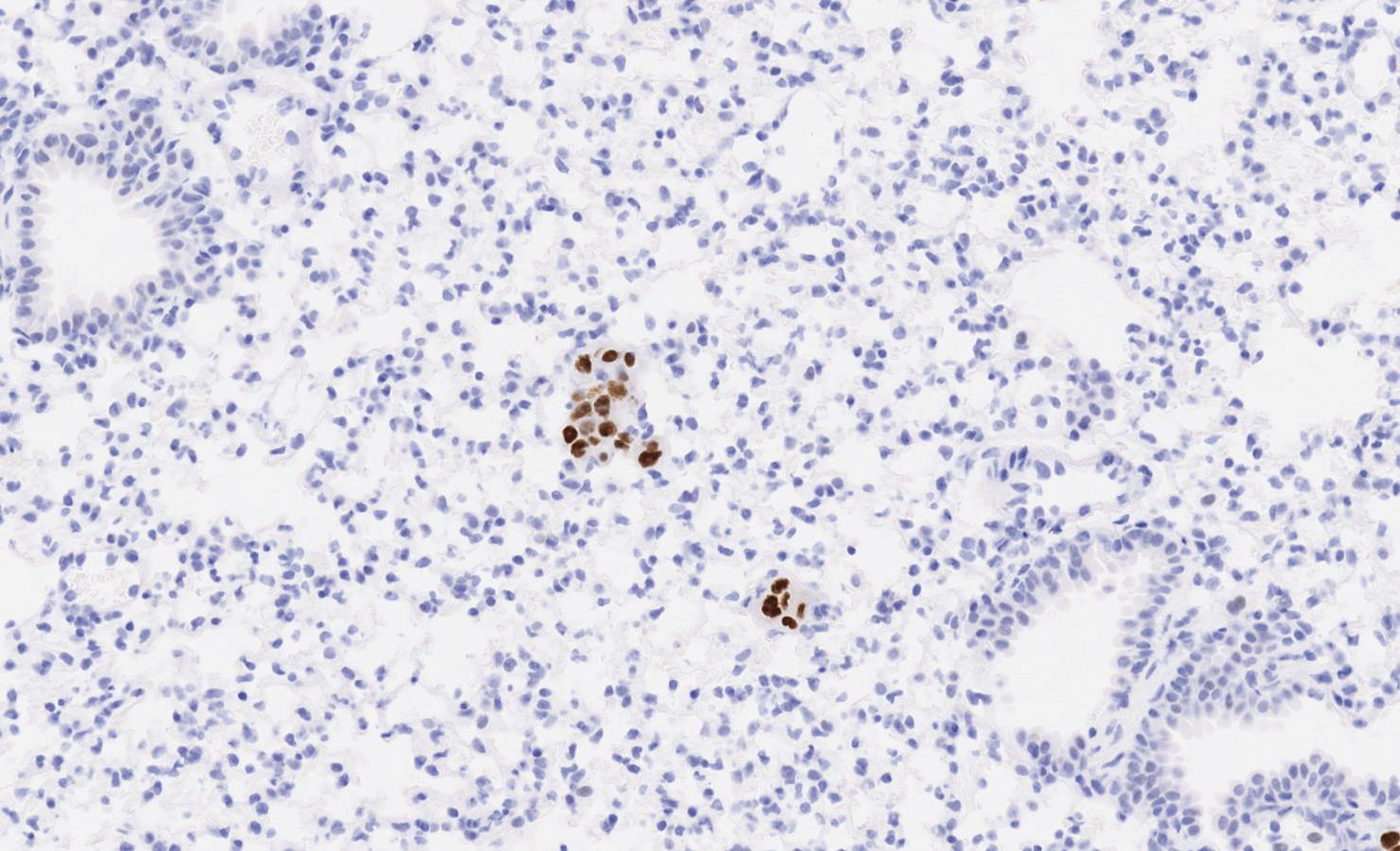

Supplement: Supplementary file 7 — Source data Fig. 6 [file 44321_2024_142_MOESM7_ESM.zip › Figure 6/J/sgSEPHS2 90 4b s12 ku80 20x.jpg]

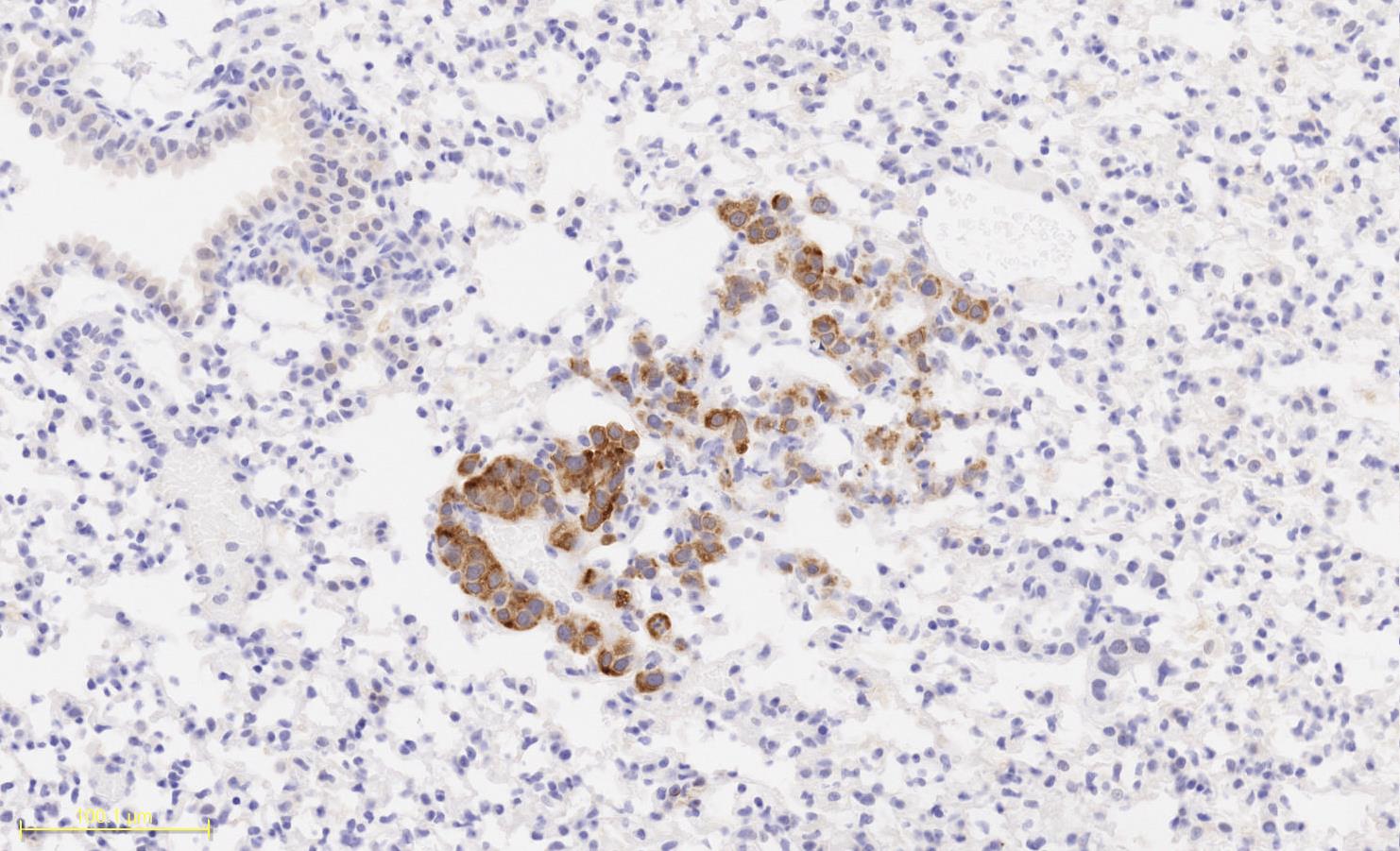

Supplement: Supplementary file 7 — Source data Fig. 6 [file 44321_2024_142_MOESM7_ESM.zip › Figure 6/J/NTC 90 2d s13 cas9 20x with scale.jpg]

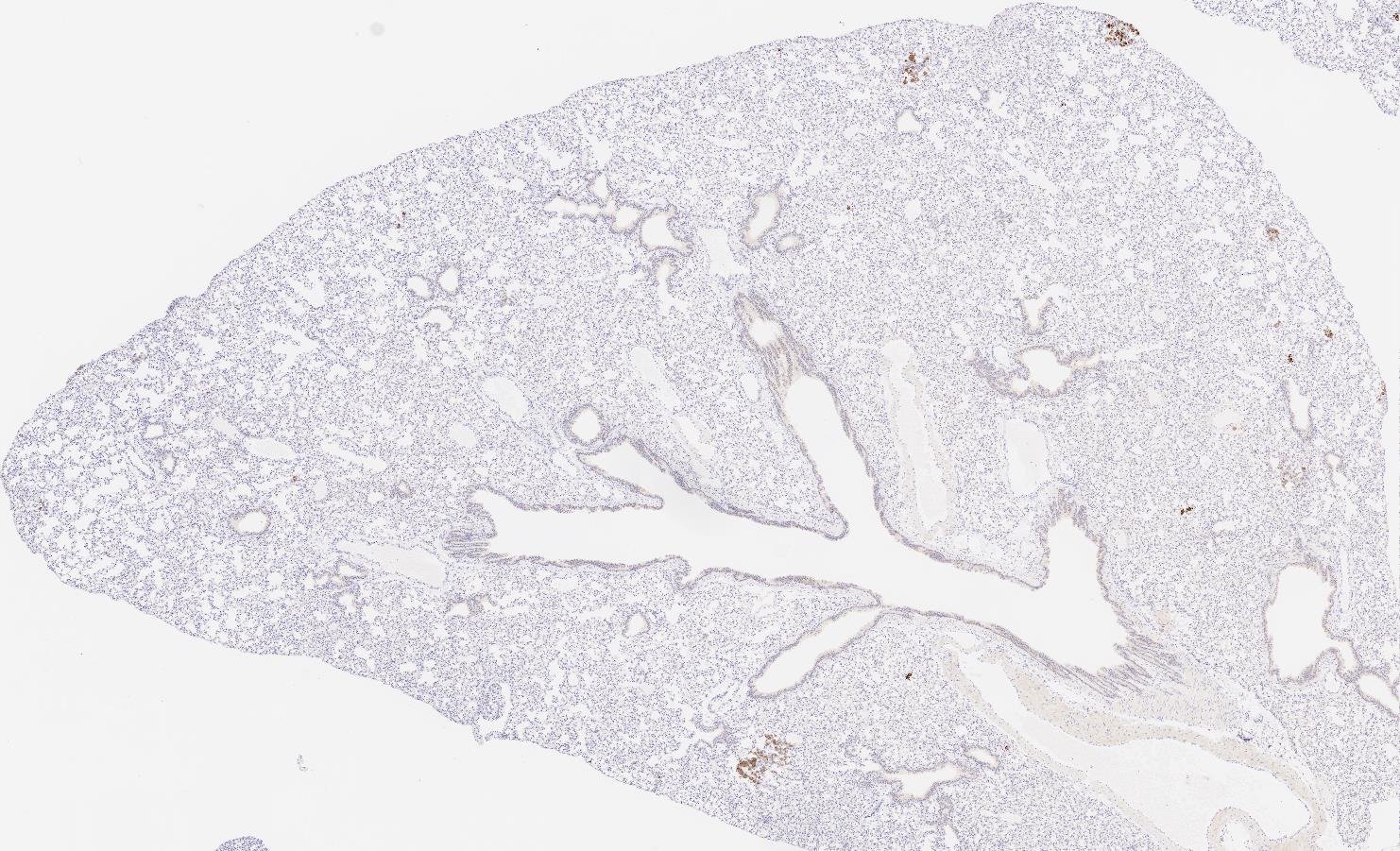

Supplement: Supplementary file 7 — Source data Fig. 6 [file 44321_2024_142_MOESM7_ESM.zip › Figure 6/J/NTC 90 2d s13 cas9 2x.jpg]

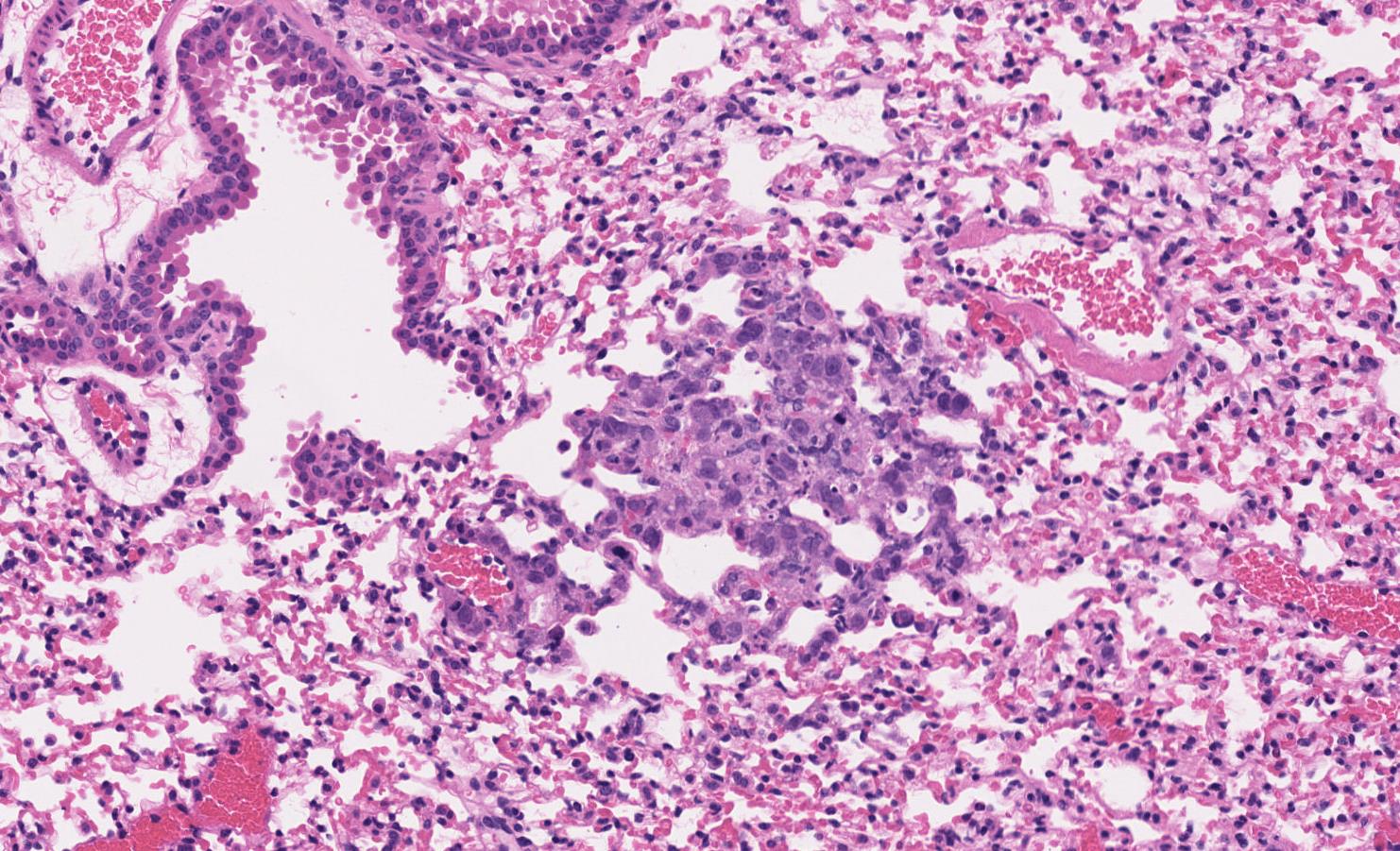

Supplement: Supplementary file 7 — Source data Fig. 6 [file 44321_2024_142_MOESM7_ESM.zip › Figure 6/J/NTC 90 2d s11 HE 20x.jpg]

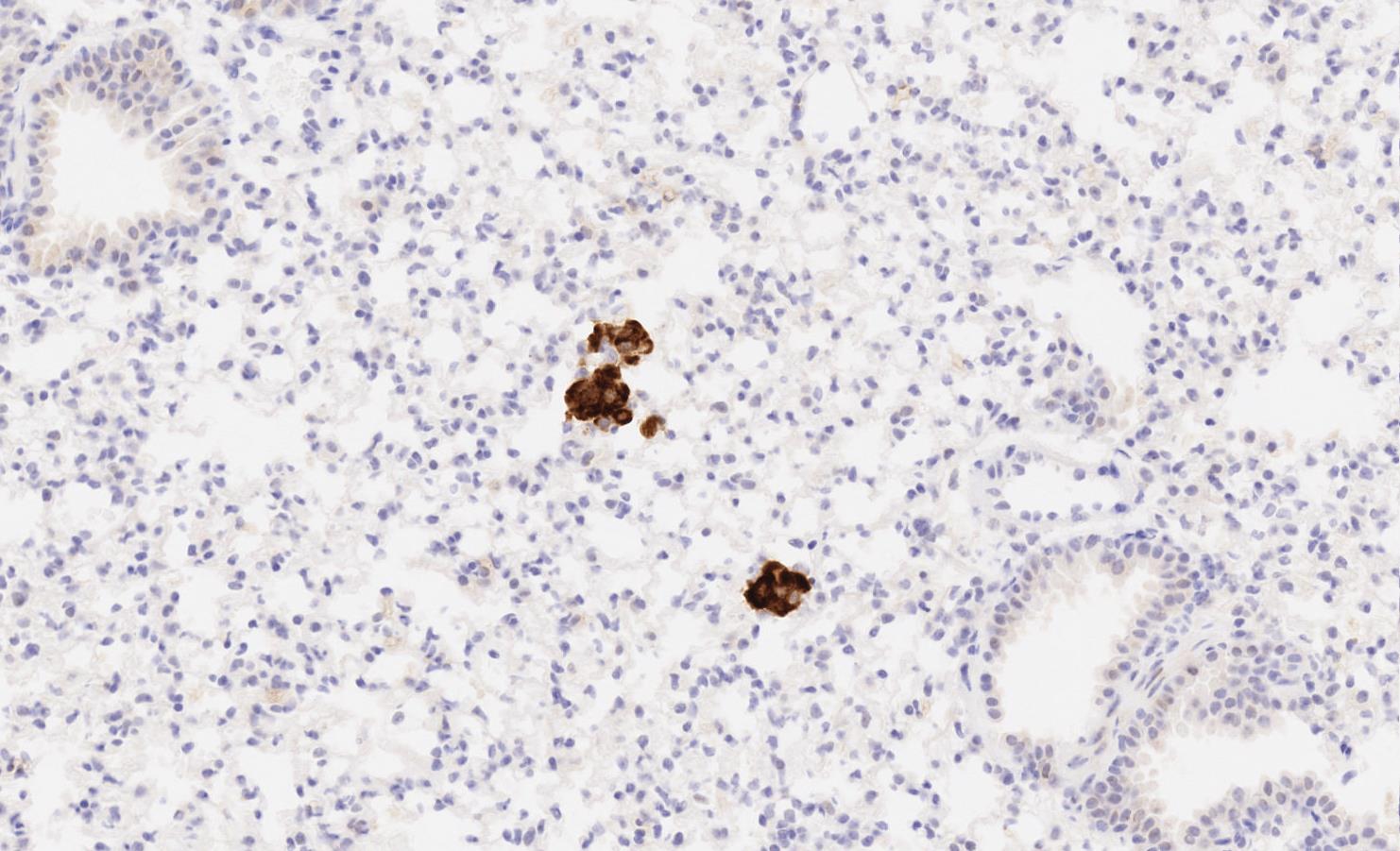

Supplement: Supplementary file 7 — Source data Fig. 6 [file 44321_2024_142_MOESM7_ESM.zip › Figure 6/J/sgSEPHS2 90 4b s13 cas9 20x.jpg]

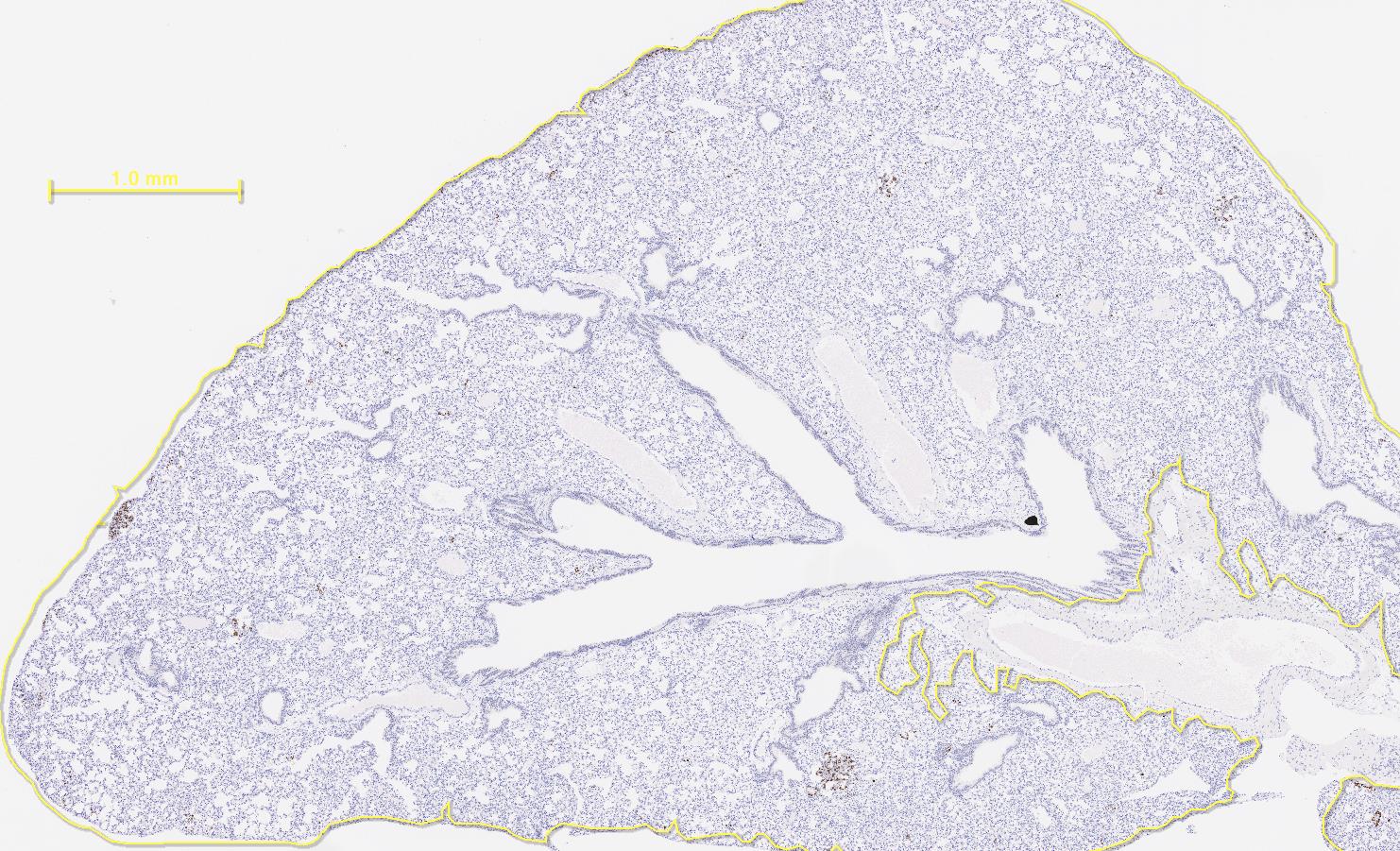

Supplement: Supplementary file 7 — Source data Fig. 6 [file 44321_2024_142_MOESM7_ESM.zip › Figure 6/J/NTC 90 2d s13 ku80 2x with scale.jpg]

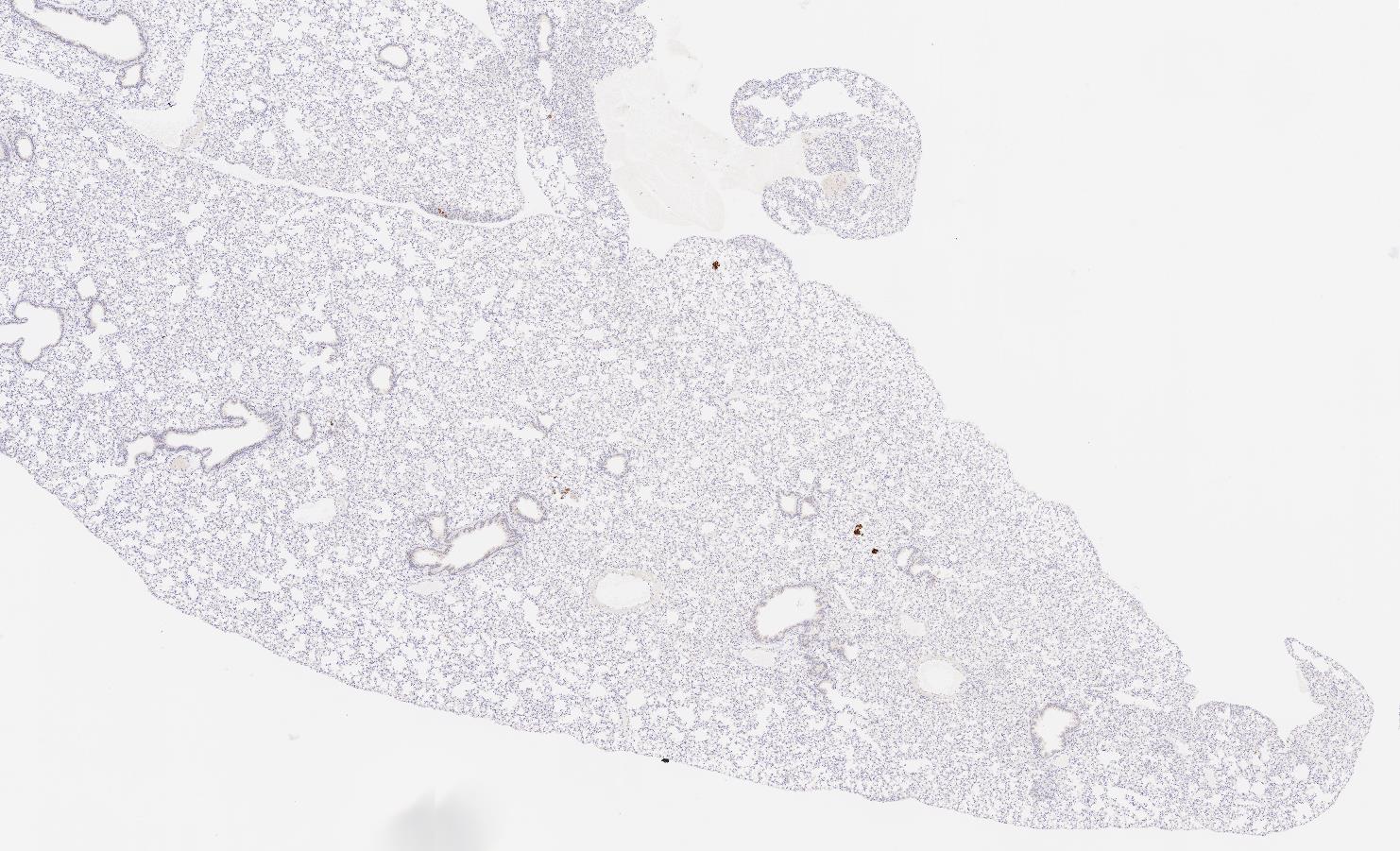

Supplement: Supplementary file 7 — Source data Fig. 6 [file 44321_2024_142_MOESM7_ESM.zip › Figure 6/J/sgSEPHS2 90 4b s13 cas9 2x.jpg]

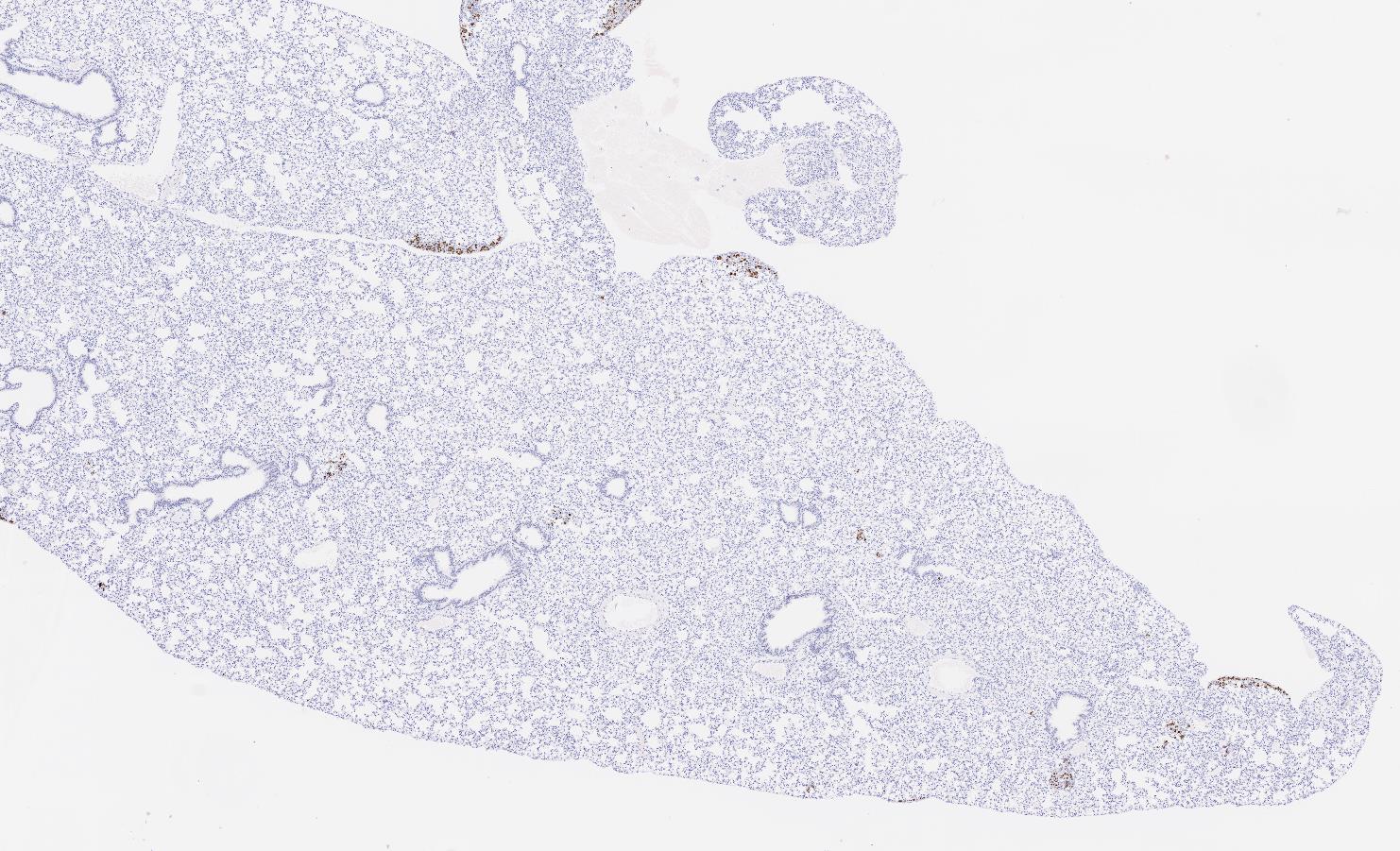

Supplement: Supplementary file 7 — Source data Fig. 6 [file 44321_2024_142_MOESM7_ESM.zip › Figure 6/J/sgSEPHS2 90 4b s12 ku80 2x.jpg]
