# Supplementary material for: Breast cancer secretes anti-ferroptotic MUFAs and depends on selenoprotein synthesis for metastasis
Source: EMBO Mol Med. 2024 Oct 21;16(11):7. doi: 10.1038/s44321-024-00142-x (PMC11555046; doi:10.1038/s44321-024-00142-x)
Supplement: Supplementary file 6 — Source data Fig. 5 [file 44321_2024_142_MOESM6_ESM.zip › Figure 5/G/full scan and labels.pptx]

## Slide 1
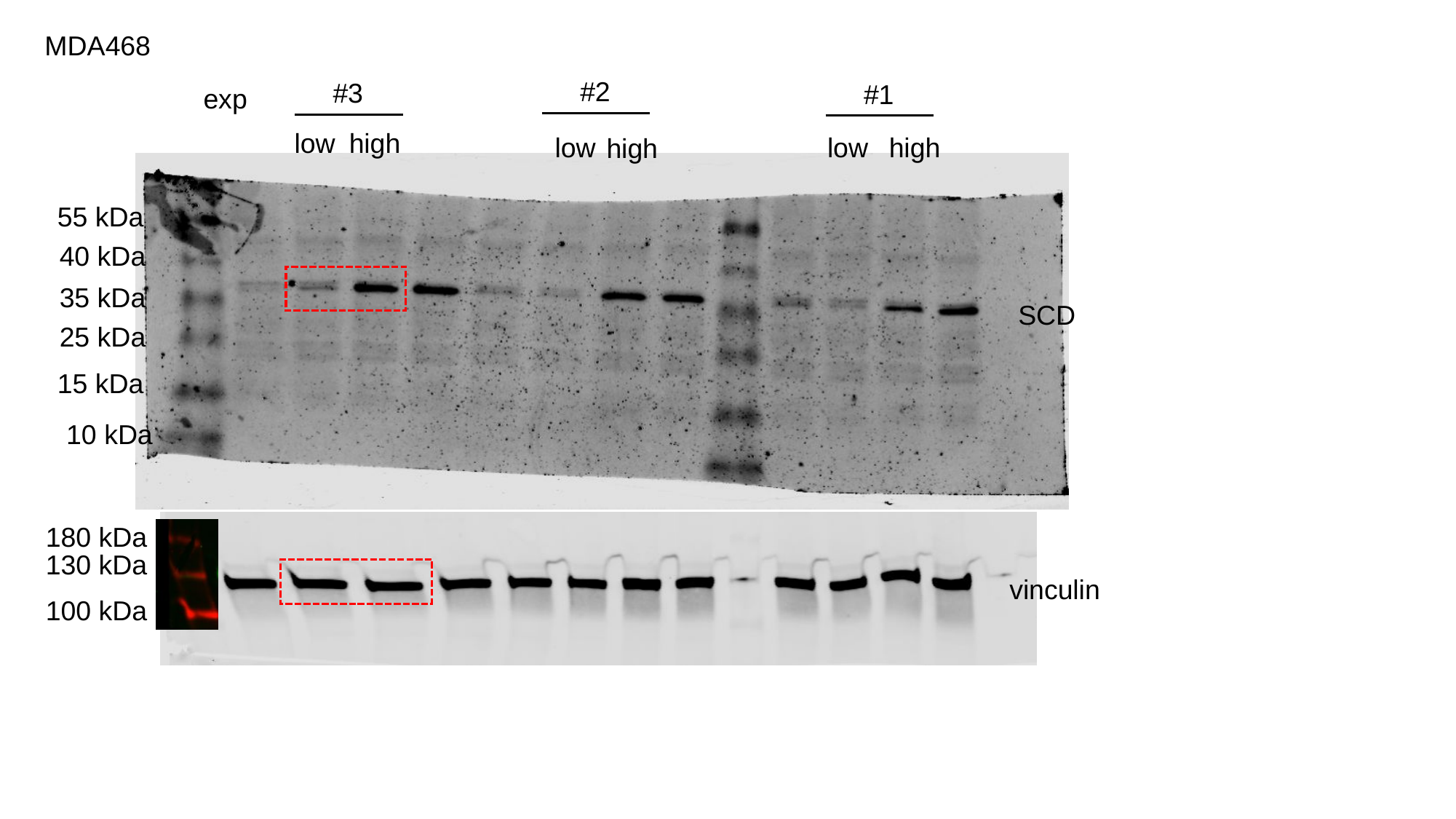

MDA468
#2
#3
#1
exp
low
high
low
low
high
high
55 kDa
40 kDa
35 kDa
SCD
25 kDa
15 kDa
10 kDa
180 kDa
130 kDa
vinculin
100 kDa

## Slide 2
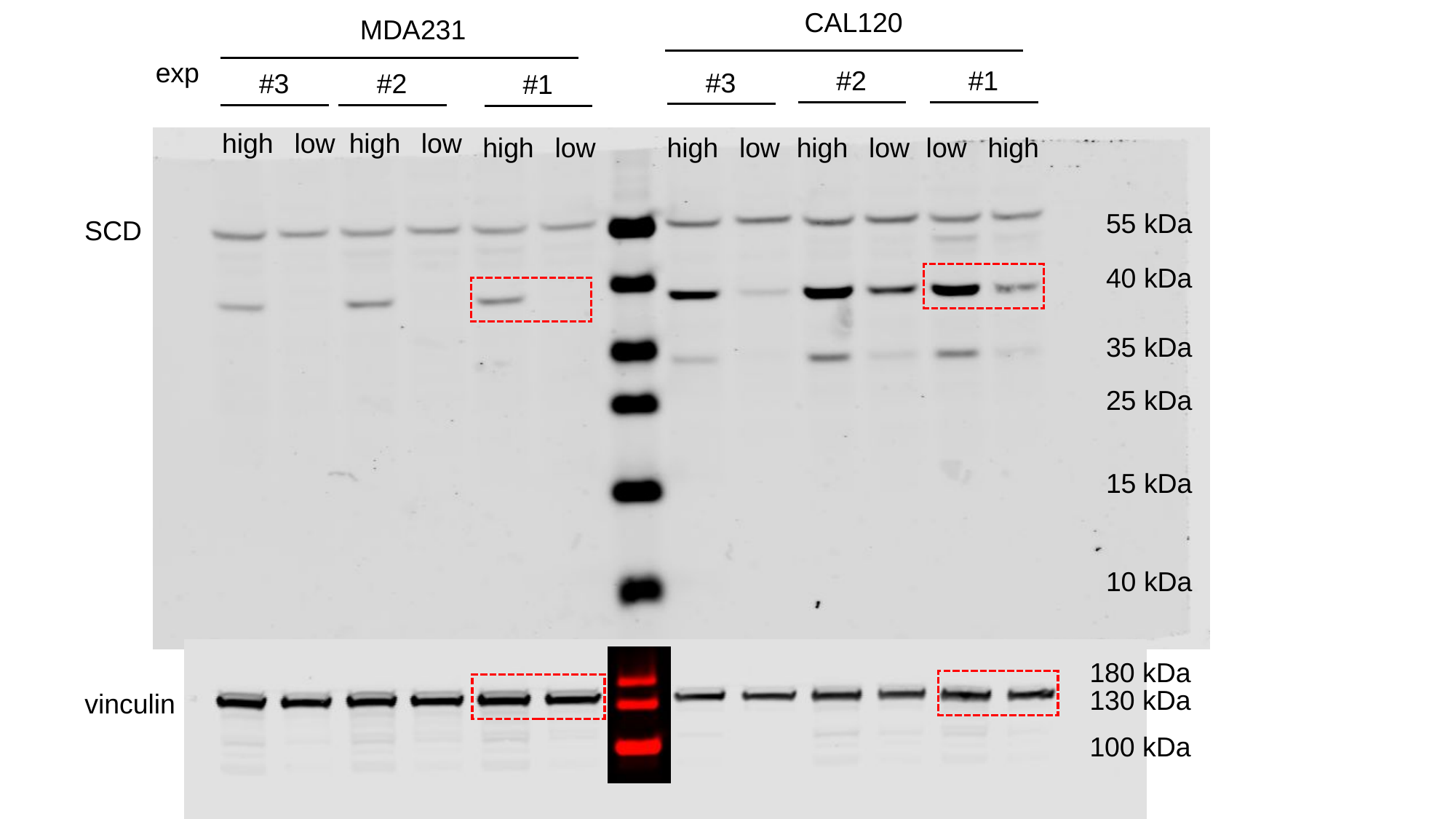

CAL120
MDA231
exp
#1
#2
#3
#3
#2
#1
high
low
high
low
high
low
high
low
high
low
low
high
55 kDa
SCD
40 kDa
35 kDa
25 kDa
15 kDa
10 kDa
180 kDa
130 kDa
vinculin
100 kDa

## Slide 3
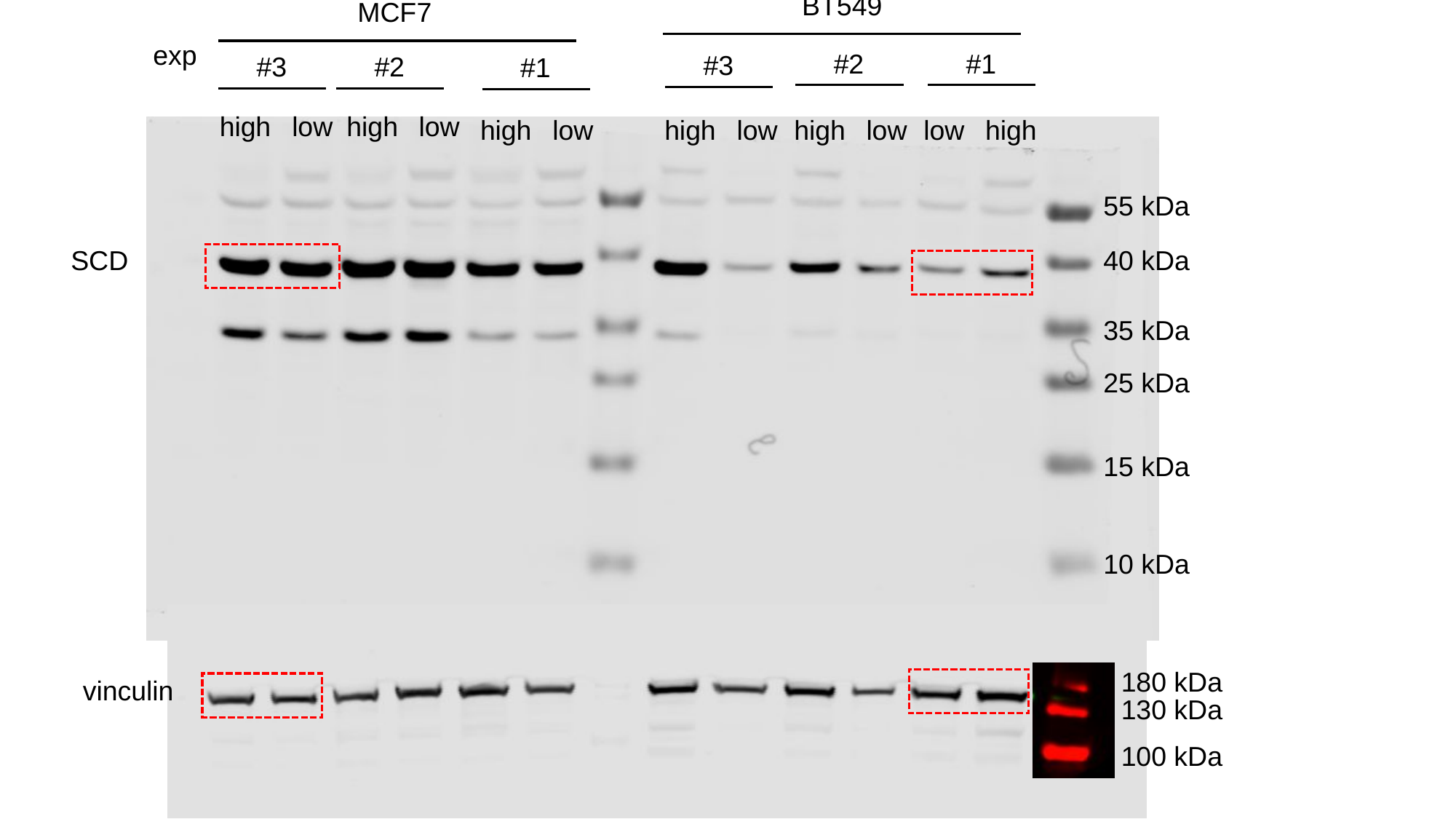

BT549
MCF7
exp
#1
#2
#3
#3
#2
#1
high
low
high
low
high
low
high
low
high
low
low
high
55 kDa
SCD
40 kDa
35 kDa
25 kDa
15 kDa
10 kDa
180 kDa
vinculin
130 kDa
100 kDa

## Slide 4
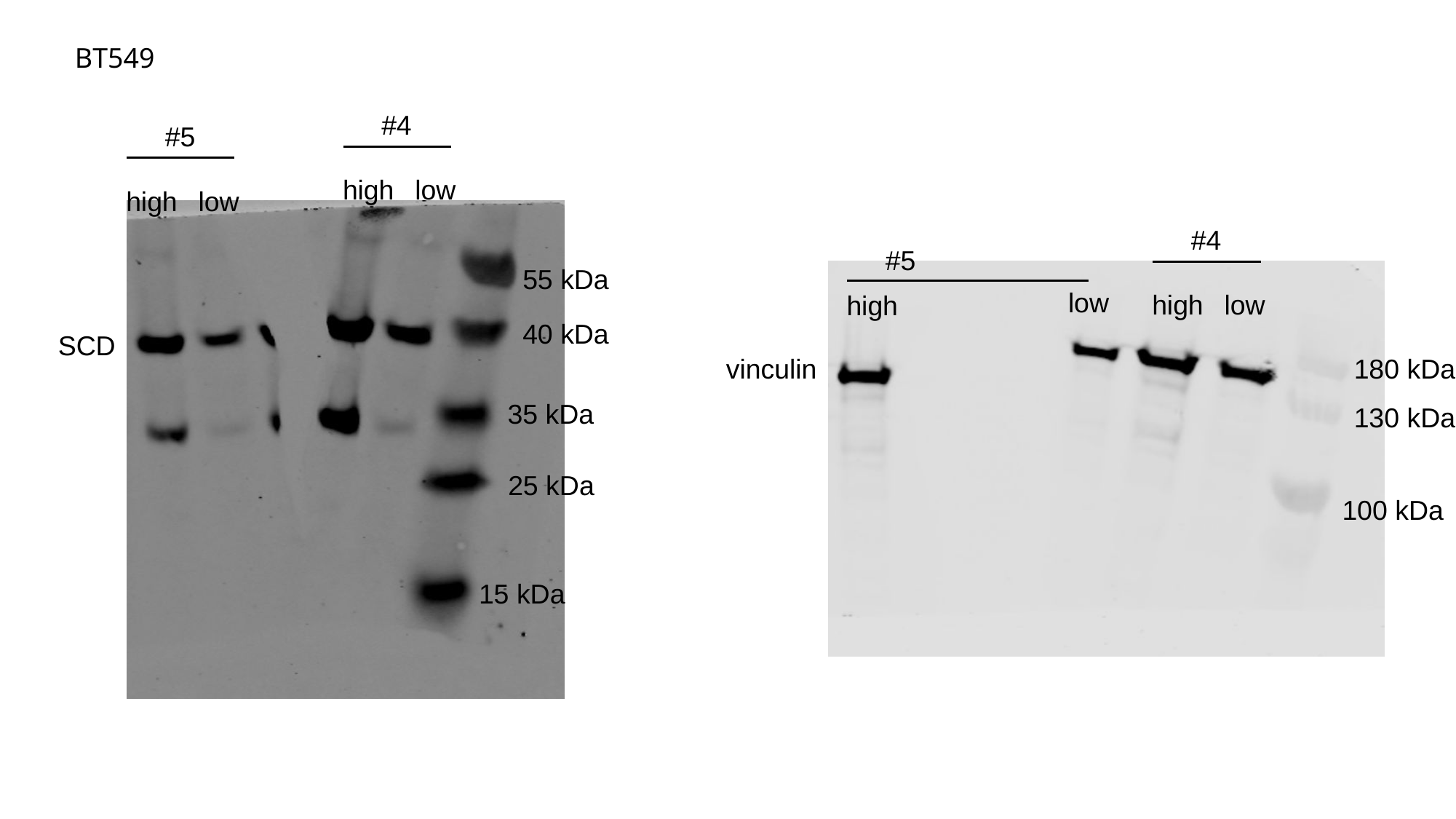

BT549
#4
#5
high
low
high
low
#4
#5
55 kDa
low
high
low
high
40 kDa
SCD
vinculin
180 kDa
35 kDa
130 kDa
25 kDa
100 kDa
15 kDa
