# Supplementary material for: Breast cancer secretes anti-ferroptotic MUFAs and depends on selenoprotein synthesis for metastasis
Source: EMBO Mol Med. 2024 Oct 21;16(11):7. doi: 10.1038/s44321-024-00142-x (PMC11555046; doi:10.1038/s44321-024-00142-x)
Supplement: Supplementary file 7 — Source data Fig. 6 [file 44321_2024_142_MOESM7_ESM.zip › Figure 6/E/full scan image with label.pptx]

## Slide 1
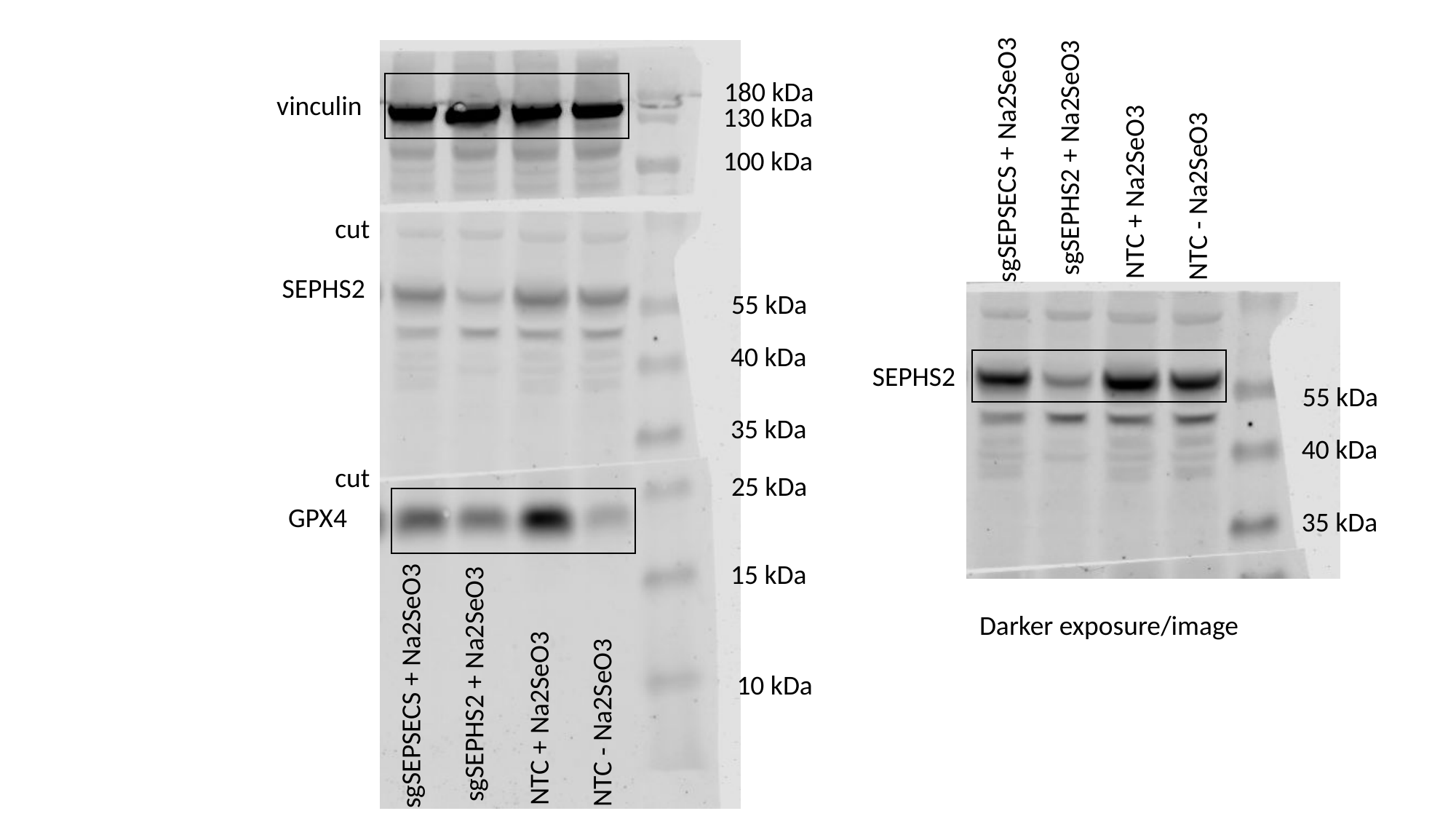

180 kDa
vinculin
130 kDa
sgSEPHS2 + Na2SeO3
sgSEPSECS + Na2SeO3
100 kDa
NTC + Na2SeO3
NTC - Na2SeO3
cut
SEPHS2
55 kDa
40 kDa
SEPHS2
55 kDa
35 kDa
40 kDa
cut
25 kDa
GPX4
35 kDa
15 kDa
Darker exposure/image
10 kDa
sgSEPHS2 + Na2SeO3
sgSEPSECS + Na2SeO3
NTC + Na2SeO3
NTC - Na2SeO3
