# Supplementary material for: Breast cancer secretes anti-ferroptotic MUFAs and depends on selenoprotein synthesis for metastasis
Source: EMBO Mol Med. 2024 Oct 21;16(11):7. doi: 10.1038/s44321-024-00142-x (PMC11555046; doi:10.1038/s44321-024-00142-x)
Supplement: Supplementary file 7 — Source data Fig. 6 [file 44321_2024_142_MOESM7_ESM.zip › Figure 6/B/full scan image with label.pptx]

## Slide 1
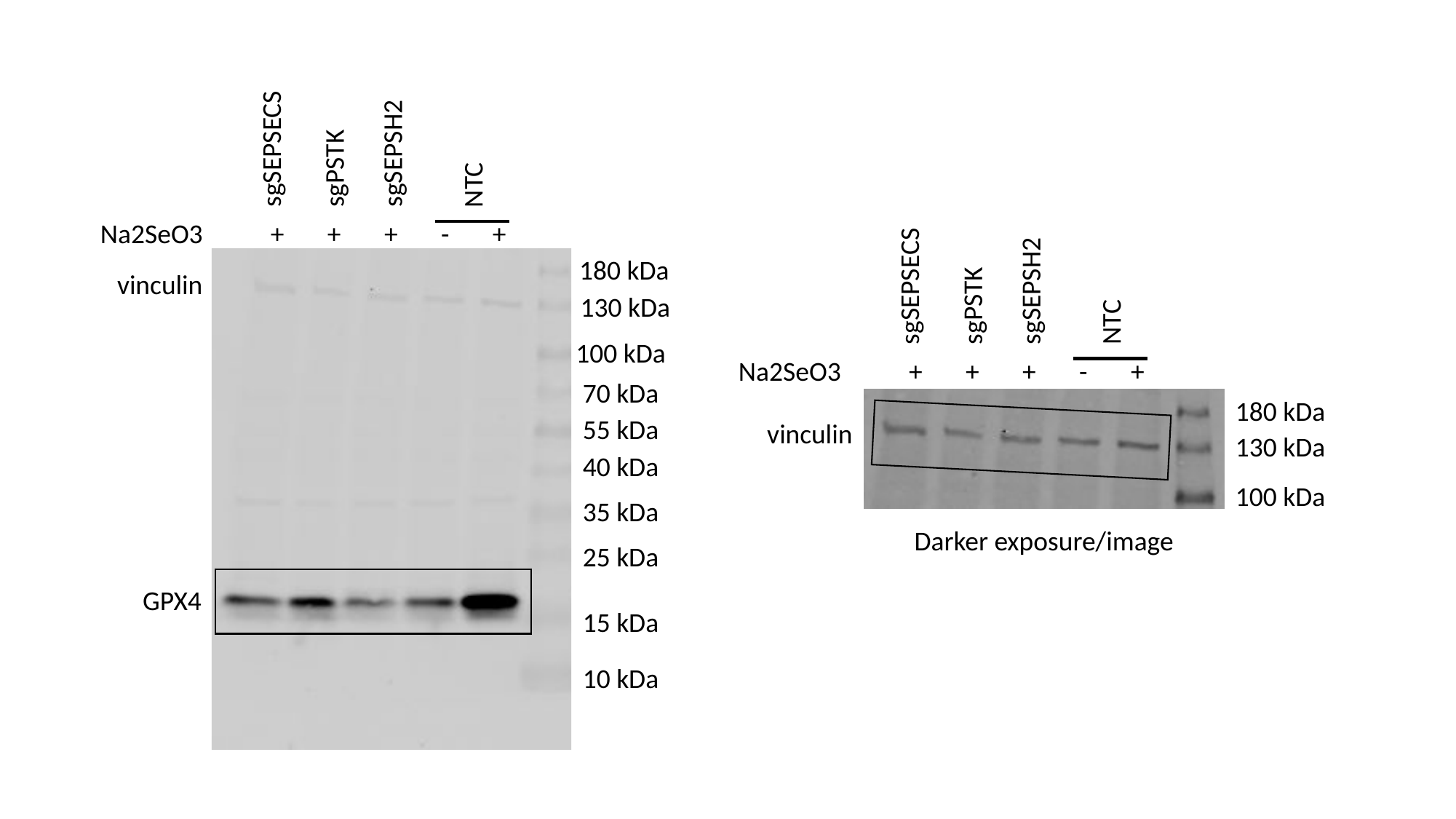

sgSEPSECS
sgSEPSH2
sgPSTK
NTC
Na2SeO3 + + + - +
180 kDa
vinculin
sgSEPSECS
sgSEPSH2
sgPSTK
130 kDa
NTC
100 kDa
Na2SeO3 + + + - +
70 kDa
180 kDa
55 kDa
vinculin
130 kDa
40 kDa
100 kDa
35 kDa
Darker exposure/image
25 kDa
GPX4
15 kDa
10 kDa
