# Supplementary material for: Breast cancer secretes anti-ferroptotic MUFAs and depends on selenoprotein synthesis for metastasis
Source: EMBO Mol Med. 2024 Oct 21;16(11):7. doi: 10.1038/s44321-024-00142-x (PMC11555046; doi:10.1038/s44321-024-00142-x)
Supplement: Supplementary file 9 — Expanded View Figures [file 44321_2024_142_MOESM9_ESM.pdf]

Expanded View Figures

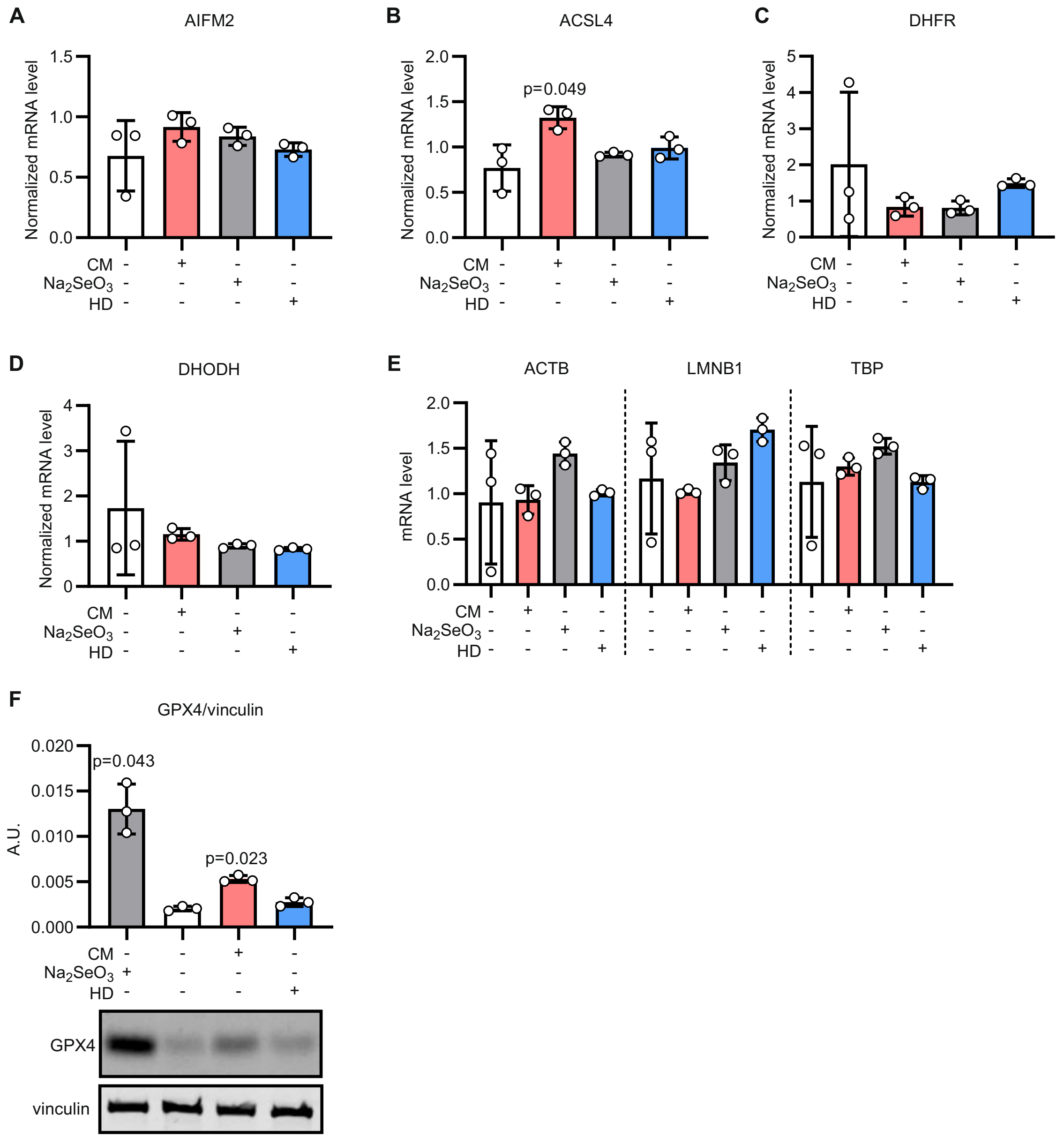

Figure EV1. Breast cancer cells produce an anti-ferroptosis molecule at high density.

(**A–D**) qPCR quantification of *AIFM2* (**A**), *ACSL4* (**B**), *DHFR* (**C**), and *DHODH* (**D**) mRNA expression in MDA-MB-468 cells seeded at low density in mock medium and supplemented with 50 nM selenite or conditioned medium (CM) for 2 days as indicated. The gene expression was also assessed in cells seeded at high density (HD) grown for 2 days in mock medium. The mRNA level is normalised to the mean mRNA abundance of the three housekeeping genes (*ACTB, LMNB1*, and *TBP*) shown in (**E**). *P* values refer to a one-way ANOVA for paired samples with Dunnett's multiple comparisons test. $n_{exp} = 3$. Bars represent mean ± s.d. (**E**) qPCR quantification of *ACTB, LMNB1*, and *TBP* mRNA expression in MDA-MB-468 cells seeded and treated as described in A–D. $n_{exp} = 3$. Bars represent mean ± s.d. (**F**) Immunoblot analysis of GPX4 and vinculin (loading control) in MDA-MB-468 cells MDA-MB-468 cells seeded and treated as described in (**A–D**). A.U.: arbitrary unit. Representative images of GPX4 and vinculin (loading control) from one of the three experiments quantified in the upper graph. *P* values refer to a one-way ANOVA for paired samples with Dunnett's multiple comparisons test. Bars represent mean ± s.d.

© The Author(s)

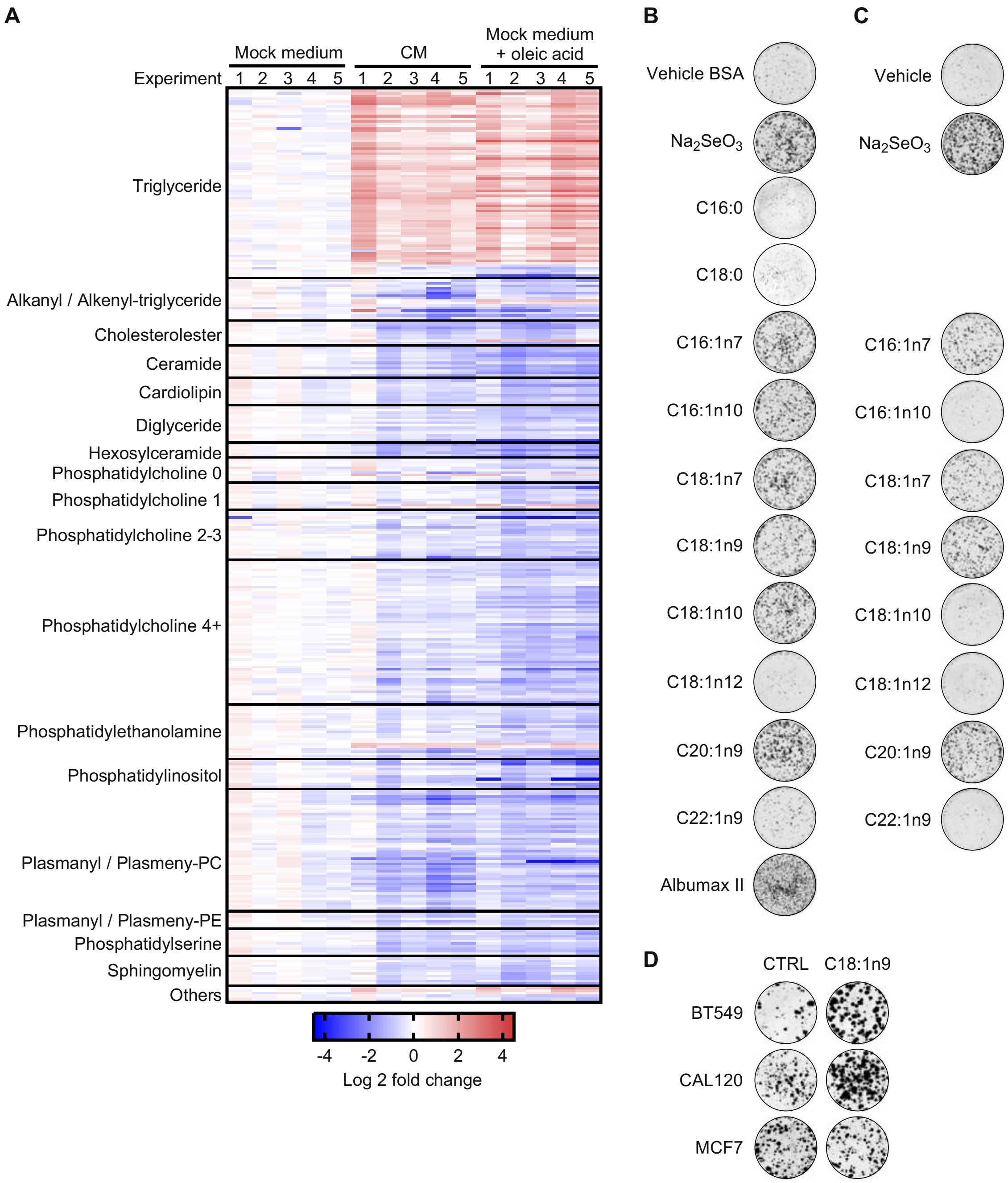

Figure EV2. Monounsaturated fatty acids are enriched in the conditioned medium and prevent ferroptosis.

(**A**) Heatmap of lipids regulated in MDA-MB-468 cells cultured at low density with mock medium, conditioned medium (CM) or mock medium with 10 μM oleic acid. Ferrostatin-1 was supplemented at 2 μM in all conditions. The lipids identified as significantly regulated with a two-tailed, homoscedastic Student's *t* tests for unpaired samples in the comparison between conditioned medium and mock medium are reported and selected classes of lipids are indicated. For the phosphatidylcholine class the number of double bonds is also reported (0, 1, 2-3, 4+). The Log2 fold change refers to the comparison with mock medium supplemented cells. $n_{exp} = 5$. (**B**) Representative images for the colony-forming assays displayed in Fig. 3D. (**C**) Representative images for the colony-forming assays displayed in Fig. 3E. (**D**) Representative images for colony-forming assays displayed in Fig. 3F.

 © The Author(s)

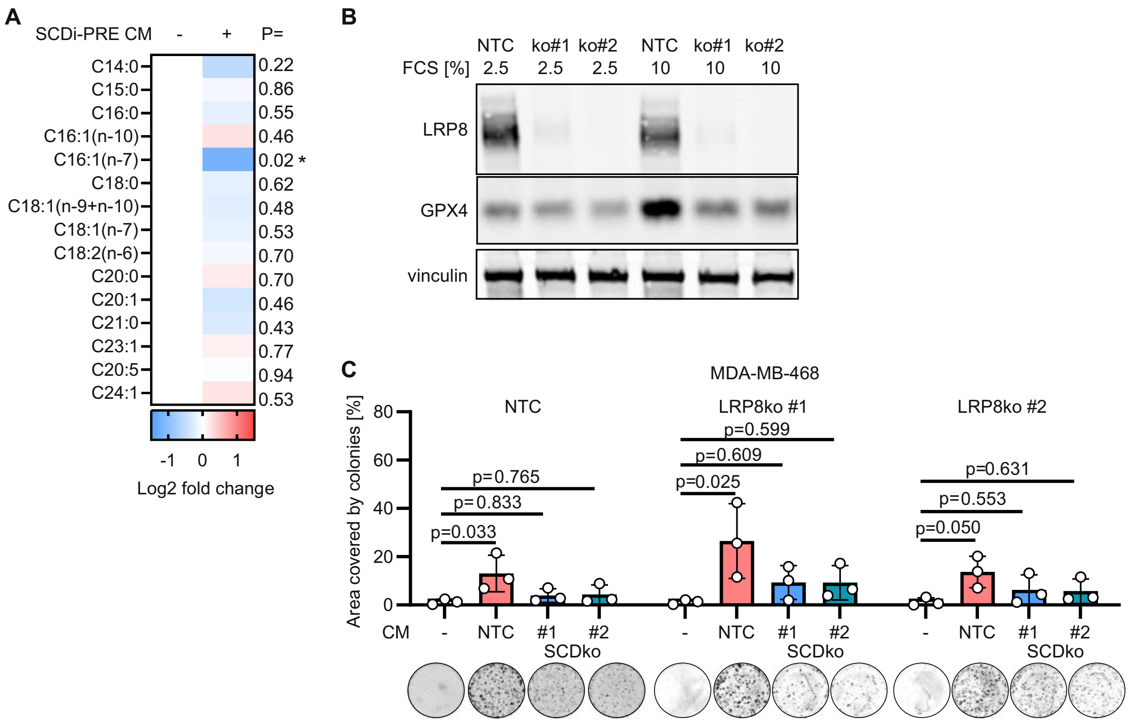

Figure EV3. SCD is required for the anti-ferroptotic capacities of the conditioned medium.

(**A**) Quantification of total (free and lipid-bound) fatty acid species in medium conditioned by MDA-MB-468 cells without or with SCD inhibitor pre-treatment (SCDi PRE). Peak area values normalised on the signal from internal standard (C17:0) were used to calculate the Log2 fold change. *P* value refers to a two-tailed, homoscedastic Student's *t* tests for unpaired samples. These data complement Fig. 4D. $n_{exp} = 4$. (**B**) Immunoblot of LRP8, GPX4, and vinculin (loading control) in NTC and LRP8ko clones (#1–2) derived from MDA-MB-468 breast cancer cells. (**C**) Well area covered by colonies formed by MDA-MB-468 NTC control cells and LRP8ko clones incubated for 7 days with mock medium or medium conditioned by NTC or SCDko MDA-MB-468 clones cultured as shown in Fig. 4F. *P* values refer to a one-way ANOVA test for unpaired samples with Dunnett's multiple comparisons test. $n_{exp} = 4$. Bars represent mean ± s.d. Representative images of wells with colonies are shown for each experimental condition.

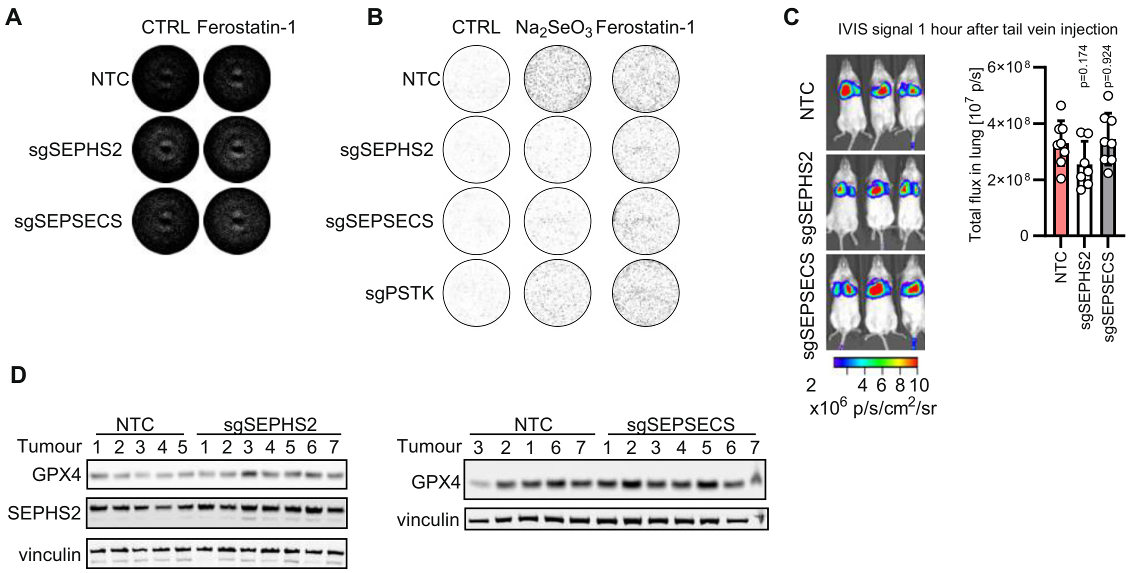

Figure EV4. Targeting selenocysteine biosynthesis impairs lung metastasis of TNBC.

(**A**) Representative images of the well area covered by cells at the end of the assays shown in Fig. 6C. (**B**) Representative images of the colony-forming assays shown in Fig. 6D. (**C**) IVIS pictures and quantification of lung metastasis burden 1 h after tail vein injection of 2×10^6 NTC, sgSEPHS2 or sgSEPSECS MDA-MB468 cells. The same mice are shown in Fig. 6H,I. *P* value refers to a one-way ANOVA test for unpaired samples with Dunnett's multiple comparisons test comparing to the NTC control. $n = 7$–8 female NSG mice as indicated by data points. One injected mouse of sgSEPHS2 group had to be culled due to husbandry reasons. (**D**) Immunoblot for GPX4, SEPHS2 and vinculin (loading control) in mammary tumours sampled 38 days after the transplantation of NTC, sgSEPHS2, or sgSEPSECS MDA-MB-468 cells. For each experimental group the lysates from 7 tumours were loaded as indicated.

 © The Author(s)
